# Scandium(III)-Enlarged Salen Complex-Catalyzed Asymmetric Michael Addition of Indoles to Enones

**DOI:** 10.3390/molecules30030459

**Published:** 2025-01-21

**Authors:** Ningning Li, Quanyu Ma, Jiaxi Xu

**Affiliations:** State Key Laboratory of Chemical Resource Engineering, Department of Organic Chemistry, College of Chemistry, Beijing University of Chemical Technology, Beijing 100029, China; 2022201047@buct.edu.cn (N.L.); 2022201019@buct.edu.cn (Q.M.)

**Keywords:** asymmetric Michael addition, indole, stereoselective control, schiff base ligand, scandium triflate

## Abstract

Salens are a class of important ligands and have been widely applied in asymmetric catalytic organic reactions. Enlarged salen-like ligands containing flexible chains were synthesized from L-phenylalanine, ethane/propanediamines, and salicylaldehydes, and successfully utilized in the scandium-catalyzed enantioselective Michael addition of indoles and enones (2-cinnamoylpyridine 1-oxides). The catalytic system demonstrates excellent reactivity and stereoselective control over electron-rich indole substrates with up to 99% yield and 99% enantiomeric excess. The enlarged Salen ligands with flexible and rigid combined linkers fit their coordination with large rare earth elements. Their coordination abilities were tuned by the electronic effect of substituents on their salicylaldehyde moiety, facilitating the construction of excellent chiral environments in the scandium(III)-catalyzed asymmetric Michael addition of indoles to 2-cinnamoylpyridine 1-oxides.

## 1. Introduction

**Figure 1:**
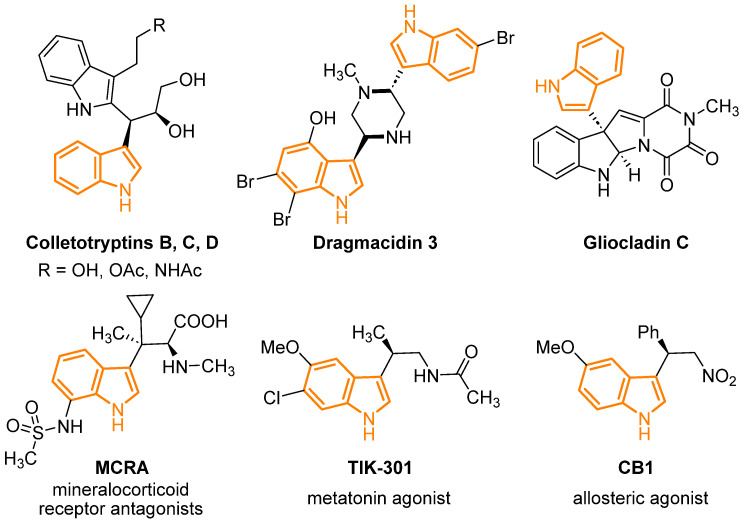
Biologically active 3-substituted indole derivatives.

Most indole compounds exhibit electron-rich properties and are prevalent in nature [1]. Optically active 3-substituted indole compounds serve as the core skeleton for numerous biologically active natural products and pharmaceutical agents, including the natural product Colletotryptins BD [2], Dragmacidin 3 [3], Gliocladin C [4], the mineralocorticoid receptor antagonist MCRA [5], the melatonin agonist TIK-301 [6], and the protein allosteric agonist CB1 [7], revealing the significance of these compounds (Figure 1).

In recent decades, the efficient synthesis of optically active 3-substituted indole compounds has been particularly appealing. The asymmetric Michael addition reaction is a significant method for constructing optically active molecules and has been widely utilized in organic synthesis [8,9,10]. Due to their electron-rich aromatic systems, indoles generally serve as excellent Michael donors [11], participating in the 1,4-addition reaction with α,β-unsaturated ketones [12,13]. α,β-unsaturated carbonyl derivatives are ideal Michael acceptors for enantioselective catalytic Michael reactions. Lewis acids have been typically employed to activate the carbon–carbon double bond of α,β-unsaturated ketones via reducing the energy of their lowest unoccupied molecular orbitals (LUMO). This activation mode facilitates the attack of the highest occupied molecular orbitals (HOMO) of electron-rich reagents, such as indoles [14].

**Scheme 1:**
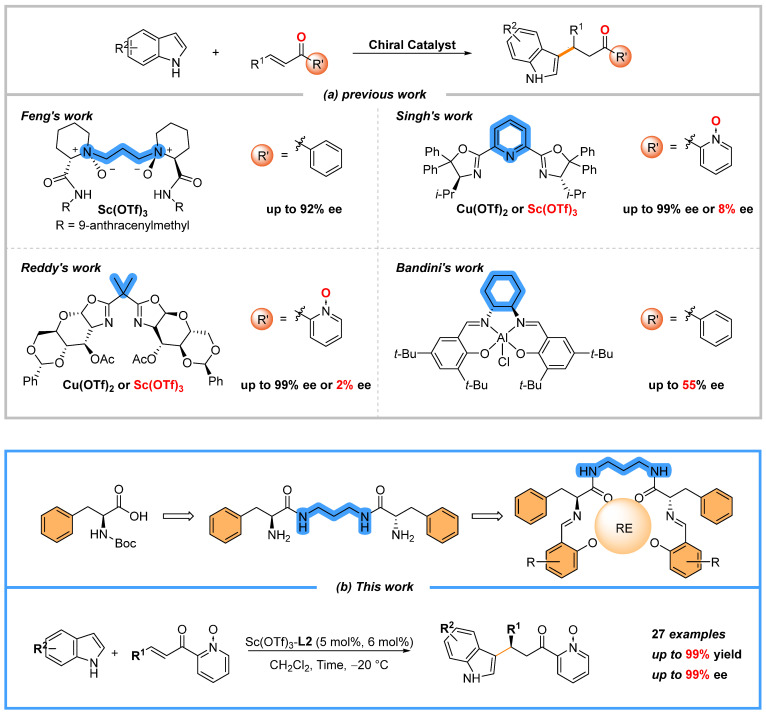
Asymmetric catalytic reactions of indoles with unsaturated enones.

Catalytic asymmetric Michael additions of indoles and enones have been successfully developed by using rare earth and transition metals in the presence of chiral ligands, including Feng’s diamine *N*,*N*-dioxide [15], PyBoxes [16], sugar-derived Boxes [17], and even aluminum–Salen complex [18] (Scheme 1, top). In comparison to transition metals, rare earth metal cations function as hard Lewis acids and display distinctive characteristics, including high oxophicility and elevated coordination numbers, and demonstrate excellent stereoselectivities in the catalytic asymmetric reactions involving enones [19,20]. The introduction of an auxiliary group at the α-position of the carbonyl group in unsaturated ketones would benefit coordination with rare earth metal cations, realizing superior chiral control. For example, 2-alk-2-enoylpyridines proceeded the PyBox–La(OTf)_3_-catalyzed asymmetric Diels–Alder cycloaddition with cyclopentadiene with excellent enantioselectivity [20]. China has abundant rare-earth metal resources. There is a demand to explore their application in catalytic asymmetric organic reactions. Herein, we present the La(OTf)_3_-enlarged Salen-complex catalyzed asymmetric Michael addition of indoles and 2-enoyl-pyridine *N*-oxides, which possess bidentate chelating sites to coordinate with rare earth metals, showing good to excellent stereoselectivities (Scheme 1, bottom). 

## 2. Results and Discussion

**Scheme 2:**
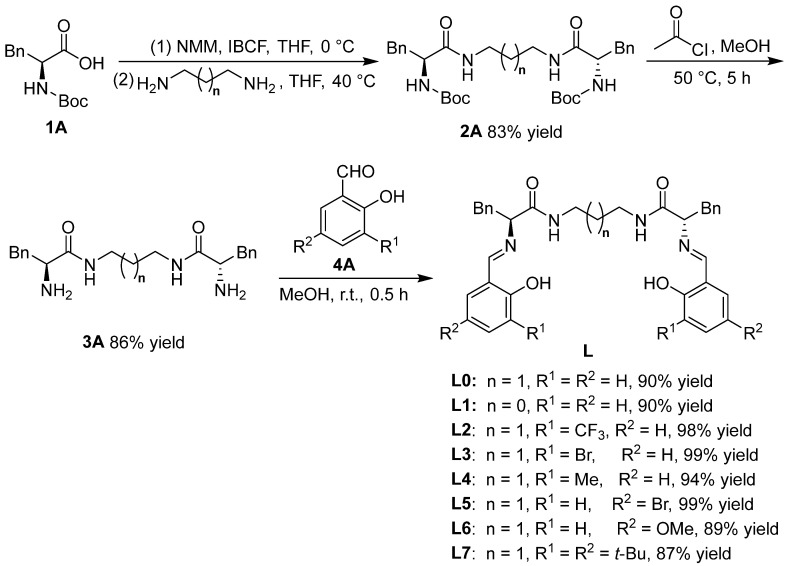
Synthesis of enlarged Salen ligands **L**.

To facilitate coordination with large rare-earth meat cations, various enantiopure enlarged Salen ligands **L** were prepared by referring to the reported procedure with a slight modification [21] (Scheme 2). In the enlarged Salen ligands, the original cyclohexane linker was displaced with linear linkers composed of rigid bisamides connected to flexible ethane-1,2-diyl or propane-1,3-diyl groups, enlarging their coordinate cavities to facilitate coordination with rare earth metal cations. The synthetic method was efficient. The desired ligands, **L0**–**L7**, were obtained in good to excellent yields.

To investigate the influence of the structure of enlarged Salen ligands with flexible and rigid combined linkers on the stereoselectivity in the 1,4-conjugate addition reaction of indoles and unsubstituted α,β-unsaturated ketones, the reaction of 6-methoxyindole (**1a**) and 2-cinnamoylpyridine 1-oxide (**2a**) was selected as a model reaction to evaluate the ligands (Table 1) and optimize the reaction conditions (Table 2). To ensure the formation of the ligand–Sc(OTf)_3_ complex, the selected ligand, Sc(OTf)_3_, and 2-cinnamoylpyridine 1-oxide (**2a**) were pre-mixed before adding 6-methoxyindole (**1a**). The results indicated that the ligand **L2,** which has a longer flexible chain consisting of three carbon atoms, and salicylaldehyde, possessing a strongly electron-withdrawing 3-trifluoromethyl group, exhibited the best stereoselective control (Table 1, entry 3). Notably, the displacement of the trifluoromethyl group with Br (**L3**) and CH_3_ (**L4**) groups significantly reduced the enantiomeric excess values (Table 1, entries 3–5). The ligands **L5** and **L6**, prepared from 5-bromo and 5-methoxysalicylaldehydes, presented low stereoselectivities (Table 1, entires 6 and 7). The ligand **L7**, derived from electron-rich and bulky 3,5-di(*tert*-butyl)salicyaldehyde, gave an excellent yield but low stereoselectivity (Table 1, entry 8). Thus, the ligand **L2** was chosen as the optimal ligand for further optimizations. These results suggest that the trifluoromethyl group in the ligand **L2** exerts an electron-withdrawing electronic effect, increasing the acidity of the phenolic group and favoring the formation of phenoxide, thereby enhancing the coordination with rare earth metal cations and forming the stable ligand–rare earth complex, which plays a critical role in controlling stereoselectivity. The stereostructure of product **3aa** was identified on the basis of chiral HPLC analysis on the same type of chiral column in comparison with the reported relative retention time.

The influence of different solvents on the stereoselectivity of the model reaction was investigated under the catalytic conditions of the ligand **L2**. The results revealed that dichloromethane is the best choice of solvent for stereochemical control (Table 2). Commercially available dichloromethane was also tested, affording the desired product in a low yield of 42% without enantioselectivity (Table 2, entry 10). Thus, the optimal conditions are identified as follows: **2a** (0.2 mmol), ligand **L2** (6 mol%), and Sc(OTf)_3_ (5 mol%) in dried DCM (2 mL) were stirred under N_2_ atmosphere at R.T. for 1 h. **1a** (0.24 mmol) was added and the resulting mixture was stirred at −20 °C for several hours, as determined by TLC monitoring.

With the optimal conditions in hand, the scope of indoles **1** was first studied (Table 3). Indoles **1a**–**1d**, with electron-rich and weak electron-poor 6-substituents, accomplished the reactions well, affording desired products **3aa**–**3da** in good yields (60–86%) and excellent enantioselectivities (94–99% ee) (Table 3, entries 1–4). However, indole **1e**, which has a strongly electron-withdrawing 6-ethoxycarbonyl group generated the desired product **3ea** in a low yield of 38% and 23% ee. This is possibly because the carboxylate group can also coordinate with the metal center, resulting in no activation of the enone moiety and leading to different stereocontrol in the reaction system (Table 3, entry 5). For 5-substituted indoles, electron-rich 5-methoxy- and 5-benzyloxyindoles **1f** and **1g** produced the corresponding products **3fa** and **3ga** in good to excellent yields (67% and 98%) and good enantioselectivities (88% ee and 78% ee) (Table 3, entries 6 and 7). In contrast, 5-chloro- and 5-fluoroindoles **1h** and **1i** gave rise to the expected products **3ha** and **3ia** in good yields (69% and 84%) and excellent enantioselectivities (98% ee and 93% ee) (Table 3, entries 8 and 9). However, 4-methoxyindole (**1j**) showed moderate yield (79%) and enantioselectivity (57% ee) due to steric hindrance (Table 3, entry 10). Both 1-methylindole (**1k**) and indole (**1m**) proceeded in the reaction, affording the expected products **3ka** and **3ma** in moderate yields (65% and 70%) but low enantioselectivities (38% ee and 51% ee) (Table 3, entries 11 and 13). However, 1-benzenesulfonylindole (**1l**) did not work due to low electron density, which significantly diminishes its nucleophilicity (Table 3, entry 12).

To further explore the applicable scope of unsaturated ketones **2**, *para*-substituted 2-cinnamoylpyridine 1-oxides, **2b**–**2g**, were first tested. Substrates **2b**, **2c**, **2f**, and **2g** with both strong electron-donating and withdrawing groups proceeded the reaction well, showing excellent reactivities (86–99% yields), but all of them exhibited moderate stereoselectivities (50–77% ee) (Table 4, entries 1–6). In comparison with *para*-substituted 2-cinnamoylpyridine 1-oxides **2b**–**2g**, their *ortho*-substituted partners, **2h**–**2k**, showed slightly low reactivities (74–95% yields), and low enantioselectivities (38–53% ee) (Table 4, entries 7–10). Fused 2-naphthyl- and heteroaryl 2-furyl substrates, **2l** and **2m**, also worked, giving the corresponding products **3al** and **3am** in 96% and 84% yields, respectively, but low stereocontrol (60% ee and 41% ee) (Table 4, entries 11 and 12). The results indicate that both steric and electronic effects of substitution govern stereoselectivity. Less steric furan-containing substrate **2m** exhibited poor stereoselectivity possibly because of the existence of additional coordinating oxygen atom in the furan ring (Table 4, entry 12).

**Figure 2:**
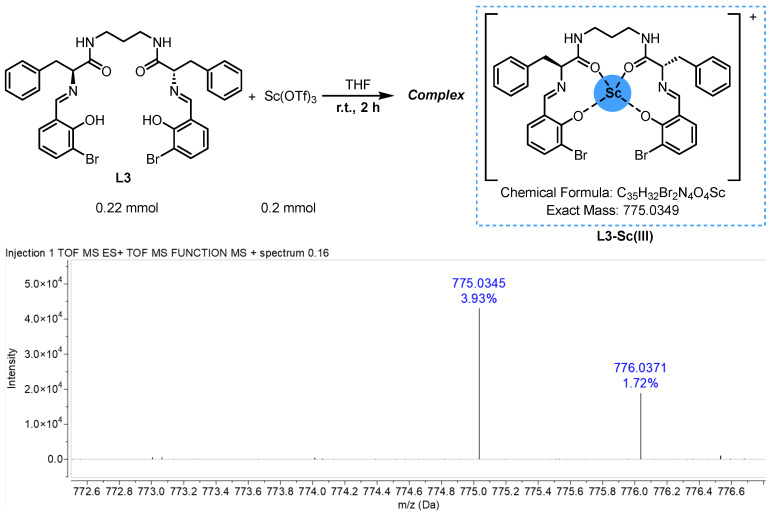
Preparation of **L3**–Sc(III) complex and its HRMS spectrum.

To identify the generated catalyst and to investigate the coordination behavior of ligands and rare earth metals, ligand **L3** (0.22 mmol, 161.6 mg) and Sc(OTf)_3_ (0.2 mmol, 98.4 mg) were added into anhydrous tetrahydrofuran (10 mL) under nitrogen atmosphere. The mixture was stirred at room temperature for 2 h. After the excess solvent was removed under vacuum, the resulting residue was washed with dry dichloromethane and subsequently recrystallized using a mixture of THF/DCM, yielding 152 mg of the complex. High-resolution mass spectrometric analysis suggests that its potential structure is **L3**-Sc(III), which provides an indirect evidence for the catalyst in the asymmetric Michael addition reaction (Figure 2).

**Figure 3:**
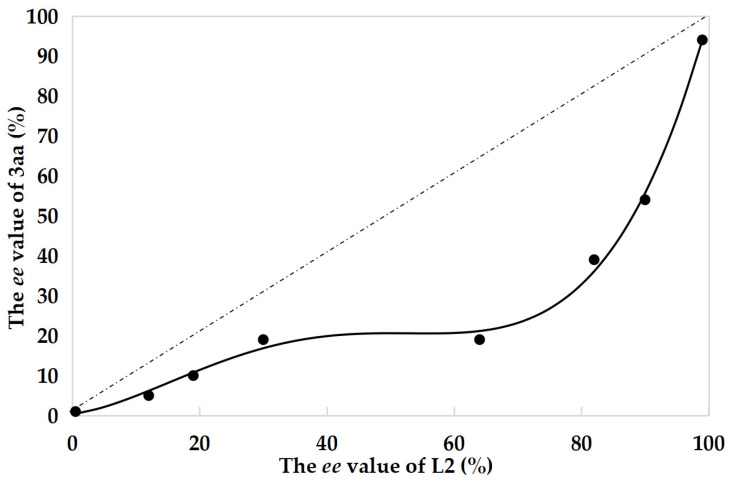
Stereoselectivity of product **3aa** as a function of the **L2** ee value.

Furthermore, we synthesized enantiomer of the ligand **L2** to prepare complexes of **L2** and Sc(OTf)_3_ with varying enantiomeric excess (ee) values, which were utilized to investigate their catalytic effect on the model reaction (Figure 3 and Table 5). The results illustrate that the chiral enlarged Salen ligand **L2**–Sc(III) complex acts as a catalyst for the reaction and displays a negative nonlinear effect. This observation suggests that the Sc(III)/L2 complex form diastereomeric dimer (or greater) structures with differing reactivity/solubility. The resulting reactive catalyst mixture reacts with diminished facial selectivity in the indole Michal addition. Understanding this phenomenon will enhance our ability to develop additional applications for these types of ligands in asymmetric contexts.

## 3. Materials and Methods

### 3.1. Materials and Instruments

Unless otherwise noted, all materials were purchased from commercial suppliers. Dichloromethane (DCM) and 1,2-dichloroethane (DCE) were refluxed over CaH_2_; Chloroform was dried by refluxed over anhydrous calcium carbonate; tetrahydrofuran (THF) and toluene (PhMe) were refluxed over lithium aluminum hydride. The solvents were freshly distilled prior to use. Methyl *tert*-butyl ether (MTBE), Ethyl acetate (EA), and CH_3_CN are all commercially available anhydrous solvents. All solvents were dried and then further dried over 4 Å molecular sieves for 12 h. ^1^H NMR spectra were recorded in CDCl_3_ or DMSO-*d*_6_ on a Bruker 400 NMR spectrometer (Bruker Corporation, Billerica, MA USA) usually with TMS as an internal standard. The chemical shifts were recorded in ppm relative to tetramethylsilane (TMS) and with the solvent resonance as the internal standard. The data are reported as follows: chemical shift, multiplicity: s (singlet), d (doublet), t (triplet), q (quartet), dd (double doublet), quin (quintet), m (multiplet), and br (broad), coupling constants (Hz), integration. The ^13^C NMR (101 MHz) and ^19^F NMR (377 MHz) data were collected in CDCl_3_ or DMSO-*d*_6_ on the same instrument with complete proton decoupling. Unless otherwise noted, all the solvents were purified by usual methods prior to use. Column chromatography was performed on silica gel (normal phase, 200–300 mesh) from Anhui Liangchen Silicon Material Co., Ltd. CH_2_Cl_2_ and MeOH (50:1, *v*/*v*) were used as eluent. The reactions were monitored using thin-layer chromatography (TLC) on GF254 silica gel plates (0.2 mm) from Anhui Liangchen Silicon Material Co., Ltd. (Liuan, Anhui province, China) The plates were visualized by UV light. The specific rotations were measured on an Anton Paar MCP500 polarimeter (Anton Paar GmbH—Headquarters, Graz, Austria) and reported as follows: ${\left[\alpha \right]}_{D}^{T}$ (c: g/100 mL, in solvent). The HRMS measurements were carried out on an Agilent LC/MSD TOF mass spectrometer (Agilent Technologies Inc. Palo Alto, CA, USA). The melting points were determined using a micro-melting point instrument, model SGW^®^ X4A, manufactured by Shanghai INESA Physico-Optical Instrument Co., Ltd. (Shanghai, China). The enantiomeric excesses were determined by chiral HPLC analysis using an Agilent 1260 LC instrument (Agilent Technologies Inc., Palo Alto, CA, USA) with Daicel Chiralcel AD-H, OD-H, or AS-H column with a mixture of isopropyl alcohol and hexane as eluents.

### 3.2. General Procedure for the Synthesis of Ligands ***L***

The ligands were prepared according to the reported procedure with slight modification [21,22].

In a 500 mL round-bottom flask, *N*-Boc-*L*-phenylalanine (**1A**) (13.27 g, 50 mmol) and tetrahydrofuran (200 mL) were added and the mixture was cooled in an ice-water bath at 0 °C. *N*-Methylmorpholine (NMM) (5.50 mL, 50 mmol) and isobutyl chloroformate (IBCF) (6.49 mL, 50 mmol) were then added into the reaction mixture under stirring. After stirring for 30 min, ethane-1,2-diamine or propane-1,3-diamine (50 mmol) was added, and the reaction mixture was warmed to 40 °C in an oil bath. The reaction mixture was stirred at the same temperature for 5 h. After the removal of the solvent, the resulting residue was extracted with dichloromethane. The combined organic phase was washed sequentially with hydrochloric acid (2 M, 60 × 3 mL) and saturated brine, and dried over anhydrous Na_2_SO_4_. After the solvent was evaporated in vacuum, a pure product **2A** was obtained with a yield of 83%.

The product **2A** obtained from the previous step was completely dissolved in methanol (100 mL) in a 250 mL round-bottom flask and the flask was placed in an ice-water bath at 0 °C, to which acetyl chloride (17.84 mL, 250 mmol) was added dropwise. After the ice-water bath was removed, the reaction mixture was allowed to warm to 50 °C in an oil bath and the reaction mixture was stirred at the same temperature for 5 h. After the removal of the solvent, the residue was dissolved in dichloromethane. The solution was washed with ammonia solution (25%~28%, *w*/*w*, 10 × 2 mL) and saturated brine sequentially, and dried over anhydrous Na_2_SO_4_. After the solvent was evaporated under vacuum conditions, the resulting residue was purified by recrystallization in petroleum ether, yielding product **3A** in 86% yield.

**3A** (2 mmol) was dissolved in 20 mL of methanol, then salicylaldehyde (8 mmol) was added (noncommercially available salicylaldehydes were prepared referring to the reported procedure [23]). The resulting mixture was stirred at room temperature for 0.5 h. Upon completion of the reaction, the solvent was evaporated under reduced pressure. The resulting crude product was then filtered and washed using suction to remove unreacted salicylaldehyde. Finally, the product was recrystallized from a mixture of DCM/MeOH, yielding yellow crystalline product ligand **L**.

(2*S*,2′*S*)-*N*,*N*′-(Propane-1,3-diyl)bis(2-(((*E*)-2-hydroxybenzylidene)amino)-3-phenylpropanamide) (**L0**)

Yellow crystals, 1.02 g, yield 90%; m.p. 143.1−144.5 °C. ^1^H NMR (400 MHz, CDCl_3_) δ 12.36 (s, 2H), 7.95 (s, 2H), 7.33 (ddd, *J* = 8.6, 7.3, 1.7 Hz, 2H), 7.26–7.12 (m, 12H), 6.98 (dd, *J* = 8.4, 1.1 Hz, 2H), 6.87 (td, *J* = 7.5, 1.1 Hz, 2H), 6.57 (t, *J* = 6.3 Hz, 2H), 4.03 (dd, *J* = 8.6, 3.8 Hz, 2H), 3.36 (dd, *J* = 13.5, 3.8 Hz, 2H), 3.22–3.05 (m, 6H), 1.55 (quin, *J* = 6.4 Hz, 2H). ^13^C NMR (101 MHz, CDCl_3_) δ 171.2, 167.6, 160.6, 137.0, 133.1, 132.1, 129.8, 128.4, 126.8, 119.1, 118.4, 117.0, 75.5, 40.9, 35.9, 29.6.,29.4 HRMS (ESI): *m*/*z* calcd for C_35_H_37_N_4_O_4_^+^ [M + H]^+^: 577.2809, found: 577.2813.

(2*S*,2′*S*)-*N*,*N*′-(Ethane-1,2-diyl)bis(2-(((*E*)-2-hydroxy-3-(trifluoromethyl)benzylidene)amino)-3-phenylpropanamide) (**L1**) [21]

Yellow crystals, 1.26 g, yield 90%; m.p. 110.3–112.3 °C. ^1^H NMR (400 MHz, CDCl_3_) δ 13.35 (s, 2H), 7.88 (s, 2H), 7.60 (dd, *J* = 7.8, 1.6 Hz, 2H), 7.26–7.09 (m, 12H), 6.90 (t, *J* = 7.7 Hz, 2H), 6.72 (t, *J* = 4.3 Hz, 2H), 4.07 (dd, *J* = 9.2, 3.8 Hz, 2H), 3.41 (ddd, *J* = 13.7, 6.1, 3.6 Hz, 6H), 3.05 (dd, *J* = 13.6, 9.3 Hz, 2H). ^13^C NMR (101 MHz, CDCl_3_) δ 171.6, 166.9, 159.0, 136.7, 135.7, 130.3, 129.7, 128.6, 126.9, 119.1, 118.3, 77.24, 75.3, 40.8, 40.4. ^19^F NMR (377 MHz, CDCl_3_) δ −2.41.

(2*S*,2′*S*)-*N*,*N*′-(Propane-1,3-diyl)bis(2-(((*E*)-2-hydroxy-3-(trifluoromethyl)benzylidene)amino)-3-phenylpropanamide) (**L2**) [21]

Yellow crystals, 1.40 g, yield 98%; m.p. 149.6–151.2 °C. ^1^H NMR (400 MHz, CDCl_3_) δ 13.49 (s, 2H), 7.96 (s, 2H), 7.61 (dd, *J* = 7.9, 1.6 Hz, 2H), 7.31–7.09 (m, 12H), 6.91 (t, *J* = 7.7 Hz, 2H), 6.77 (t, *J* = 6.4 Hz, 2H), 4.07 (dd, *J* = 9.0, 4.0 Hz, 2H), 3.41 (dd, *J* = 13.6, 4.0 Hz, 2H), 3.24–3.14 (m, 4H), 3.06 (dd, *J* = 13.6, 9.0 Hz, 2H), 1.59 (quin, *J* = 6.3 Hz, 2H). ^13^C NMR (101 MHz, CDCl_3_) δ 170.8, 166.8, 159.2, 136.8, 135.7, 130.3, 129.7, 128.6, 126.9, 119.2, 118.2, 77.4, 77.1, 76.7, 75.3, 40.7, 35.8, 29.3. ^19^F NMR (376 MHz, CDCl_3_) δ −62.57.

(2*S*,2′*S*)-*N*,*N*′-(Propane-1,3-diyl)bis(2-(((*E*)-3-bromo-2-hydroxybenzylidene)amino)-3-phenylpropanamide) (**L3**)

Yellow crystals, 1.45 g, yield 99%; m.p. 109.4.1–110.6 °C. ^1^H NMR (400 MHz, CDCl_3_) δ 13.43 (s, 2H), 7.91 (s, 2H), 7.57 (dd, *J* = 7.9, 1.5 Hz, 2H), 7.28–7.05 (m, 12H), 6.76 (q, *J* = 6.5 Hz, 4H), 4.09 (dd, *J* = 9.0, 3.8 Hz, 2H), 3.43 (dd, *J* = 13.6, 3.8 Hz, 2H), 3.20 (tt, *J* = 6.6, 3.8 Hz, 4H), 3.08 (dd, *J* = 13.6, 9.0 Hz, 2H), 1.60 (quin, *J* = 6.3 Hz, 2H). ^13^C NMR (101 MHz, CDCl_3_) δ 170.8, 166.8, 157.5, 136.8, 136.3, 131.3, 129.7, 128.6, 126.9, 119.9, 119.2, 110.8, 75.1, 40.8, 36.0, 29.4. HRMS (ESI): *m*/*z* calcd for C_35_H_35_Br_2_N_4_O_4_^+^ [M + H]^+^: 733.1020, found: 733.1024.

(2*S*,2′*S*)-*N*,*N*′-(Propane-1,3-diyl)bis(2-(((*E*)-2-hydroxy-3-methylbenzylidene)amino)-3-phenylpropanamide) (**L4**)

Yellow crystals, 1.14 g, yield 94%; m.p. 135.9–138.6 °C. ^1^H NMR (400 MHz, CDCl_3_) δ 12.59 (s, 2H), 7.93 (s, 2H), 7.25–7.12 (m, 12H), 6.96 (dd, *J* = 7.7, 1.7 Hz, 2H), 6.77 (t, *J* = 7.5 Hz, 2H), 6.63–6.48 (m, 2H), 4.02 (dd, *J* = 8.8, 3.7 Hz, 2H), 3.38 (dd, *J* = 13.5, 3.7 Hz, 2H), 3.18 (tq, *J* = 11.1, 6.3 Hz, 4H), 3.08 (dd, *J* = 13.5, 8.7 Hz, 2H), 2.29 (s, 6H), 1.56 (quin, *J* = 6.5 Hz, 2H). ^13^C NMR (101 MHz, CDCl_3_) δ 171.3, 167.8, 158.9, 137.1, 134.2, 129.9, 129.8, 128.5, 126.8, 126.0, 118.7, 117.7, 75.4, 40.9, 36.0, 29.6, 15.5. HRMS (ESI): *m*/*z* calcd for C_37_H_41_N_4_O_4_^+^ [M + H]^+^: 605.3122, found: 6053.3124.

(2*S*,2′*S*)-*N*,*N*′-(Propane-1,3-diyl)bis(2-(((*E*)-5-bromo-2-hydroxybenzylidene)amino)-3-phenylpropanamide) (**L5**)

Yellow crystals, 1.45 g, yield 99%; m.p. 140.3–141.9 °C. ^1^H NMR (400 MHz, CDCl_3_) δ 12.39 (s, 2H), 7.89 (s, 2H), 7.42 (dd, *J* = 8.9, 2.5 Hz, 2H), 7.31–7.18 (m, 8H), 7.17–7.06 (m, 4H), 6.90 (d, *J* = 8.8 Hz, 2H), 6.64 (t, *J* = 6.4 Hz, 2H), 4.05 (dd, *J* = 8.7, 3.8 Hz, 2H), 3.38 (dd, *J* = 13.5, 3.8 Hz, 2H), 3.18 (ddd, *J* = 21.0, 13.6, 6.9 Hz, 4H), 3.14–3.02 (m, 2H), 1.58 (quin, *J* = 6.2 Hz, 2H). ^13^C NMR (101 MHz, CDCl_3_) δ 171.0, 166.3, 159.6, 136.8, 135.8, 134.1, 129.7, 128.5, 126.9, 119.8, 119.1, 110.6, 75.4, 40.8, 35.8, 29.4. HRMS (ESI): *m*/*z* calcd for C_35_H_35_Br_2_N_4_O_4_^+^ [M + H]^+^: 733.1020, found: 733.1024.

(2*S*,2′*S*)-*N*,*N*′-(Propane-1,3-diyl)bis(2-(((*E*)-2-hydroxy-5-methoxybenzylidene)amino)-3-phenylpropanamide) (**L6**)

Yellow crystals, 1.13 g, yield 89%; m.p. 147.4–148.8 °C. ^1^H NMR (400 MHz, CDCl_3_) δ 11.91 (s, 2H), 7.89 (s, 2H), 7.25–7.11 (m, 10H), 6.96–6.85 (m, 4H), 6.68–6.56 (m, 4H), 4.02 (dd, *J* = 8.6, 3.8 Hz, 2H), 3.72 (s, 6H), 3.35 (dd, *J* = 13.5, 3.8 Hz, 2H), 3.17 (qd, *J* = 6.4, 3.3 Hz, 4H), 3.09 (dd, *J* = 13.5, 8.7 Hz, 2H), 1.55 (quin, *J* = 6.4 Hz, 2H). ^13^C NMR (101 MHz, CDCl_3_) δ 171.2, 167.3, 154.7, 152.3, 137.0, 129.8, 128.5, 126.8, 120.3, 118.1, 117.8, 115.3, 75.5, 55.9, 40.9, 35.9, 29.6. HRMS (ESI): *m*/*z* calcd for C_37_H_41_N_4_O_6_^+^ [M + H]^+^: 637.3021, found: 637.3022.

(2*S*,2′*S*)-*N*,*N*′-(Propane-1,3-diyl)bis(2-(((*E*)-3,5-di-*tert*-butyl-2-hydroxybenzylidene)amino)-3-phenylpropanamide) (**L7**)

Yellow crystals, 1.39 g, yield 87%; m.p. 193.4–195.2 °C. ^1^H NMR (400 MHz, CDCl_3_) δ 12.79 (s, 2H), 8.04 (s, 2H), 7.41 (d, *J* = 2.4 Hz, 2H), 7.25–7.14 (m, 10H), 6.97 (d, *J* = 2.4 Hz, 2H), 6.52 (t, *J* = 6.5 Hz, 2H), 4.03 (dd, *J* = 8.3, 3.9 Hz, 2H), 3.34 (dd, *J* = 13.6, 4.0 Hz, 2H), 3.13 (ddd, *J* = 13.8, 9.5, 5.2 Hz, 6H), 1.46 (s, 20H), 1.27 (s, 18H). ^13^C NMR (101 MHz, CDCl_3_) δ 171.3, 168.6, 157.7, 140.6, 137.2, 136.8, 129.8, 128.4, 127.9, 126.7, 117.6, 75.4, 41.0, 35.9, 35.1, 34.2, 31.5, 29.5. HRMS (ESI): *m*/*z* calcd for C_51_H_69_N_4_O_4_^+^ [M + H]^+^: 801.5313, found: 801.5314.

### 3.3. General Procedure for the Asymmetric Catalytic Michael Addition of Indoles ***1*** and Enones ***2***

A solution of ligand **L2** (8.6 mg, 0.012 mmol), *trans*-2-enoyl pyridine 1-oxide (0.20 mmol), and Sc(OTf)_3_ (4.9 mg, 0.01 mmol) in dry chloroform (2 mL) was stirred at room temperature for 1 h under nitrogen atmosphere at room temperature. The reaction mixture was cooled to −20 °C and stirred for 10 min. Indole (0.24 mmol) was added, and the reaction mixture was stirred at −20 °C until the completion of the reaction (monitored by TLC). The mixture was concentrated in vacuo and the residue was purified over silica gel by column chromatography (methanol/dichloromethane 1:50, *v*/*v* as eluent) to afford product **3**.

(*R*)-2-(3-(6-Methoxy-1*H*-indol-3-yl)-3-phenylpropanoyl)pyridine 1-oxide (**3aa**)

Colorless crystals, 48 mg, yield 65%; m.p. 167.9–169.1 °C. *ee* 96%, HPLC analysis: chiralpak AD-H (*i*-PrOH/hexane = 40:60, *v*/*v*, 1.0 mL/min, 254 nm) major *t*_R_ = 15.57 min and minor *t*_S_ = 18.27 min. ^1^H NMR (400 MHz, CDCl_3_) δ 8.06 (d, *J* = 6.4 Hz, 1H), 7.90 (brs, 1H), 7.23–7.11 (m, 6H), 7.08–6.99 (m, 3H), 6.89 (dd, *J* = 2.4, 1.0 Hz, 1H), 6.70 (d, *J* = 2.2 Hz, 1H), 6.57 (dd, *J* = 8.7, 2.3 Hz, 1H), 4.80 (t, *J* = 7.8 Hz, 1H), 3.98 (dd, *J* = 16.4, 7.6 Hz, 1H), 3.86 (dd, *J* = 16.4, 8.0 Hz, 1H), 3.71 (s, 3H). ^13^C{1H} NMR (101 MHz, CDCl_3_) δ 197.2, 153.8, 147.1, 143.7, 140.1, 131.6, 128.4, 127.9, 127.5, 127.1, 126.5, 126.4, 125.6, 122.4, 118.5, 112.2, 111.7, 101.3, 55.8, 49.0, 38.6. HRMS (ESI): *m*/*z* Calcd for C_23_H_20_N_2_O_3_Na^+^ [M + Na]^+^: 395.1366, found: 395.1370.

(*R*)-2-(3-(6-Methyl-1*H*-indol-3-yl)-3-phenylpropanoyl)pyridine 1-oxide (**3ba**)

Yellow crystals, 53 mg, yield 74%; m.p. 185.9–189.4 °C. *ee* 94%, HPLC analysis: chiralpak AD-H (*i*-PrOH/hexane = 40:60, *v*/*v*, 1.0 mL/min, 230 nm) major *t*_R_ = 13.48 min and minor *t*_R_ = 16.79 min. ^1^H NMR (400 MHz, DMSO-*d*_6_) δ 10.70 (d, *J* = 2.4 Hz, 1H), 8.32 (d, *J* = 6.4 Hz, 1H), 7.51 (ddd, *J* = 7.5, 6.5, 2.2 Hz, 1H), 7.38–7.29 (m, 3H), 7.25–7.08 (m, 7H), 6.71 (dd, *J* = 8.2, 1.5 Hz, 1H), 4.76 (t, *J* = 7.7 Hz, 1H), 3.99 (dd, *J* = 16.7, 8.0 Hz, 1H), 3.80 (dd, *J* = 16.7, 7.4 Hz, 1H), 2.34 (s, 3H). ^13^C NMR (101 MHz, DMSO-*d*_6_) δ 197.8, 146.8, 145.1, 140.5, 137.3, 130.5, 128.8, 128.6, 128.1, 126.4, 126.3, 126.2, 124.7, 121.8, 120.5, 118.8, 117.6, 111.6, 48.6, 38.3, 21.8. HRMS (ESI): *m*/*z* Calcd for C_23_H_20_N_2_NaO_2_^+^ [M + Na]^+^: 379.1417, found: 379.1427.

(*R*)-2-(3-(6-Bromo-1*H*-indol-3-yl)-3-phenylpropanoyl)pyridine 1-oxide (**3ca**)

Brown crystals, 50 mg, yield 60%; m.p. 198.7–201.7 °C. *ee* 98%, HPLC analysis: chiralpak AD-H (*i*-PrOH/hexane = 30:70, *v*/*v*, 1.0 mL/min, 254 nm) major *t*_R_ = 10.97 min and minor *t*_S_ = 12.26 min. ^1^H NMR (400 MHz, DMSO-*d*_6_) δ 11.05 (d, *J* = 2.6 Hz, 1H), 8.39–8.27 (m, 1H), 7.57–7.46 (m, 2H), 7.38–7.17 (m, 8H), 7.13 (t, *J* = 7.4 Hz, 1H), 7.01 (dd, *J* = 8.5, 1.8 Hz, 1H), 4.78 (t, *J* = 7.7 Hz, 1H), 4.01 (dd, *J* = 16.9, 8.1 Hz, 1H), 3.79 (dd, *J* = 16.9, 7.2 Hz, 1H). ^13^C NMR (101 MHz, DMSO-*d*_6_) δ 197.6, 146.8, 144.8, 140. 6, 137.7, 128.9, 128.7, 128.0, 126.6, 126.3, 126.3, 125.8, 123.7, 121.6, 120.8, 118.1, 114.4, 114.3, 48.5, 37.9. HRMS (ESI): *m*/*z* Calcd for C_22_H_17_N_2_O_2_BrNa^+^ [M + Na]^+^: 443.0366, found: 443.0432.

(*R*)-2-(3-(6-Fluoro-1*H*-indol-3-yl)-3-phenylpropanoyl)pyridine 1-oxide (**3da**)

Colorless crystals, 62 mg, yield 86%; m.p.147.4–149.5 °C. *ee* 99%, HPLC analysis: chiralpak AD-H (*i*-PrOH/hexane = 40:60, *v*/*v*, 1.0 mL/min, 254 nm) major *t*_R_ = 9.42 min and minor *t*_S_ = 10.38 min. ^1^H NMR (400 MHz, CDCl_3_) δ 8.16 (d, *J* = 6.5 Hz, 1H), 7.98 (brs, 1H), 7.31–7.25 (s, 5H), 7.24–7.17 (m, 4H), 7.14 (tdd, *J* = 7.9, 3.7, 1.3 Hz, 2H), 7.03 (dd, *J* = 9.7, 2.5 Hz, 1H), 6.86 (td, *J* = 9.0, 2.5 Hz, 1H), 4.87 (t, *J* = 7.7 Hz, 1H), 4.08 (dd, *J* = 16.6, 7.6 Hz, 1H), 3.93 (dd, *J* = 16.7, 7.8 Hz, 1H). ^13^C NMR (101 MHz, DMSO-*d*_6_) δ 197.7, 160.4, 158.1, 146.8, 144.8, 140.5, 136.7, 136.6, 128.9, 128.7, 128.1, 126.6, 126.3, 123.6, 123.2, 120.1, 120.0, 118.0, 107.4, 107.1, 97.9, 97.6, 48.5, 38.1. ^19^F NMR (376 MHz, CDCl_3_) δ −124.39. HRMS (ESI): *m*/*z* Calcd for C_22_H_17_N_2_O_2_FNa^+^ [M + Na]^+^: 383.1166, found: 383.1169.

(*R*)-2-(3-(6-(Methoxycarbonyl)-1*H*-indol-3-yl)-3-phenylpropanoyl)pyridine 1-oxide (**3ea**)

Colorless crystals, 30 mg, yield 38%; *ee* 23%, m.p. 194.5–196.3 °C. HPLC analysis: chiralpak AD-H (*i*-PrOH/hexane = 30:70, *v*/*v*, 1.0 mL/min, 230 nm) major *t*_R_ = 39.17 min and minor *t*_S_ = 54.22 min. ^1^H NMR (400 MHz, DMSO-*d*_6_) δ 11.35 (s, 1H), 8.32 (d, *J* = 6.4 Hz, 1H), 7.99 (s, 1H), 7.64–7.40 (m, 4H), 7.37–7.28 (m, 3H), 7.27–7.17 (m, 3H), 7.16–7.08 (m, 1H), 4.82 (t, *J* = 7.7 Hz, 1H), 4.03 (dd, *J* = 16.9, 8.1 Hz, 1H), 3.83 (s, 4H). ^13^C NMR (101 MHz, DMSO-*d*_6_) δ 197.6, 167.7, 146.8, 144.8, 140.6, 136.0, 130.2, 128.9, 128.7, 128.1, 126.7, 126.6, 126.3, 126.3, 122.5, 119.5, 118.9, 118.4, 113.9, 52.2, 48.5, 37.9. HRMS (ESI): *m*/*z* Calcd for C_24_H_20_N_2_O_4_Na^+^ [M + Na]^+^: 423.1315, found: 423.1320.

(*R*)-2-(3-(5-Methoxy-1*H*-indol-3-yl)-3-phenylpropanoyl)pyridine 1-oxide (**3fa**) [17]

Yellow crystals, 50 mg, yield 67%; *ee* 88%, m.p. 169.1–171.9 °C. HPLC analysis: chiralpak AD-H (*i*-PrOH/hexane = 40:60, *v*/*v*, 1.0 mL/min, 254 nm) major *t*_R_ = 16.29 min and minor *t*_S_ = 19.35 min. ^1^H NMR (400 MHz, CDCl_3_) δ 8.17 (d, *J* = 6.5 Hz, 1H), 8.04 (s, 1H), 7.38–7.30 (m, 2H), 7.30–7.10 (m, 7H), 7.09–7.05 (m, 1H), 6.88 (d, *J* = 2.4 Hz, 1H), 6.80 (dd, *J* = 8.8, 2.4 Hz, 1H), 4.90 (t, *J* = 7.8 Hz, 1H), 4.11 (dd, *J* = 16.4, 7.6 Hz, 1H), 3.96 (dd, *J* = 16.4, 8.0 Hz, 1H), 3.76 (s, 3H). ^13^C NMR (101 MHz, CDCl_3_) δ 197.2, 153.8, 147.1, 143.7, 140.1, 131.6, 128.4, 127.9, 127.5, 127.1, 126.5, 126.4, 125.6, 122.4, 118.5, 112.2, 111.7, 101.3, 55.8, 49.0, 38.6.

(*R*)-2-(3-(5-(Benzyloxy)-1*H*-indol-3-yl)-3-phenylpropanoyl)pyridine 1-oxide (**3ga**)

Yellow crystals, 88 mg, yield 98%; m.p. 126.1.9–128.0 °C. *ee* 78%, HPLC analysis: chiralpak AD-H (*i*-PrOH/hexane = 40:60, *v*/*v*, 1.0 mL/min, 254 nm) major *t*_R_ = 24.97 min and minor *t*_S_ = 29.80 min. ^1^H NMR (400 MHz, CDCl_3_) δ 8.26 (brs, 1H), 7.97 (dd, *J* = 6.4, 1.0 Hz, 1H), 7.34–7.27 (m, 2H), 7.27–7.15 (m, 5H), 7.11–6.99 (m, 5H), 6.96–6.83 (m, 4H), 6.72 (dd, *J* = 8.8, 2.4 Hz, 1H), 4.86 (d, *J* = 1.5 Hz, 2H), 4.75 (t, *J* = 7.7 Hz, 1H), 3.96 (dd, *J* = 16.5, 7.5 Hz, 1H), 3.83 (dd, *J* = 16.4, 8.0 Hz, 1H). ^13^C NMR (101 MHz, DMSO-*d*_6_) δ 197.8, 152.3, 146.9, 144.9, 140.5, 138.3, 132.2, 128.8, 128.8, 128.7, 128.1, 128.1, 127.1, 126.4, 126.3, 126.2, 123.3, 117.6, 112.5, 112.1, 102.8, 70.3, 48.4, 38.2. HRMS (ESI): *m*/*z* Calcd for C_29_H_24_N_2_O_3_Na^+^ [M + Na]^+^: 471.1679, found: 471.1680.

(*R*)-2-(3-(5-Chloro-1*H*-indol-3-yl)-3-phenylpropanoyl)pyridine 1-oxide (**3ha**) [17]

Orange-red crystals, 52 mg, yield 69%; m.p. 196.9–201.9 °C. *ee* 98%, HPLC analysis: chiralpak AD-H (*i*-PrOH/hexane = 40:60, *v*/*v*, 1.0 mL/min, 230 nm) major *t*_R_ = 10.06 min and minor *t*_s_ = 12.04 min, ^1^H NMR (400 MHz, DMSO-*d*_6_) δ 11.11 (s, 1H), 8.33 (d, *J* = 6.4 Hz, 1H), 7.52 (td, *J* = 7.0, 2.2 Hz, 1H), 7.45–7.29 (m, 6H), 7.27–7.19 (m, 3H), 7.14 (t, *J* = 7.3 Hz, 1H), 7.03 (dd, *J* = 8.6, 2.1 Hz, 1H), 4.79 (t, *J* = 7.7 Hz, 1H), 4.04 (dd, J = 16.9, 8.2 Hz, 1H), 3.79 (dd, *J* = 16.9, 7.2 Hz, 1H). ^13^C NMR (101 MHz, DMSO-*d*_6_) δ 197.6, 146.8, 144.8, 140.6, 135.3, 128.9, 128.7, 128.1, 127.9, 126.6, 126.4, 126.3, 124.5, 123.5, 121.5, 118.3, 117.7, 113.4, 48.5, 37.9.

(*R*)-2-(3-(5-Fluoro-1*H*-indol-3-yl)-3-phenylpropanoyl)pyridine 1-oxide (**3ia**) [17]

Colorless crystals, 61 mg, yield 84%; m.p. 198.6–202.2 °C. *ee* 93%, HPLC analysis: chiralpak AD-H (*i*-PrOH/hexane = 40:60, *v*/*v*, 1.0 mL/min, 254 nm) major *t*_R_ = 8.09 min and minor *t*_S_ = 8.84 min, ^1^H NMR (400 MHz, DMSO-*d*_6_) δ 10.99 (d, *J* = 2.6 Hz, 1H), 8.41–8.28 (m, 1H), 7.52 (ddd, *J* = 7.6, 6.5, 2.1 Hz, 1H), 7.42–7.27 (m, 5H), 7.26–7.17 (m, 3H), 7.16–7.05 (m, 2H), 6.86 (td, *J* = 9.1, 2.5 Hz, 1H), 4.75 (t, *J* = 7.7 Hz, 1H), 4.02 (dd, *J* = 16.8, 8.1 Hz, 1H), 3.79 (dd, *J* = 16.8, 7.4 Hz, 1H). ^13^C NMR (101 MHz, DMSO-*d*_6_) δ 197.7, 158.1, 155.8, 146.8, 144.8, 140.6, 133.5, 128.9, 128.7, 128.1, 127.0, 126.9, 126.6, 126.3, 126.3, 124.7, 118.1, 112.8, 112.8, 109.7, 109.5, 103.8, 103.6, 48.4, 38.0. ^19^F NMR (377 MHz, DMSO-*d*_6_) δ −125.29.

(*R*)-2-(3-(4-Methoxy-1*H*-indol-3-yl)-3-phenylpropanoyl)pyridine 1-oxide (**3ja**)

Yellow crystals, 59 mg, yield 79%; m.p. 81.7–85.8 °C. *ee* 57%, HPLC analysis: chiralpak AD-H (*i*-PrOH/hexane = 30:70, *v*/*v*, 1.0 mL/min, 254 nm) major *t*_R_ = 8.46 min and minor *t*_S_ = 9.19 min. ^1^H NMR (400 MHz, CDCl_3_) δ 8.31 (brs, 1H), 8.14 (d, *J* = 6.5 Hz, 1H), 7.38–7.30 (m, 2H), 7.22 (dd, *J* = 9.2, 5.9 Hz, 3H), 7.15–7.09 (m, 1H), 7.08–6.99 (m, 3H), 6.93–6.79 (m, 2H), 6.40 (d, *J* = 7.8 Hz, 1H), 5.32 (d, *J* = 8.1 Hz, 1H), 4.02 (dd, *J* = 7.9, 3.8 Hz, 2H), 3.78 (s, 3H). ^13^C NMR (101 MHz, CDCl_3_) δ 197.6, 154.7, 147.4, 144.8, 139.9, 138.1, 128.1, 128.1, 127.3, 126.3, 125.9, 125.6, 122.9, 120.5, 119.5, 116.8, 104.4, 99.6, 54.9, 49.8, 39.2. HRMS (ESI): *m*/*z* Calcd for C_23_H_20_N_2_O_3_Na^+^ [M + Na]^+^: 395.1366, found: 395.1363.

(*R*)-2-(3-(1-Methyl-1*H*-indol-3-yl)-3-phenylpropanoyl)pyridine 1-oxide (**3ka**) [17]

Yellow crystals, 46 mg, yield 65%; m.p. 42.9–44.8 °C. *ee* 38%, HPLC analysis: chiralpak OD-H (*i*-PrOH/hexane = 30:70, *v*/*v*, 1.0 mL/min, 254 nm) major *t*_R_ = 18.26 min and minor *t*_S_ = 24.19 min. ^1^H NMR (400 MHz, CDCl_3_) δ 8.07 (d, *J* = 6.4 Hz, 1H), 7.43 (d, *J* = 8.0 Hz, 1H), 7.35–7.26 (m, 2H), 7.24–7.03 (m, 8H), 7.02–6.89 (m, 3H), 4.91 (t, *J* = 7.7 Hz, 1H), 4.08 (dd, *J* = 16.5, 7.4 Hz, 1H), 3.95 (dd, *J* = 16.5, 8.1 Hz, 1H), 3.65 (s, 3H). ^13^C NMR (101 MHz, CDCl_3_) δ 197.1, 147.1, 144.1, 140.1, 137.2, 128.4, 127.9, 127.6, 127.1, 126.6, 126.4, 126.3, 125.4, 121.70, 119.6, 118.9, 117.4, 109.2, 77.4, 49.2, 38.6, 32.7.

(*R*)-2-(3-(1*H*-Indol-3-yl)-3-phenylpropanoyl)pyridine 1-oxide (**3ma**) [17]

Yellow crystals, 52 mg, yield 69%; m.p. 149.8–151.3 °C. *ee* 51%, HPLC analysis: chiralpak AD-H (*i*-PrOH/hexane = 30:70, *v*/*v*, 1.0 mL/min, 210 nm) major *t*_R_ = 10.59 min and minor *t*_S_ = 12.40 min, ^1^H NMR (400 MHz, CDCl_3_) δ 8.17 (d, *J* = 6.4 Hz, 1H), 8.00 (s, 1H), 7.35 (d, *J* = 8.0 Hz, 1H), 7.28–7.19 (m, 4H), 7.18–7.00 (m, 7H), 6.95–6.88 (m, 1H), 4.85 (t, *J* = 7.8 Hz, 1H), 4.02 (dd, *J* = 16.4, 7.7 Hz, 1H), 3.89 (dd, *J* = 16.4, 7.9 Hz, 1H). ^13^C NMR (101 MHz, CDCl_3_) δ 200.1, 153.3, 148.7, 144.5, 137.1, 136.6, 128.3, 128.0, 127.2, 126.8, 126.2, 122.1, 122.0, 121.5, 119.6, 119.5, 119.3, 111.0, 44.2, 38.2.

(*R*)-2-(3-(6-Methoxy-1*H*-indol-3-yl)-3-(4-methoxyphenyl)propanoyl)pyridine 1-oxide (**3ab**)

Brown crystals, 79 mg, yield 98%; m. p. 65.7–66.8 °C. *ee* 50%, HPLC analysis: chiralpak AS-H (*i*-PrOH/hexane = 30:70, *v*/*v*, 1.0 mL/min, 210 nm) major *t*_R_ = 18.01 min and minor *t*_R_ = 22.06 min. ^1^H NMR (400 MHz, CDCl_3_) δ 8.17 (d, *J* = 6.4 Hz, 1H), 8.10 (brs, 1H), 7.29–7.11 (m, 6H), 6.94 (s, 1H), 6.82–6.73 (m, 3H), 6.66 (dd, *J* = 8.7, 2.3 Hz, 1H), 4.84 (t, *J* = 7.8 Hz, 1H), 4.05 (dd, *J* = 16.3, 7.5 Hz, 1H), 3.98–3.87 (m, 1H), 3.79 (s, 3H), 3.75 (s, 3H). ^13^C NMR (101 MHz, CDCl_3_) δ 197.3, 158.0, 156.5, 147.2, 140.1, 137.3, 136.0, 128.8, 127.5, 126.5, 125.7, 121.1, 120.3, 120.1, 119.0, 113.7, 109.3, 94.6, 55.6, 55.2, 49.2, 37.9. HRMS (ESI): *m*/*z* Calcd for C_24_H_22_N_2_O_4_Na^+^ [M + Na]^+^: 425.1472, found: 425.1470.

(*R*)-2-(3-(4-Fluorophenyl)-3-(6-methoxy-1*H*-indol-3-yl)propanoyl)pyridine 1-oxide (**3ac**)

White crystals, 77 mg, yield 99%; m. p. 69.4–71.9 °C. *ee* 73%, HPLC analysis: chiralpak OD-H (*i*-PrOH/hexane = 30:70, *v*/*v*, 1.0 mL/min, 254 nm) major *t*_R_ = 18.26 min and minor *t*_S_ = 24.19 min. ^1^H NMR (400 MHz, CDCl_3_) δ 8.21 (d, *J* = 6.4 Hz, 1H), 8.07 (brs, 1H), 7.28–7.16 (s, 6H), 6.99 (s, 1H), 6.94–6.88 (m, 2H), 6.81 (d, *J* = 2.2 Hz, 1H), 6.67 (dd, *J* = 8.7, 2.3 Hz, 1H), 4.89 (t, *J* = 7.7 Hz, 1H), 4.05 (dd, *J* = 16.6, 7.5 Hz, 1H), 3.94 (dd, *J* = 16.6, 8.0 Hz, 1H), 3.80 (s, 3H). ^13^C NMR (101 MHz, CDCl_3_) δ 196.7, 162.6, 160.2, 156.5, 146.9, 140.2, 139.7, 137.3, 129.4, 129.3, 127.7, 126.6, 126.1, 121.0, 120.3, 119.9, 118.5, 115.2, 115.0, 109.4, 94.6, 55.6, 49.2, 37.8. ^19^F NMR (376 MHz, CDCl_3_) δ -116.86. HRMS (ESI): *m*/*z* Calcd for C_23_H_19_N_2_O_3_FNa^+^ [M + Na]^+^: 413.1272, found: 413.1281.

(*R*)-2-(3-(4-Chlorophenyl)-3-(6-methoxy-1*H*-indol-3-yl)propanoyl)pyridine 1-oxide (**3ad**)

Yellow crystals, 65 mg, yield 80%; m. p. 66.6–68.1 °C. *ee* 67%, HPLC analysis: chiralpak AS-H (*i*-PrOH/hexane = 30:70, *v*/*v*, 1.0 mL/min, 210 nm) major *t*_R_ = 21.68 min and minor *t*_R_ = 29.63 min. ^1^H NMR (400 MHz, CDCl_3_) δ 8.17 (d, *J* = 6.5 Hz, 1H), 8.04 (brs, 1H), 7.34–7.15 (m, 8H), 6.99 (dd, *J* = 2.3, 1.0 Hz, 1H), 6.81 (d, *J* = 2.2 Hz, 1H), 6.68 (dd, *J* = 8.7, 2.2 Hz, 1H), 4.90 (t, *J* = 7.7 Hz, 1H), 4.06 (dd, *J* = 16.7, 7.4 Hz, 1H), 3.95 (dd, *J* = 16.7, 7.9 Hz, 1H), 3.81 (s, 3H). ^13^C NMR (101 MHz, CDCl_3_) δ 196.6, 156.6, 146.9, 142.6, 140.3, 137.3, 132.0, 129.3, 128.5, 127.7, 126.8, 125.6, 120.9, 120.4, 119.9, 118.3, 109.4, 94.6, 55.6, 49.0, 37.9. HRMS (ESI): *m*/*z* Calcd for C_23_H_19_N_2_O_3_ClNa^+^ [M + Na]^+^: 429.0976, found: 429.1003.

(*R*)-2-(3-(4-Bromophenyl)-3-(6-methoxy-1*H*-indol-3-yl)propanoyl)pyridine 1-oxide (**3ae**)

Yellow crystals, 68 mg, yield 75%; m. p. 163.7–165.9 °C. *ee* 63%, HPLC analysis: chiralpak AS-H (*i*-PrOH/hexane = 40:60, *v*/*v*, 1.0 mL/min, 254 nm) major *t*_R_ = 14.18 min and minor *t*_S_ = 17.78 min.^1^H NMR (400 MHz, CDCl_3_) δ 8.26 (brs, 1H), 8.17 (d, *J* = 6.5 Hz, 1H), 7.36–7.31 (m, 2H), 7.28 (s, 3H), 7.21–7.11 (m, 3H), 6.97–6.92 (m, 1H), 6.78 (d, *J* = 2.2 Hz, 1H), 6.67 (dd, *J* = 8.7, 2.2 Hz, 1H), 4.87 (t, *J* = 7.6 Hz, 1H), 4.05 (dd, *J* = 16.8, 7.4 Hz, 1H), 3.95 (dd, *J* = 16.7, 8.0 Hz, 1H), 3.78 (s, 3H). ^13^C NMR (101 MHz, CDCl_3_) δ 196.4, 156.6, 146.8, 143.1, 140.3, 137.3, 131.4, 129.7, 127.8, 126.76, 125.9, 120.9, 120.4, 120.1, 119.9, 118.2, 109.4, 94.6, 55.6, 48.9, 37.9. HRMS (ESI): *m*/*z* Calcd for C_23_H_19_N_2_O_3_BrNa^+^ [M + Na]^+^: 473.0471, found: 473.0479.

(*R*)-2-(3-(4-Cyanophenyl)-3-(6-methoxy-1*H*-indol-3-yl)propanoyl)pyridine 1-oxide (**3af**)

Yellow crystals, 69 mg, yield 87%; m. p. 85.9–87.5 °C. *ee* 71%, HPLC analysis: chiralpak AD-H (*i*-PrOH/hexane = 30:70, *v*/*v*, 1.0 mL/min, 254 nm) major *t*_R_ = 38.39 min and minor *t*_S_ = 47.41 min. ^1^H NMR (400 MHz, CDCl_3_) δ 8.38 (brs, 1H), 8.18 (d, *J* = 6.4 Hz, 1H), 7.60–7.42 (m, 4H), 7.38–7.30 (m, 2H), 7.19 (t, *J* = 8.2 Hz, 2H), 7.06–6.96 (m, 1H), 6.79 (d, *J* = 2.0 Hz, 1H), 6.67 (dd, *J* = 8.7, 2.2 Hz, 1H), 4.99 (t, *J* = 7.5 Hz, 1H), 4.17–3.94 (m, 2H), 3.82–3.68 (m, 3H). ^13^C NMR (101 MHz, CDCl_3_) δ 195.6, 156.7, 149.8, 146.5, 140.4, 137.3, 132.3, 128.8, 128.1, 126.9, 126.3, 120.7, 120.6, 119.6, 119.0, 117.3, 110.1, 109.6, 94.7, 55.6, 48.8, 38.3. HRMS (ESI): *m*/*z* Calcd for C_24_H_19_N_3_O_3_Na^+^ [M + Na]^+^: 420.1319, found: 420.1330.

(*R*)-2-(3-(6-Methoxy-1*H*-indol-3-yl)-3-(4-(trifluoromethyl)phenyl)propanoyl)pyridine 1-oxide (**3ag**)

Yellow crystals, 76 mg, yield 86%; m. p. 73.2–74.5°C. *ee* 77%, HPLC analysis: chiralpak AD-H (*i*-PrOH/hexane = 40:60, *v*/*v*, 1.0 mL/min, 254 nm) major *t*_R_ = 15.65 min and minor *t*_S_ = 21.80 min. ^1^H NMR (400 MHz, CDCl_3_) δ 8.18 (dt, *J* = 6.2, 1.0 Hz, 1H), 8.09 (brs, 1H), 7.56–7.43 (m, 4H), 7.39–7.13 (m, 4H), 7.02 (dq, *J* = 3.2, 1.6 Hz, 1H), 6.81 (d, *J* = 2.2 Hz, 1H), 6.69 (dd, *J* = 8.7, 2.3 Hz, 1H), 5.00 (t, *J* = 7.6 Hz, 1H), 4.22–3.92 (m, 2H), 3.81 (d, *J* = 1.0 Hz, 3H). ^13^C NMR (101 MHz, CDCl_3_) δ 196.2, 156.7, 148.2, 146.7, 140.3, 137.3, 128.3, 127.8, 126.8, 125.6, 125.3, 120.9, 120.5, 119.8, 117.9, 109.5, 94.7, 55.6, 48.9, 38.2. ^19^F NMR (376 MHz, CDCl_3_) δ −62.34. HRMS (ESI): *m*/*z* Calcd for C_24_H_19_N_2_O_3_F_3_Na^+^ [M + Na]^+^: 463.1240, found: 463.1252.

(*S*)-2-(3-(6-Methoxy-1*H*-indol-3-yl)-3-(2-methoxyphenyl)propanoyl)pyridine 1-oxide (**3ah**)

Yellow crystals, 60 mg, yield 74%; m. p. 88.4–90.5 °C. *ee* 38%, HPLC analysis: chiralpak AS-H (*i*-PrOH/hexane = 40:0, *v*/*v*, 1.0 mL/min, 214 nm) major *t*_R_ = 18.51 min and minor *t*_S_ = 23.56 min. ^1^H NMR (400 MHz, CDCl_3_) δ 8.13 (d, *J* = 6.5 Hz, 1H), 7.94 (brs, 1H), 7.36 (d, *J* = 8.7 Hz, 1H), 7.23 (ddd, *J* = 6.6, 5.5, 4.2 Hz, 1H), 7.16–7.05 (m, 4H), 6.95 (dd, *J* = 2.4, 1.2 Hz, 1H), 6.83–6.72 (m, 3H), 6.66 (dd, *J* = 8.7, 2.3 Hz, 1H), 5.31 (t, *J* = 7.8 Hz, 1H), 4.14 (dd, *J* = 16.4, 8.3 Hz, 1H), 3.78 (d, *J* = 1.5 Hz, 7H). ^13^C NMR (101 MHz, CDCl_3_) δ 197.5, 156.6, 156.4, 147.4, 140.0, 137.2, 131.9, 128.6, 127.4, 127.2, 126.4, 125.3, 121.5, 120.7, 120.6, 120.2, 118.6, 110.4, 109.2, 94.5, 55.6, 55.4, 47.7, 31.3. HRMS (ESI): *m*/*z* Calcd for C_24_H_22_N_2_O_4_Na^+^ [M + Na]^+^: 425.1472, found: 425.1475.

(*S*)-2-(3-(2-Fluorophenyl)-3-(6-methoxy-1*H*-indol-3-yl)propanoyl)pyridine 1-oxide (**3ai**)

Brown crystals, 60 mg, yield 77%; m. p. 60.8–63.1 °C. *ee* 53%, HPLC analysis: chiralpak AD-H (*i*-PrOH/hexane = 30:70, *v*/*v*, 1.0 mL/min, 254 nm) major *t*_R_ = 23.97 min and minor *t*_S_ = 38.42 min. ^1^H NMR (400 MHz, CDCl_3_) δ 8.18 (d, *J* = 6.4 Hz, 1H), 8.03 (brs, 1H), 7.38 (d, *J* = 8.7 Hz, 1H), 7.34–7.22 (m, 3H), 7.22–7.08 (m, 2H), 7.06–6.95 (m, 3H), 6.80 (d, *J* = 2.2 Hz, 1H), 6.71 (dd, *J* = 8.7, 2.2 Hz, 1H), 5.25 (t, *J* = 7.7 Hz, 1H), 4.22 (dd, *J* = 17.0, 7.6 Hz, 1H), 3.93 (dd, *J* = 17.0, 7.7 Hz, 1H), 3.81 (s, 3H). ^13^C NMR (101 MHz, CDCl_3_) δ 196.4, 159.3, 156.6, 146.9, 140.3, 137.2, 130.7, 129.4, 128.0, 127.9, 127.7, 126.7, 125.6, 124.1, 121.1, 120.6, 119.8, 117.7, 115.5, 115.3, 109.4, 94.6, 55.6, 47.7, 31.1. ^19^F NMR (376 MHz, CDCl_3_) δ −117.82. HRMS (ESI): *m*/*z* Calcd for C_23_H_19_N_2_O_3_FNa^+^ [M + Na]^+^: 413.1272, found: 413.1273.

(*S*)-2-(3-(2-Chlorophenyl)-3-(6-methoxy-1*H*-indol-3-yl)propanoyl)pyridine 1-oxide (**3aj**)

Colorless crystals, 72 mg, yield 89%; m. p. 162.6–164.5 °C. *ee* 44%, HPLC analysis: chiralpak AD-H (*i*-PrOH/hexane = 40:60, *v*/*v*, 1.0 mL/min, 254 nm) major *t*_R_ = 14.79 min and minor *t*_S_ = 19.25 min. ^1^H NMR (400 MHz, CDCl_3_) δ 8.16 (d, *J* = 6.4 Hz, 1H), 7.92 (brs, 1H), 7.36–7.17(s, 5H), 7.18 (d, *J* = 7.6 Hz, 1H), 7.12–6.99 (m, 3H), 6.79 (d, *J* = 2.2 Hz, 1H), 6.68 (dd, *J* = 8.7, 2.3 Hz, 1H), 5.42 (t, *J* = 7.6 Hz, 1H), 4.22 (dd, *J* = 17.0, 8.3 Hz, 1H), 3.79 (s, 4H). ^13^C NMR (101 MHz, CDCl_3_) δ 196.3, 156.6, 146.9, 141.1, 140.3, 137.3, 133.5, 129.5, 129.3, 127.7, 127.0, 126.7, 125.6, 121.2, 120.9, 120.1, 117.8, 109.5, 94.6, 55.6, 47.7, 34.6. HRMS (ESI): *m*/*z* Calcd for C_23_H_19_N_2_O_3_ClNa^+^ [M + Na]^+^: 429.0976, found: 429.0992.

(*S*)-2-(3-(2-Bromophenyl)-3-(6-methoxy-1H-indol-3-yl)propanoyl)pyridine 1-oxide (**3ak**)

Yellow crystals, 86 mg, yield 95%; m. p. 108.7–110.3 °C. *ee* 41%, HPLC analysis: chiralpak AD-H (*i*-PrOH/hexane = 40:60, *v*/*v*, 1.0 mL/min, 254 nm) major *t*_R_ = 13.40 min and minor *t*_S_ = 17.21 min. ^1^H NMR (400 MHz, CDCl_3_) δ 8.17 (d, *J* = 5.9 Hz, 2H), 7.54 (dd, *J* = 8.0, 1.3 Hz, 1H), 7.36 (d, *J* = 8.7 Hz, 1H), 7.31–7.08 (m, 5H), 7.01 (td, *J* = 6.8, 2.6 Hz, 2H), 6.79 (d, *J* = 2.3 Hz, 1H), 6.70 (dd, *J* = 8.7, 2.3 Hz, 1H), 5.41 (t, *J* = 7.6 Hz, 1H), 4.23 (dd, *J* = 16.9, 8.5 Hz, 1H), 3.79 (s, 4H). ^13^C NMR (101 MHz, DMSO-*d*_6_) δ 197.8, 152.3, 146.9, 145.0, 140.5, 138.3, 132.2, 128.8, 128.8, 128.7, 128.1, 128.1, 127.1, 126.4, 126.3, 126.2, 123.3, 117.6, 112.5, 112.1, 102.8, 70.3, 48.4, 38.2. HRMS (ESI): *m*/*z* Calcd for C_23_H_19_N_2_O_3_BrNa^+^ [M + Na]^+^: 473.0471, found: 473.0470.

(*R*)-2-(3-(6-Methoxy-1*H*-indol-3-yl)-3-(naphthalen-2-yl)propanoyl)pyridine 1-oxide (**3al**)

Yellow crystals, 81 mg, yield 96%; m. p. 80.9–82.6 °C. *ee* 60%, HPLC analysis: chiralpak AD-H (*i*-PrOH/hexane = 40:60, *v*/*v*, 1.0 mL/min, 254 nm) major *t*_R_ = 20.71 min and minor *t*_S_ = 23.55 min. ^1^H NMR (400 MHz, CDCl_3_) δ 8.12 (d, *J* = 6.5 Hz, 1H), 8.03 (brs, 1H), 7.78–7.69 (m, 3H), 7.66 (d, *J* = 8.5 Hz, 1H), 7.44–7.35 (m, 3H), 7.28 (d, *J* = 8.7 Hz, 1H), 7.19 (td, *J* = 7.1, 2.6 Hz, 1H), 7.08 (dt, *J* = 7.9, 2.2 Hz, 1H), 6.99 (tt, *J* = 5.3, 2.6 Hz, 2H), 6.76 (d, *J* = 2.2 Hz, 1H), 6.61 (dd, *J* = 8.7, 2.3 Hz, 1H), 5.05 (t, *J* = 7.7 Hz, 1H), 4.14 (dd, *J* = 16.5, 7.4 Hz, 1H), 4.05 (dd, *J* = 16.5, 8.0 Hz, 1H), 3.75 (d, *J* = 1.5 Hz, 3H). ^13^C NMR (101 MHz, CDCl_3_) δ 197.1, 156.5, 147.1, 141.4, 140.1, 137.3, 133.4, 132.3, 128.1, 127.8, 127.5, 126.6, 126.5, 126.1, 125.9, 125.5, 125.4, 121.2, 120.5, 120.1, 118.6, 109.4, 94.6, 55.6, 48.9, 38.7. HRMS (ESI): *m*/*z* Calcd for C_27_H_22_N_2_O_3_Na^+^ [M + Na]^+^: 445.1523, found: 445.1530.

(*S*)-2-(3-(Furan-2-yl)-3-(6-methoxy-1*H*-indol-3-yl)propanoyl)pyridine 1-oxide (**3am**)

Yellow crystals, 61 mg, yield 84%; m. p. 78.9–80.6 °C. *ee* 41%, HPLC analysis: chiralpak AS-H (*i*-PrOH/hexane = 30:70, *v*/*v*, 1.0 mL/min, 214 nm) major *t*_R_ = 29.72 min and minor *t*_S_ = 32.99 min. ^1^H NMR (400 MHz, CDCl_3_) δ 8.14 (d, *J* = 6.0 Hz, 1H), 7.91 (brs, 1H), 7.43 (d, *J* = 8.6 Hz, 1H), 7.29–7.23 (m, 4H), 7.15 (td, *J* = 7.4, 1.2 Hz, 1H), 6.97 (d, *J* = 2.4 Hz, 1H), 6.79 (d, *J* = 2.3 Hz, 1H), 6.72 (dd, *J* = 8.6, 2.2 Hz, 1H), 6.23 (dd, *J* = 3.2, 1.8 Hz, 1H), 6.04 (d, *J* = 3.1 Hz, 1H), 4.99 (t, *J* = 7.6 Hz, 1H), 4.00 (d, *J* = 7.7 Hz, 2H), 3.81 (s, 3H). ^13^C NMR (101 MHz, CDCl_3_) δ 196.5, 156.8, 156.5, 146.92, 141.2, 140.2, 137.1, 127.6, 126.6, 125.4, 120.8, 120.0, 116.3, 110.1, 109.5, 105.6, 94.6, 55.7, 47.2, 32.4. HRMS (ESI): *m*/*z* Calcd for C_21_H_18_N_2_NaO_4_^+^ [M + Na]^+^: 385.1159, found: 385.1124.

## 4. Conclusions

In summary, we prepared a series of enantiopure enlarged Salen ligands containing flexible and rigid combined linkers. The enlarged Salen ligands are more suitable for coordination with large rare earth elements. At the same time, the electronic effect of the coordinating sites of the ligands is tuned by substituents of the salicylaldehyde moiety. The results indicate that the enlarged Salen ligand linked with a flexible and rigid combined linker of trimethylene-connected diamide and a strong electron-withdrawing trifluoromethyl substituent group can achieve high reactivity and excellent stereoselectivity in the catalytic asymmetric Michael addition reaction of indoles and 2-cinnamoylpyridine 1-oxides, which provides an efficient way for the optically active modification of the 3-position of indoles in the future.

## Figures and Tables

**Table 1:** Screening of ligands for the asymmetric Michael addition of indole and enone ^a^.

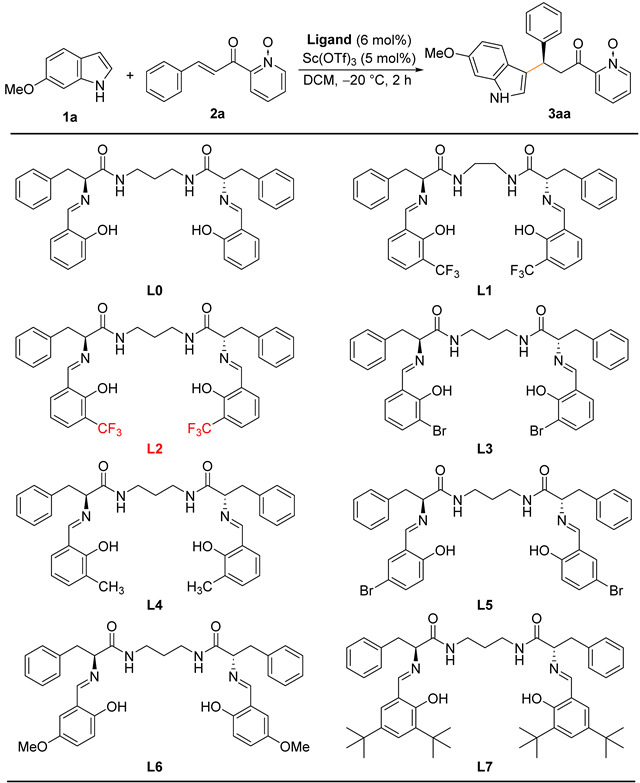			
Entry	Ligand	Yield ^b^/%	*ee* ^c^/%
1	**L0**	81	21
2	**L1**	90	35
3	**L2**	65	96
4	**L3**	95	60
5	**L4**	88	18
6	**L5**	77	6
7	**L6**	91	12
8	**L7**	98	11

^a^ **2a** (0.2 mmol), ligand **L** (6 mol%), and Sc(OTf)_3_ (5 mol%) in dried CH_2_Cl_2_ (2 mL) were stirred under N_2_ atmosphere at R.T. for 1 h. **1a** (0.24 mmol) was added, and the resulting mixture was stirred at −20 °C for 2 h. ^b^ Isolated yield. ^c^ Determined by HPLC using a Chiralpak AD-H column.

**Table 2:** Optimization of reaction conditions for asymmetric Michael addition of indole and enone ^a^.

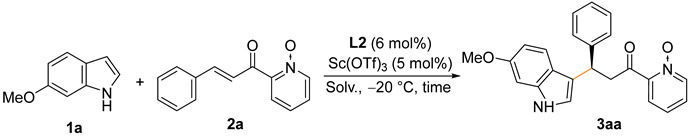				
Entry	Solvent	Time/h	Yield ^c^/%	*ee* ^d^/%
1	CH_2_Cl_2_	2	65	96
2	CHCl_3_	2	79	88
3	DCE	4	42	14
4	CH_3_CN	3	61	58
5	Toluene	3	37	66
6	DME	4	62	19
7	MTBE	4	34	24
8	THF	4	62	75
9	EA	4	89	14
10 ^b^	CH_2_Cl_2_	2	42	0

^a^ **2a** (0.2 mmol), ligand **L2** (6 mol%), and Sc(OTf)_3_ (5 mol%) in dried solvent (2 mL) were stirred under N_2_ atmosphere at R.T. for 1 h. **1a** (0.24 mmol) was added and the resulting mixture was stirred at −20 °C for the indicated time. ^b^ Commercially available solvent. ^c^ Isolated yield. ^d^ Determined by HPLC using a Chiralpak AD-H column.

**Table 3:** Catalytic asymmetric Michael addition of indole derivatives and enone **2a** ^a^.

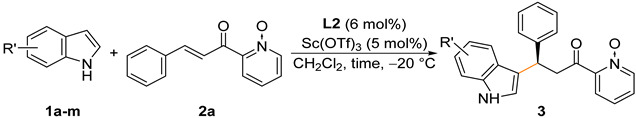					
Entry	Indole	Product 3	Time/h	Yield ^b^/%	*ee* ^c^/%
1	**1a**, R′: 6-MeO	**3aa**	2	65	96
2	**1b**, R′: 6-Me	**3ba**	1	74	94
3	**1c**, R′: 6-Br	**3ca**	3	60	98
4	**1d**, R′: 6-F	**3da**	4	86	99
5	**1e**, R′: 6-CO_2_Me	**3ea**	18	38	23
6	**1f**, R′: 5-MeO	**3fa**	2	67	88
7	**1g**, R′: 5-BnO	**3ga**	4	98	78
8	**1h**, R′: 5-Cl	**3ha**	3	69	98
9	**1i**, R′: 5-F	**3ia**	5	84	93
10	**1j**, R′: 4-MeO	**3ja**	6	79	57
11	**1k**, R′: 1-Me	**3ka**	1	65	38
12	**1l**, R′: 1-PhSO_2_	**3la**	4	N.R.	/
13	**1m**, R′: H	**3ma**	2	69	51

^a^ All reactions were carried out with **2a** (0.2 mmol) and ligand **L2** (6 mol%)–Sc(OTf)_3_ complex (5 mol%) in dry CH_2_Cl_2_ (2 mL) under N_2_ atmosphere at R.T. for 1 h. Then, **1** (0.24 mmol) was added, and the resulting mixture was stirred at −20 °C. ^b^ Isolated yield. ^c^ Determined by HPLC analysis.

**Table 4:** Catalytic asymmetric Michael addition of 6-methoxyindole (**1a**) with various 2-enoylpyridine *N*-oxides **2** ^a^.

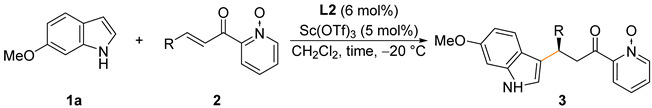					
Entry	Product 3	R	Time/h	Yield ^b^/%	*ee* ^c^/%
1	**3ab**	4-MeOC_6_H_4_	2	98	50
2	**3ac**	4-FC_6_H_4_	2	99	73
3	**3ad**	4-ClC_6_H_4_	2	80	67
4	**3ae**	4-BrC_6_H_4_	2	75	63
5	**3af**	4-NCC_6_H_4_	2	87	71
6	**3ag**	4-CF_3_C_6_H_4_	4	86	77
7	**3ah**	2-MeOC_6_H_4_	2	74	38
8	**3ai**	2-FC_6_H_4_	1	77	53
9	**3aj**	2-ClC_6_H_4_	2	89	44
10	**3ak**	2-BrC_6_H_4_	2	95	41
11	**3al**	2-Naphthyl	2	96	60
12	**3am**	2-Furyl	2	84	41

^a^ All the reactions were carried out with **2** (0.2 mmol) and ligand **L2** (6 mol%)–Sc(OTf)_3_ complex (5 mol%) in dry CH_2_Cl_2_ (2 mL) with under N_2_ atmosphere at R.T. for 1 h. Then, **1a** (0.24 mmol) as added, and the resulting mixture was stirred at −20 °C. ^b^ Isolated yield. ^c^ Determined by HPLC analysis.

**Table 5:** Stereoselectivities of the model reaction under the catalysis of different enantiomeric excesses of ligand **L2**.

**L2** ee (%)	0.5	12	19	30	64	82	90	99
**3aa** ee (%)	1	5	10	19	19	39	54	94

## Data Availability

Data are contained within the article or Supplementary Materials.

## Supplementary Materials

The following supporting information can be downloaded at: www.mdpi.com/article/10.3390/molecules30030459/s1. Copies of ^1^H-NMR, ^13^C-NMR, and ^19^F-NMR spectra and HPLC profiles of compounds **3** and **L**, and HRMS spectra of unknown products **L** and **3** are included in the Supporting Information.
