# Supplementary material for: Scandium(III)-Enlarged Salen Complex-Catalyzed Asymmetric Michael Addition of Indoles to Enones
Source: Molecules. 2025 Jan 21;30(3):459. doi: 10.3390/molecules30030459 (PMC11820277; doi:10.3390/molecules30030459)
Supplement: Supplementary file 1 [file molecules-30-00459-s001.zip › molecules-3425955-supplementary.pdf]

Supporting Information

Scandium(III)-Enlarged Salen Complex-Catalyzed Asymmetric Michael Addition of Indoles to Enones

Ningning Li, Quanyu Ma, and Jiaxi Xu*

State Key Laboratory of Chemical Resource Engineering, Department of Organic Chemistry, College of Chemistry, Beijing University of Chemical Technology, Beijing 100029, China

* Correspondence: jxxu@mail.buct.edu.cn; Tel.: +86-10-6443-5565; fax: +8610-6443-5565

Contents

1. **Copies of NMR spectra of Ligands L ……………………………….………………S2**
2. **Copies of NMR spectra of products 3………………………………….…………S12**
3. **Copies of HPLC profiles of products 3…………………………..………………..S42**

1. Copies of NMR spectra of Ligands

(2*S*,2'*S*)-*N*,*N'*-(Propane-1,3-diyl)bis(2-(((*E*)-2-hydroxybenzylidene)amino)-3-phenylpropanamide) (**L0**)

^{1}H NMR (400 MHz, $CDCl_3$)

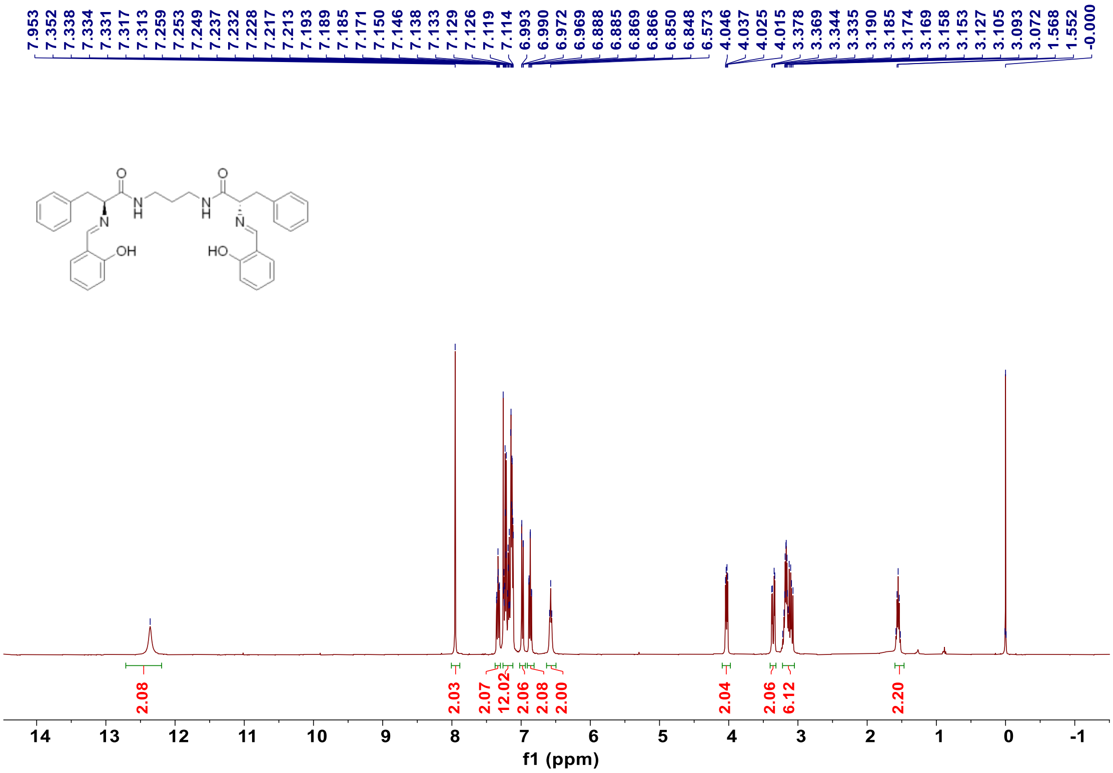

^{13}C{^{1}H} NMR (101 MHz, $CDCl_3$)

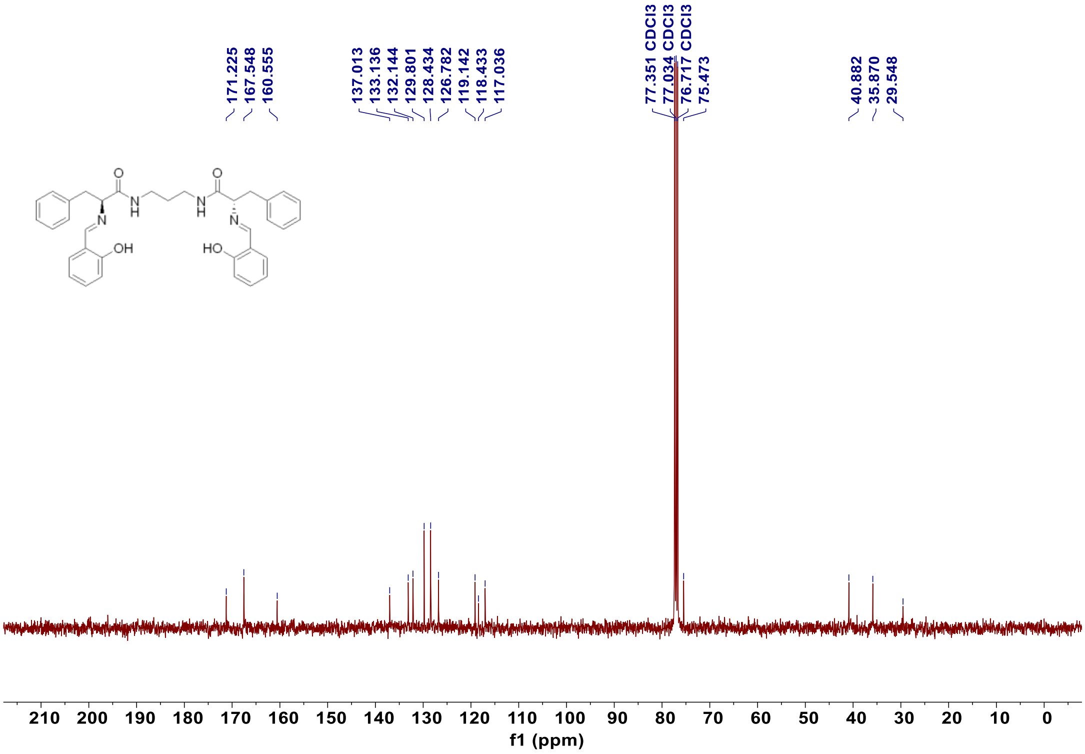

(2*S*,2'*S*)-*N*,*N*'-(Ethane-1,2-diyl)bis(2-(((*E*)-2-hydroxy-3-(trifluoromethyl)benzylidene) amino)-3-phenylpropanamide) (**L1**)

^{1}H NMR (400 MHz, $CDCl_3$)

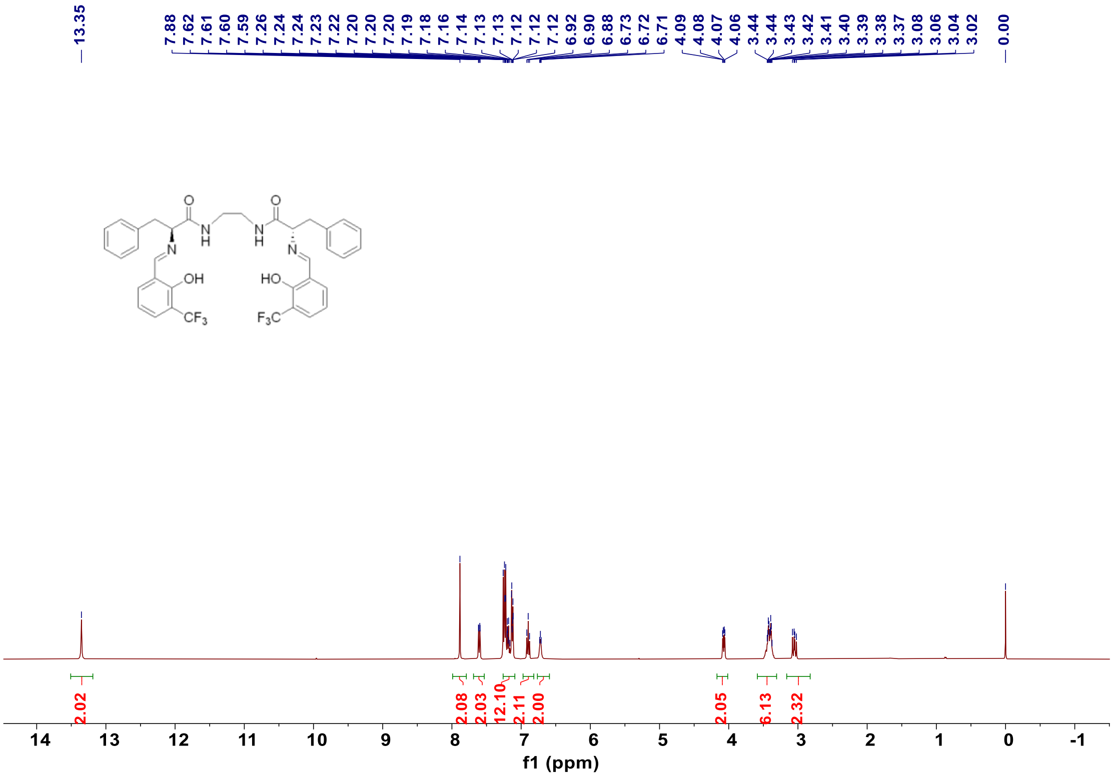

^{13}C{^{1}H} NMR (101 MHz, $CDCl_3$)

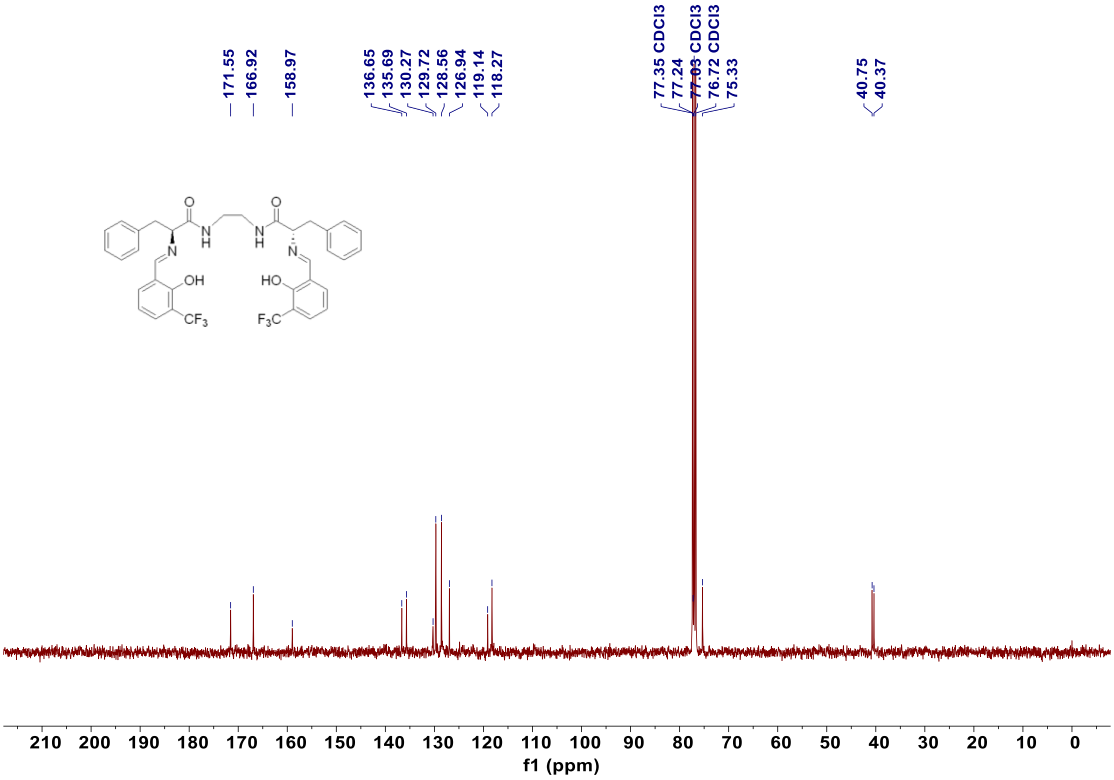

^{19}F NMR (377 MHz, $CDCl_3$)

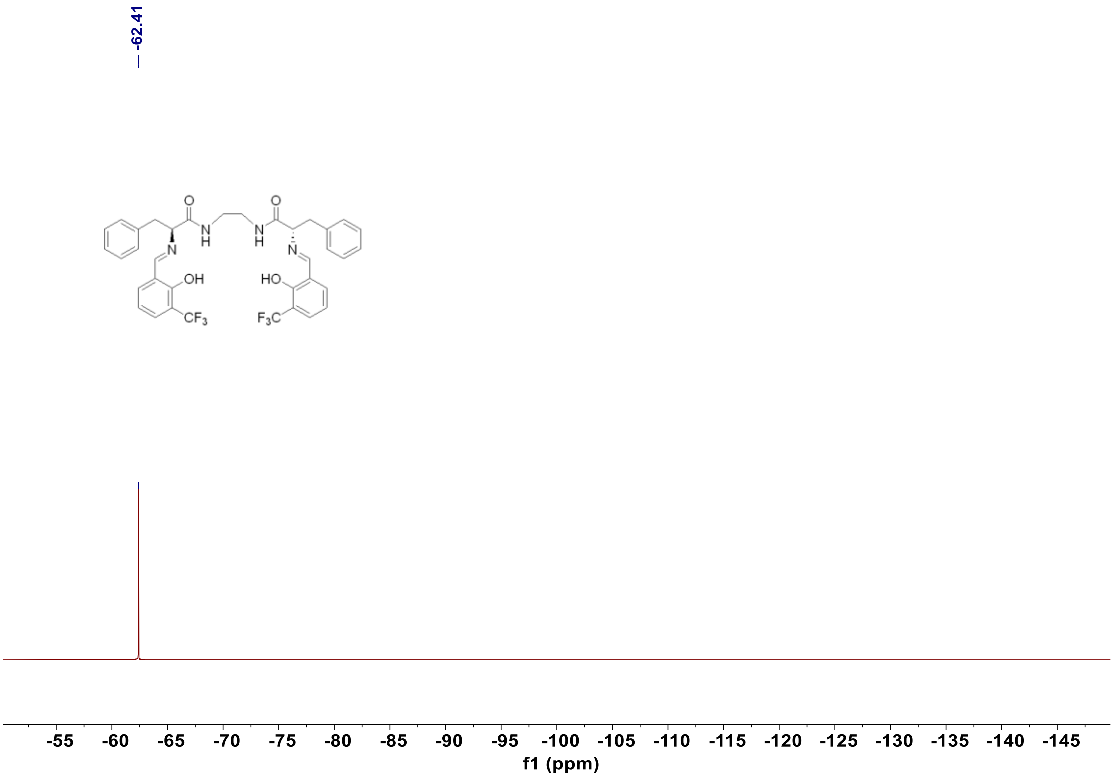

(2*S*,2'*S*)-*N*,*N*'-(Propane-1,3-diyl)bis(2-(((*E*)-2-hydroxy-3-(trifluoromethyl)benzylidene) amino)-3-phenylpropanamide) (**L2**)

^{1}H NMR (400 MHz, $CDCl_3$)

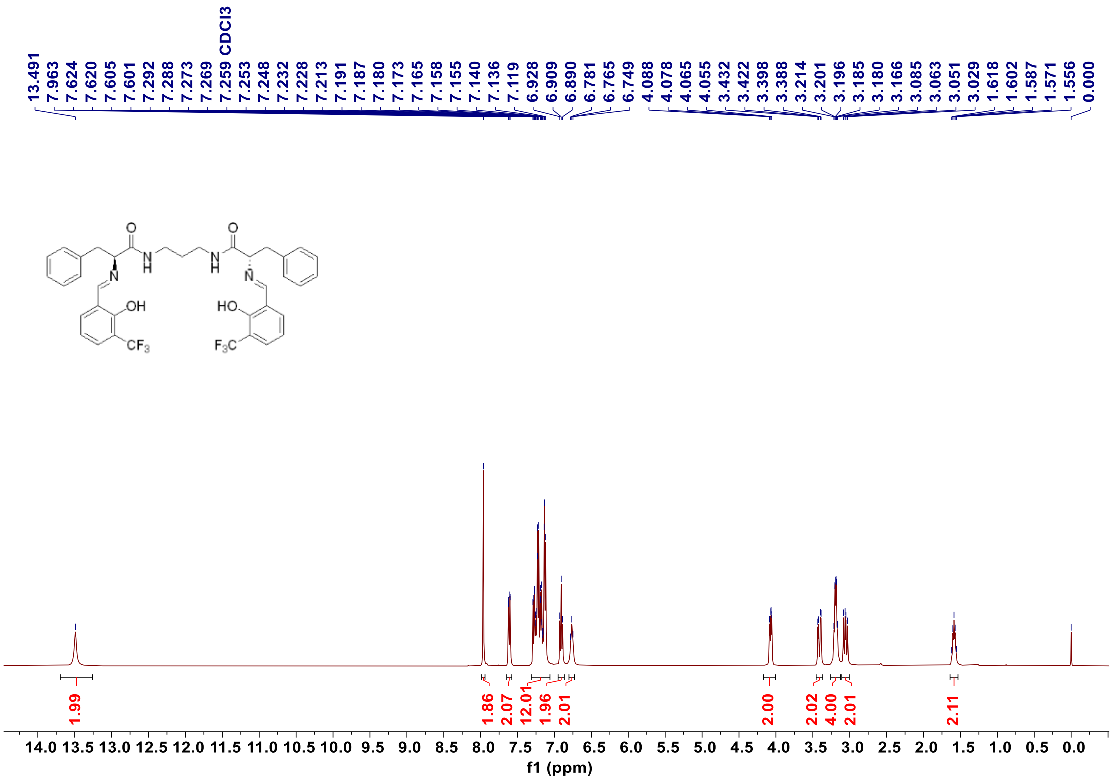

$^{13}C\{^1H\}$ NMR (101 MHz, $CDCl_3$)

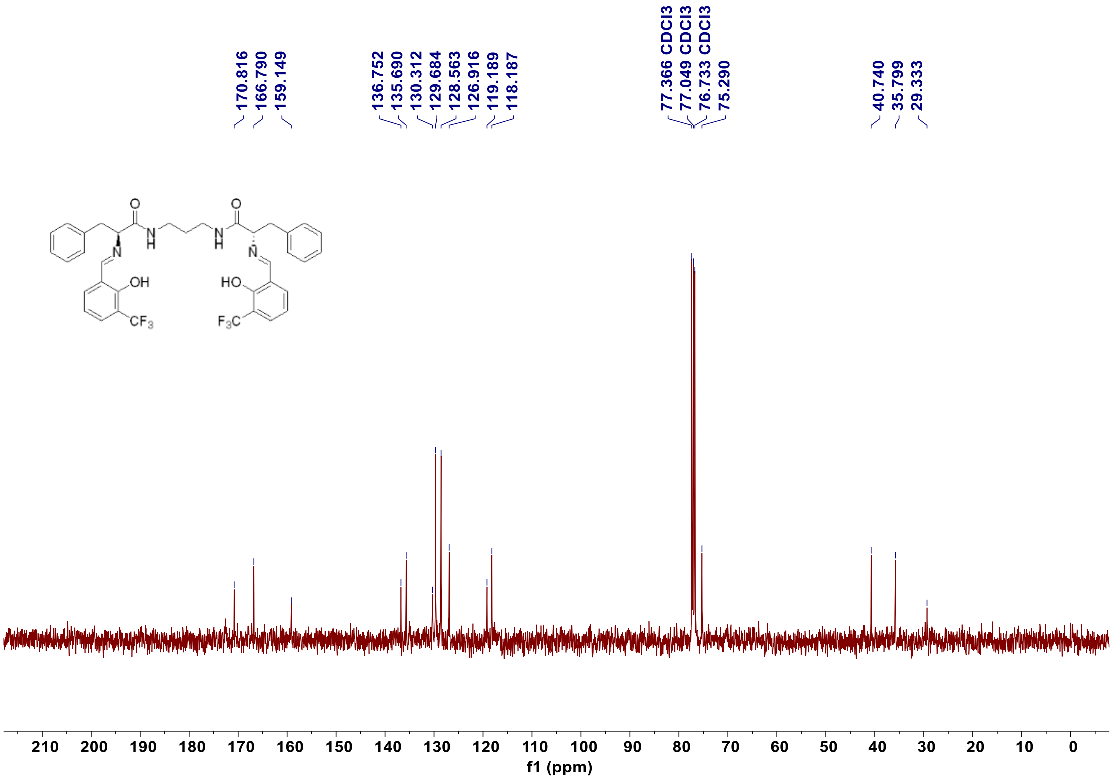

^{19}F NMR (377 MHz, $CDCl_3$)

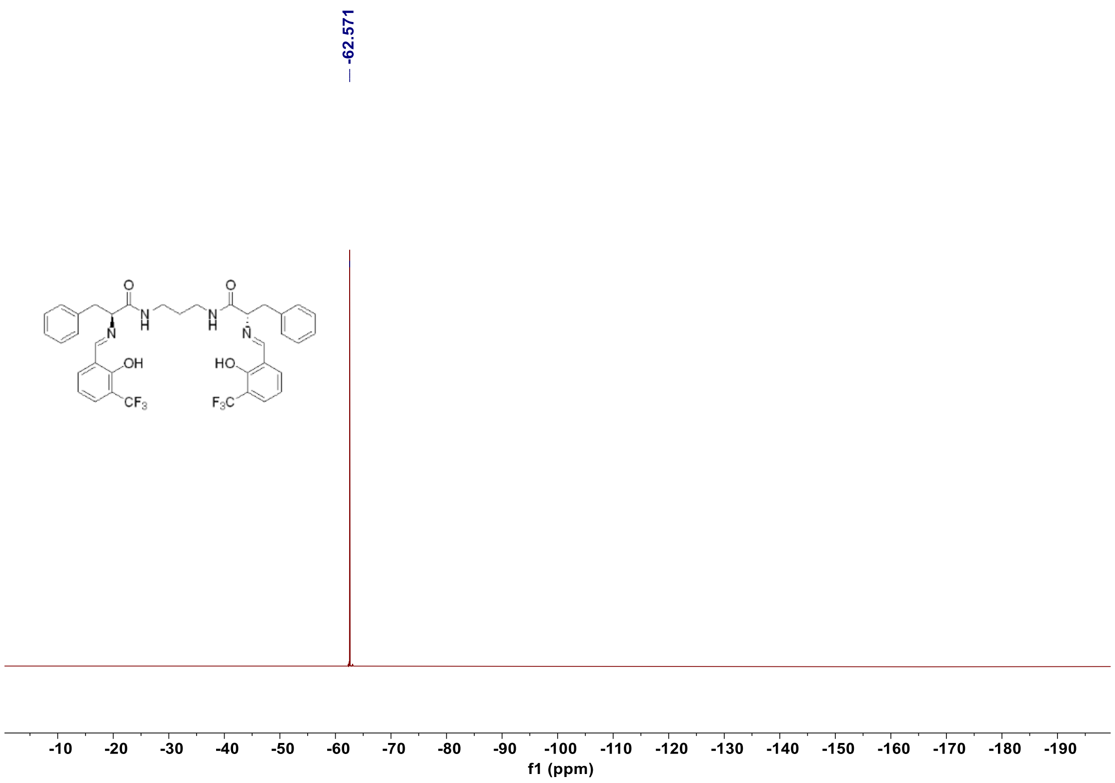

(2*S*,2'*S*)-*N*,*N*'-(Propane-1,3-diyl)bis(2-(((*E*)-3-bromo-2-hydroxybenzylidene)amino)-3-phenylpropanamide) (**L3**)

^{1}H NMR (400 MHz, $CDCl_3$)

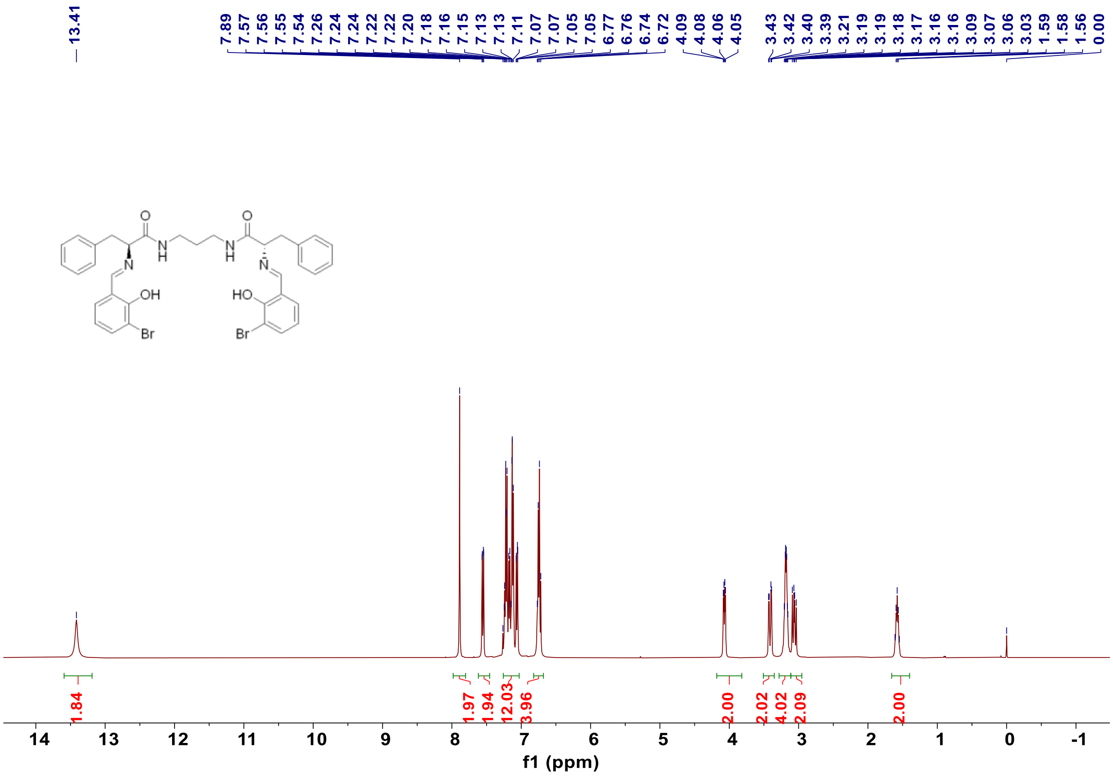

$^{13}C\{^{1}H\}$ NMR (101 MHz, $CDCl_3$)

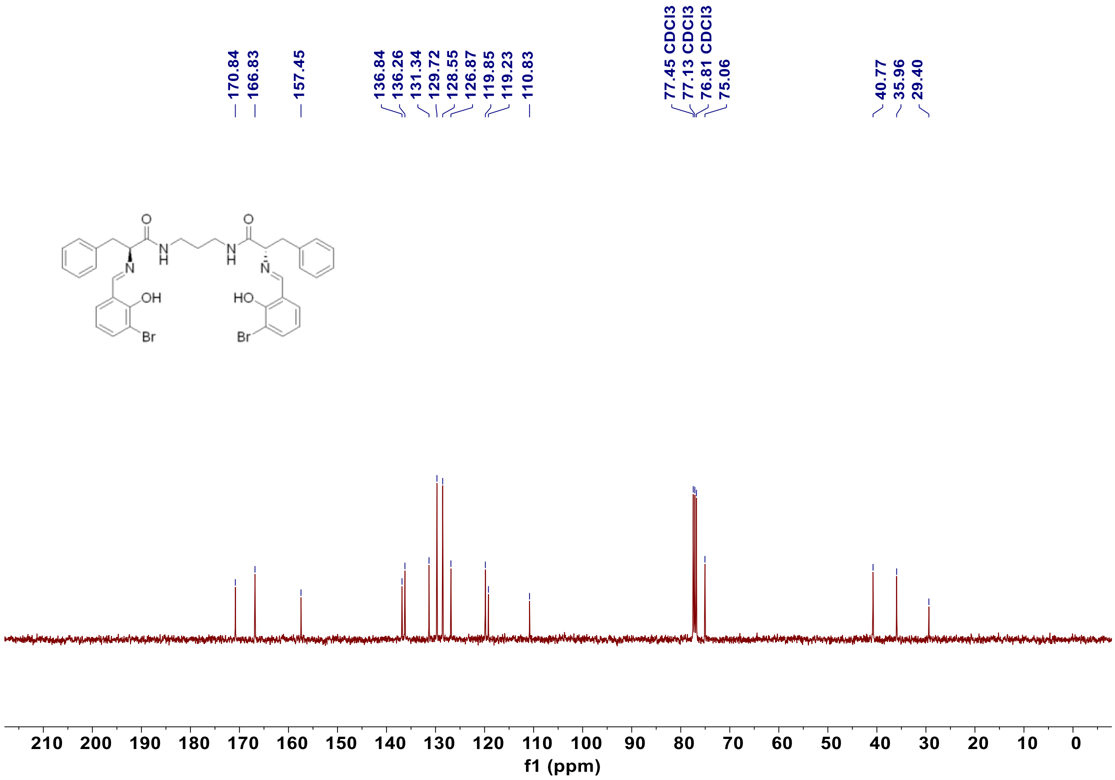

(2*S*,2'*S*)-*N*,*N*'-(Propane-1,3-diyl)bis(2-(((*E*)-2-hydroxy-3-methylbenzylidene)amino)-3-phenylpropanamide) (**L4**)

^{1}H NMR (400 MHz, $CDCl_3$)

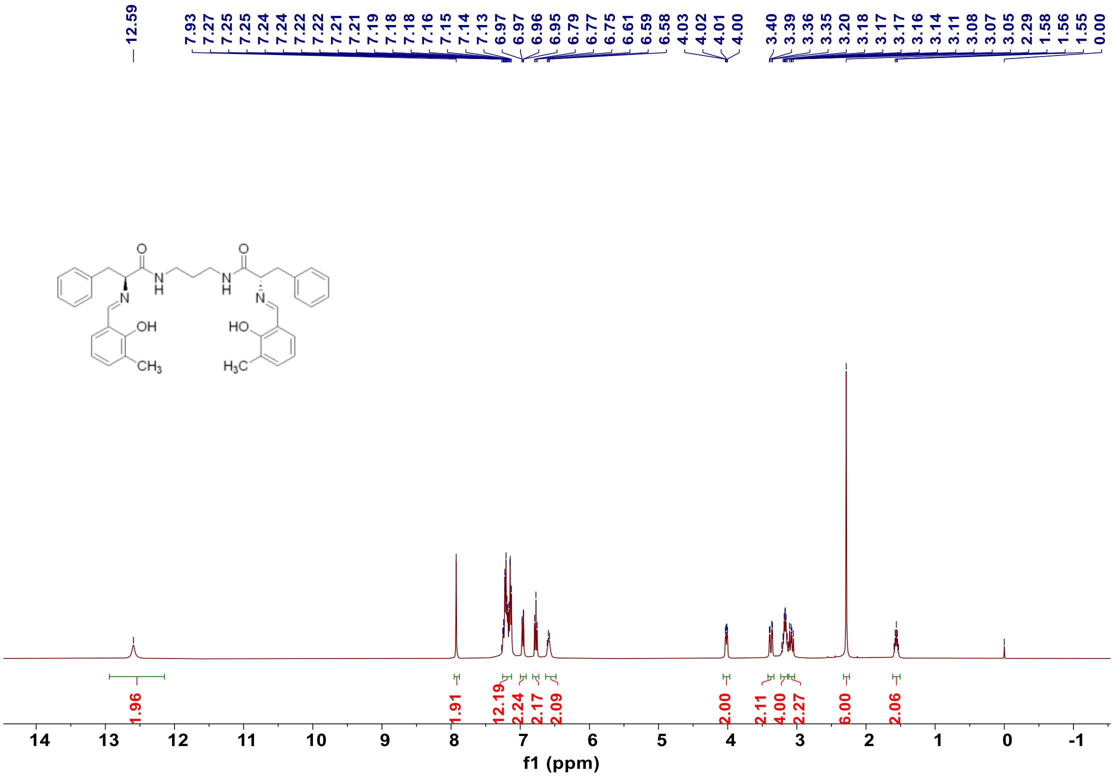

^{13}C{^{1}H} NMR (101 MHz, $CDCl_3$)

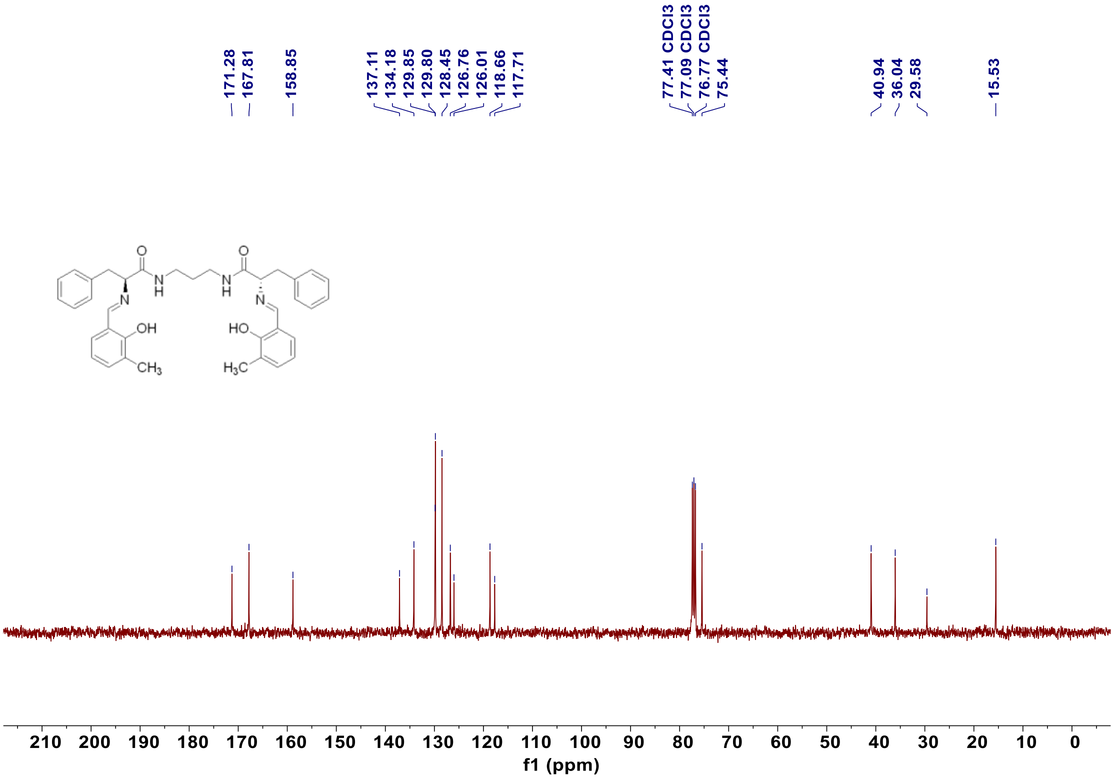

(2*S*,2'*S*)-*N*,*N*'-(Propane-1,3-diyl)bis(2-(((*E*)-5-bromo-2-hydroxybenzylidene)amino)-3-phenylpropanamide) (**L5**)

^{1}H NMR (400 MHz, $CDCl_3$)

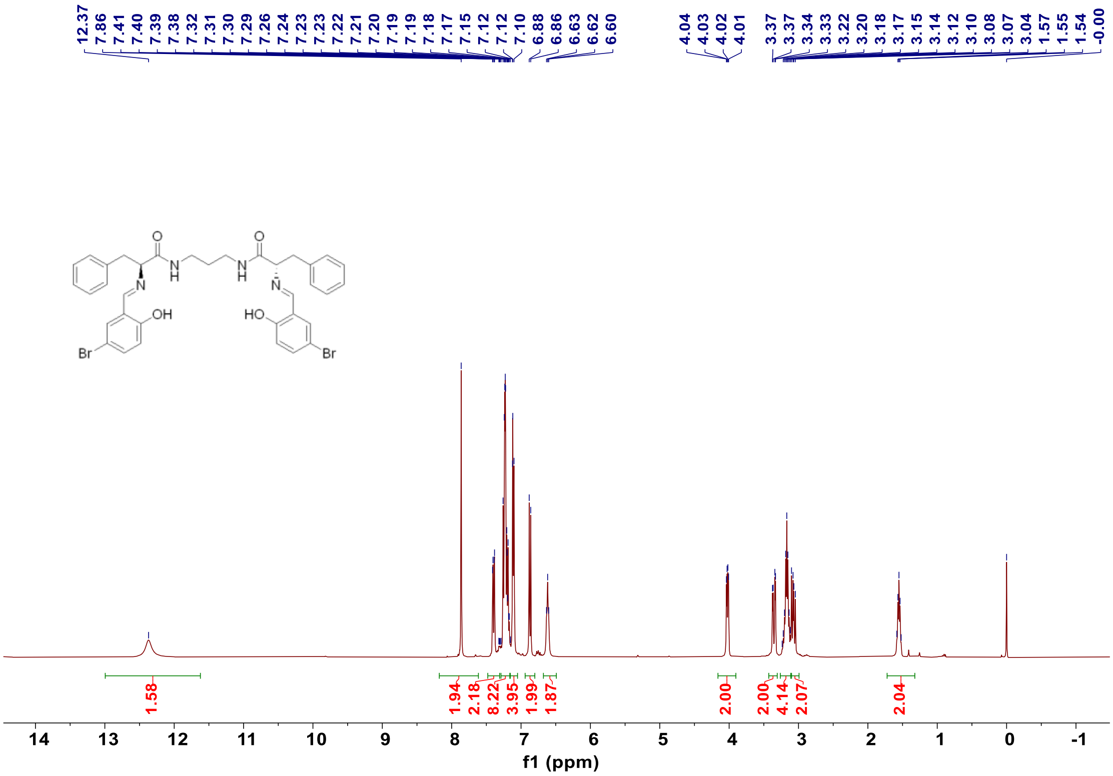

^{13}C{^{1}H} NMR (101 MHz, $CDCl_3$)

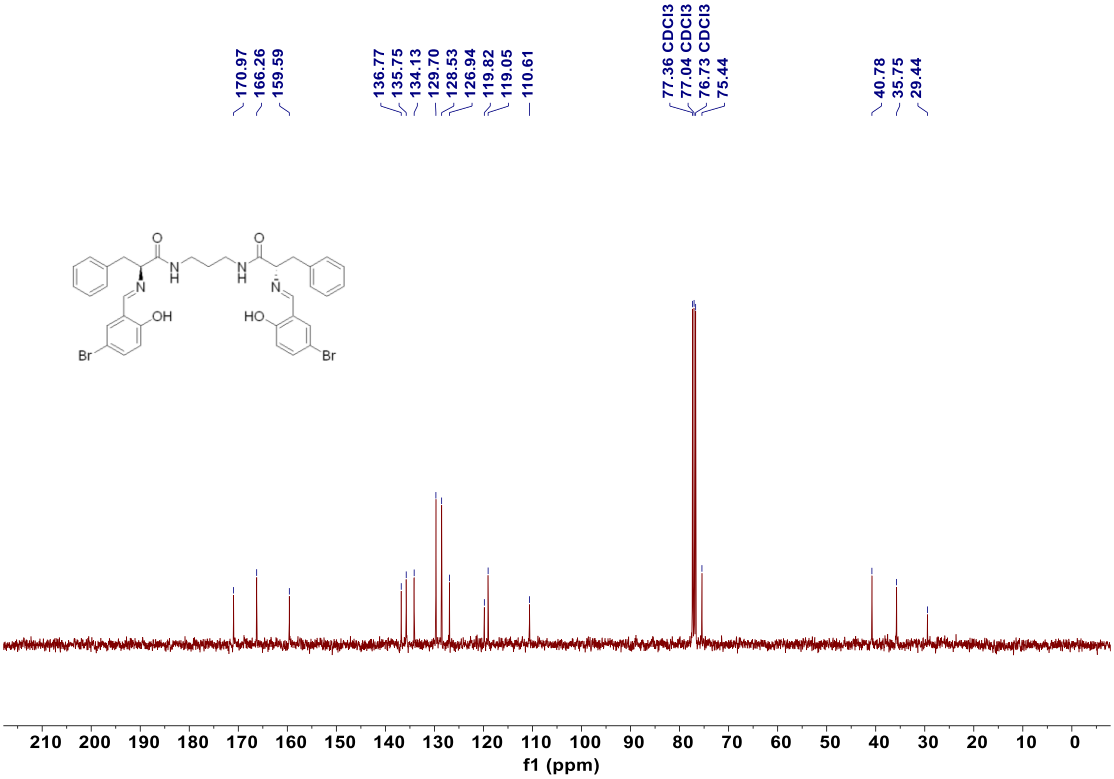

(2S,2'S)-*N*,*N*'-(Propane-1,3-diyl)bis(2-(((*E*)-2-hydroxy-5-methoxybenzylidene)amino)-3-phenylpropanamide) (**L6**)

^{1}H NMR (400 MHz, $CDCl_3$)

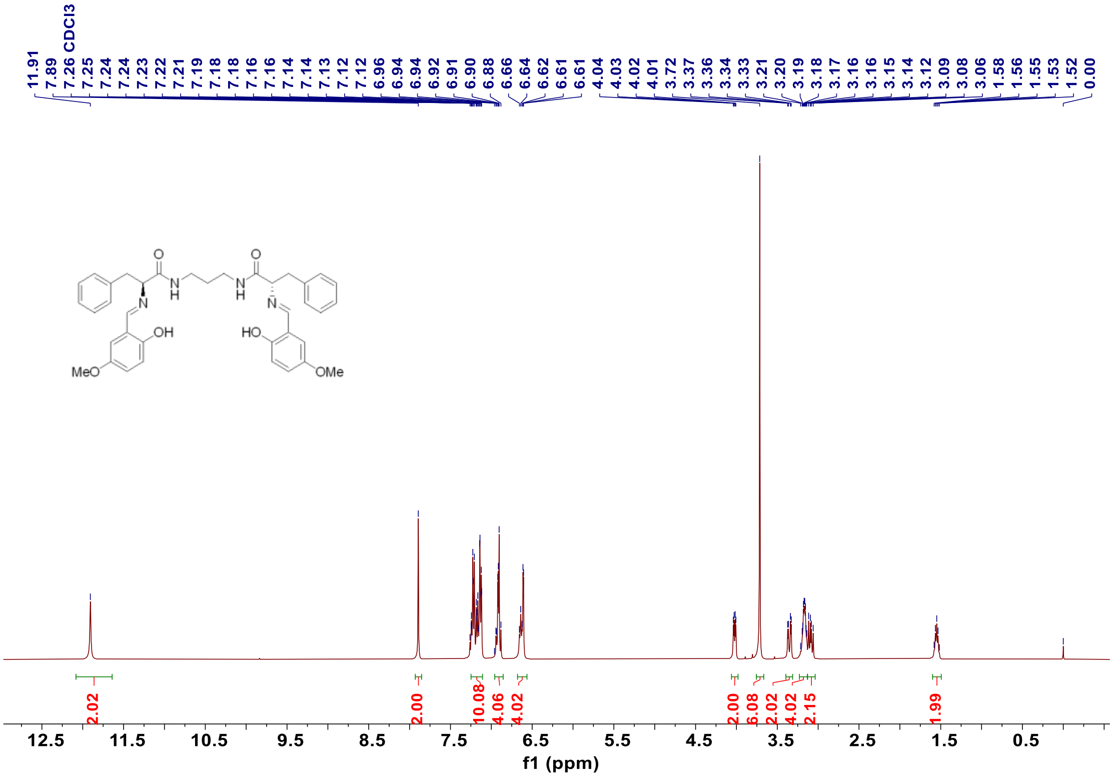

^{13}C{^{1}H} NMR (101 MHz, $CDCl_3$)

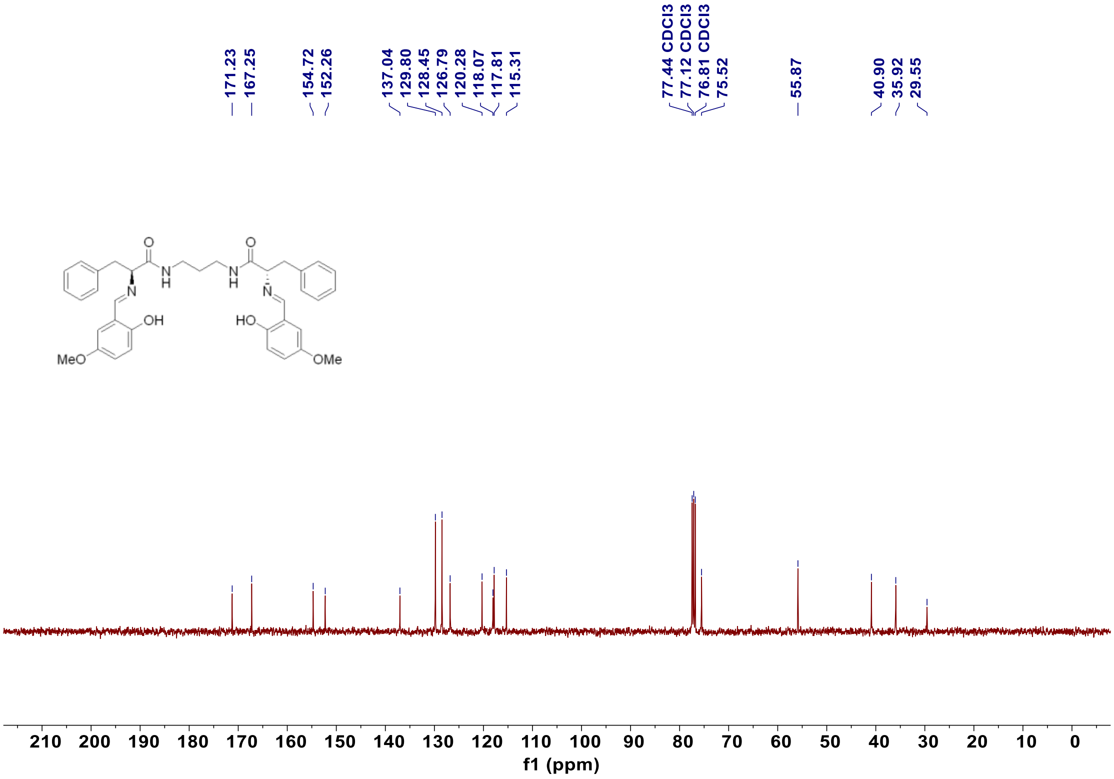

(2*S*,2'*S*)-*N*,*N*'-(Propane-1,3-diyl)bis(2-(((*E*)-3,5-di-*tert*-butyl-2-hydroxybenzylidene) amino)-3-phenylpropanamide) (**L7**)

^{1}H NMR (400 MHz, $CDCl_3$)

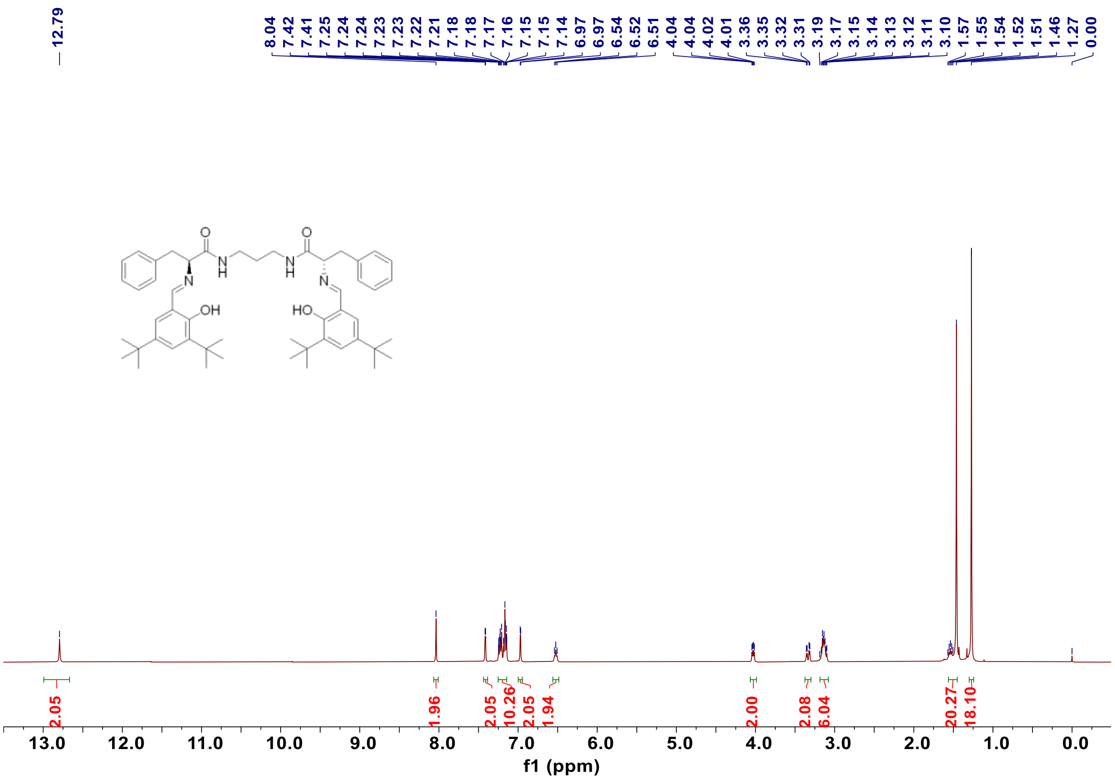

^{13}C{^{1}H} NMR (101 MHz, $CDCl_3$)

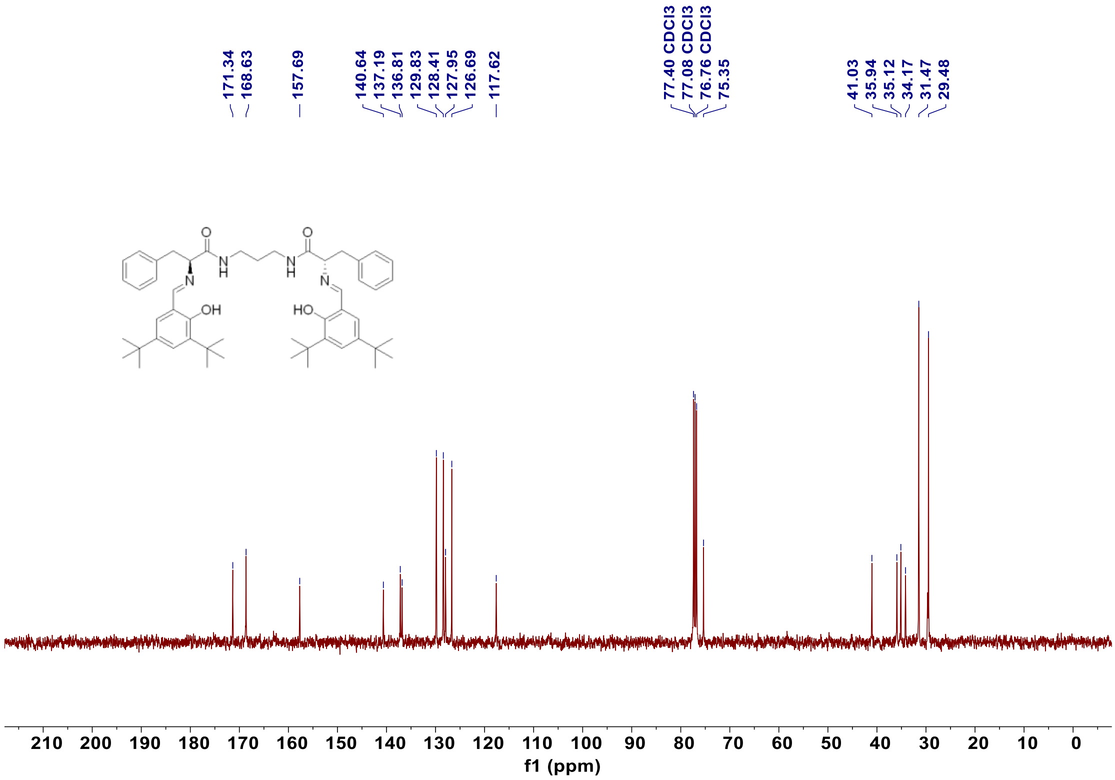

2. Copies of NMR spectra of products 3

(*R*)-2-(3-(6-Methoxy-1*H*-indol-3-yl)-3-phenylpropanoyl)pyridine 1-oxide (3aa)

^1H NMR (400 MHz, $CDCl_3$)

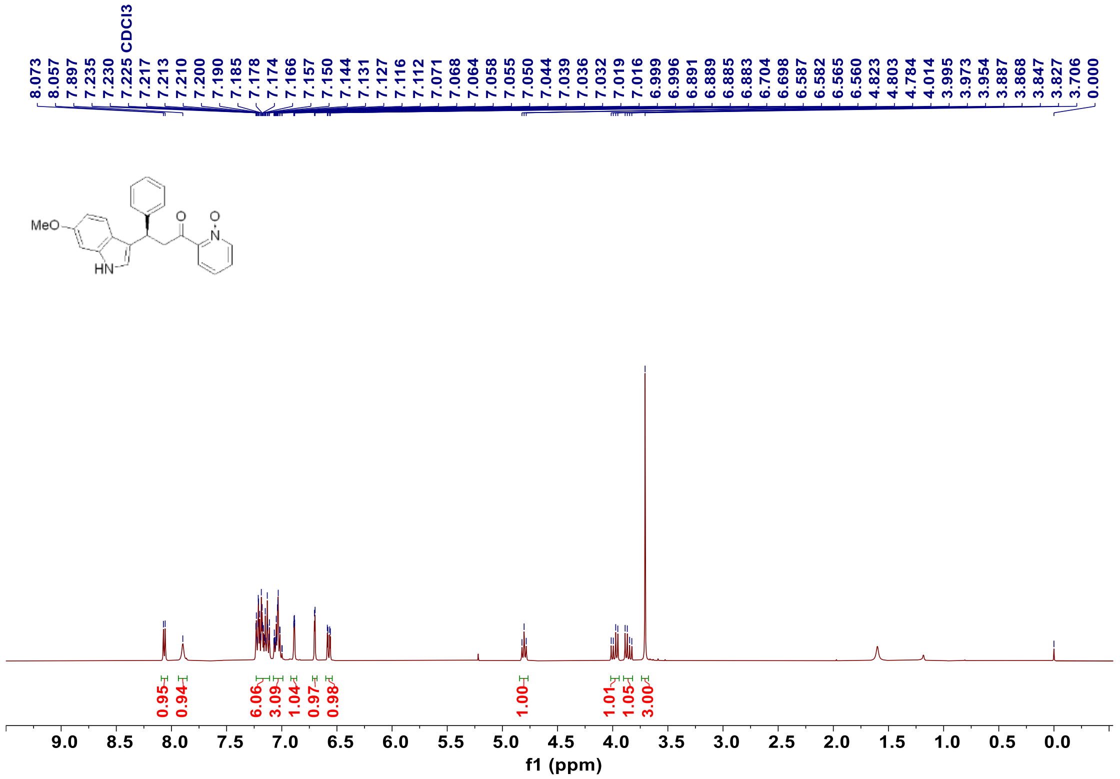

$^{13}C\{^1H\}$ NMR (101 MHz, $CDCl_3$)

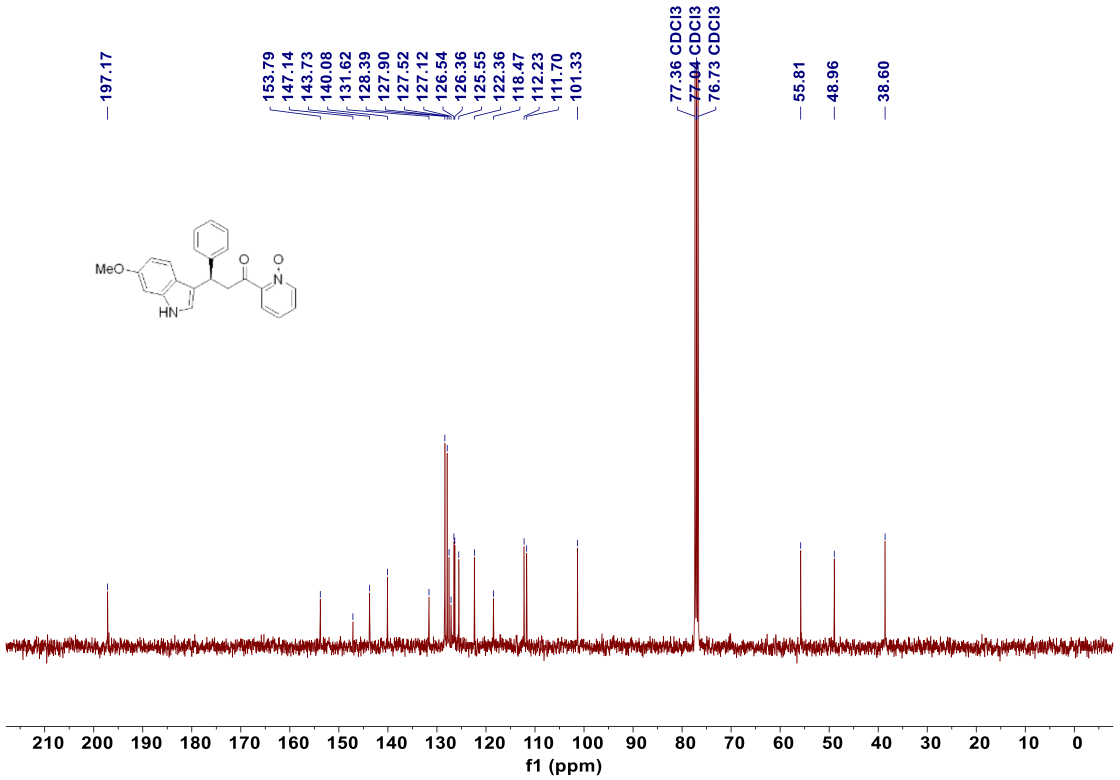

(*R*)-2-(3-(6-Methyl-1*H*-indol-3-yl)-3-phenylpropanoyl)pyridine 1-oxide (3ba)

^{1}H NMR (400 MHz, **DMSO-*d*₆**)

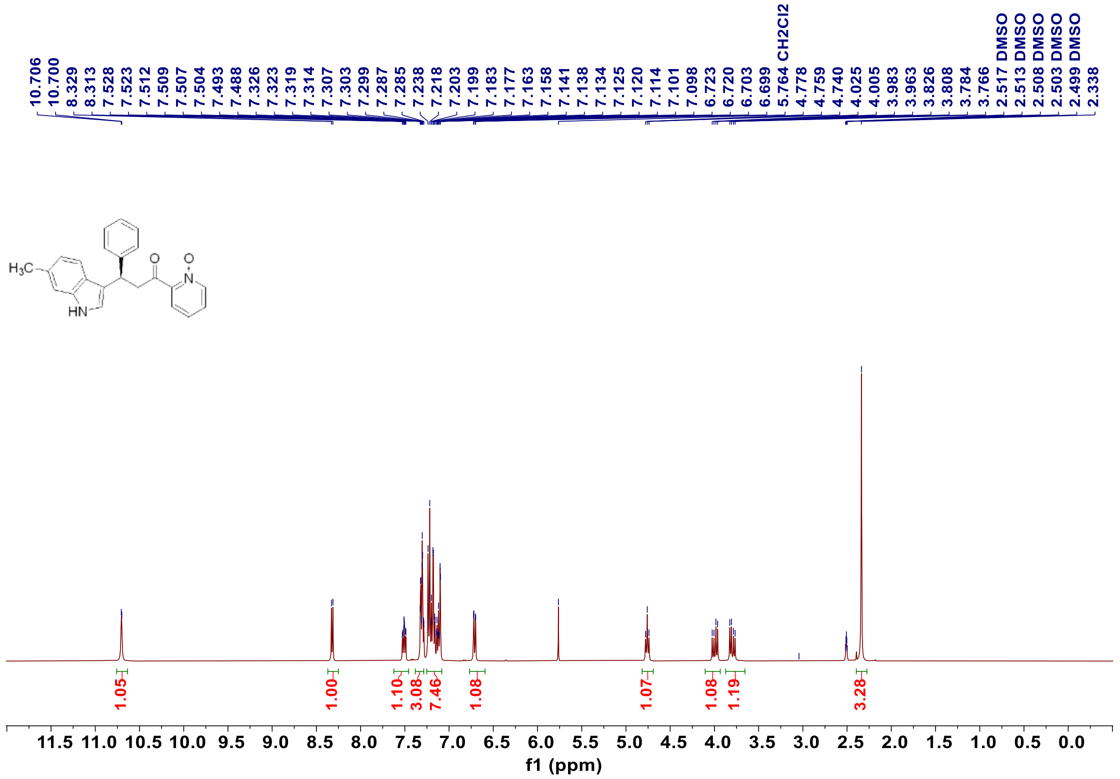

^{13}C{^{1}H} NMR (101 MHz, **DMSO-*d*₆**)

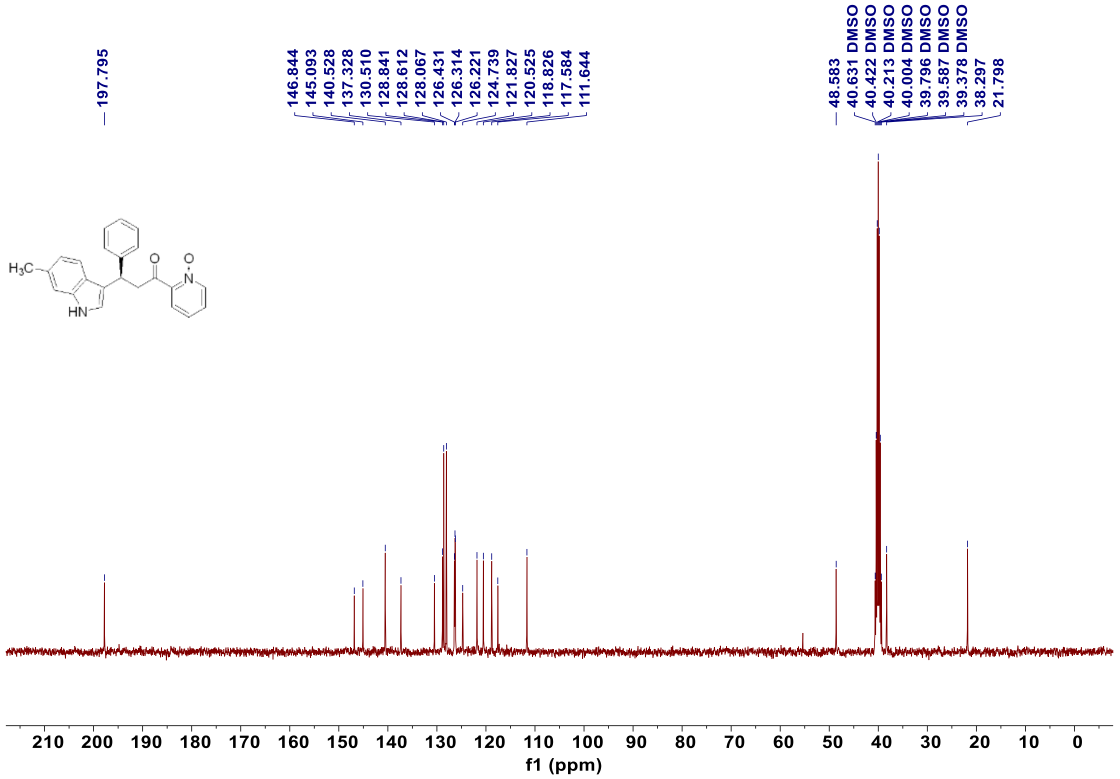

(*R*)-2-(3-(6-Bromo-1*H*-indol-3-yl)-3-phenylpropanoyl)pyridine 1-oxide (3ca)

^{1}H NMR (400 MHz, DMSO-d_6)

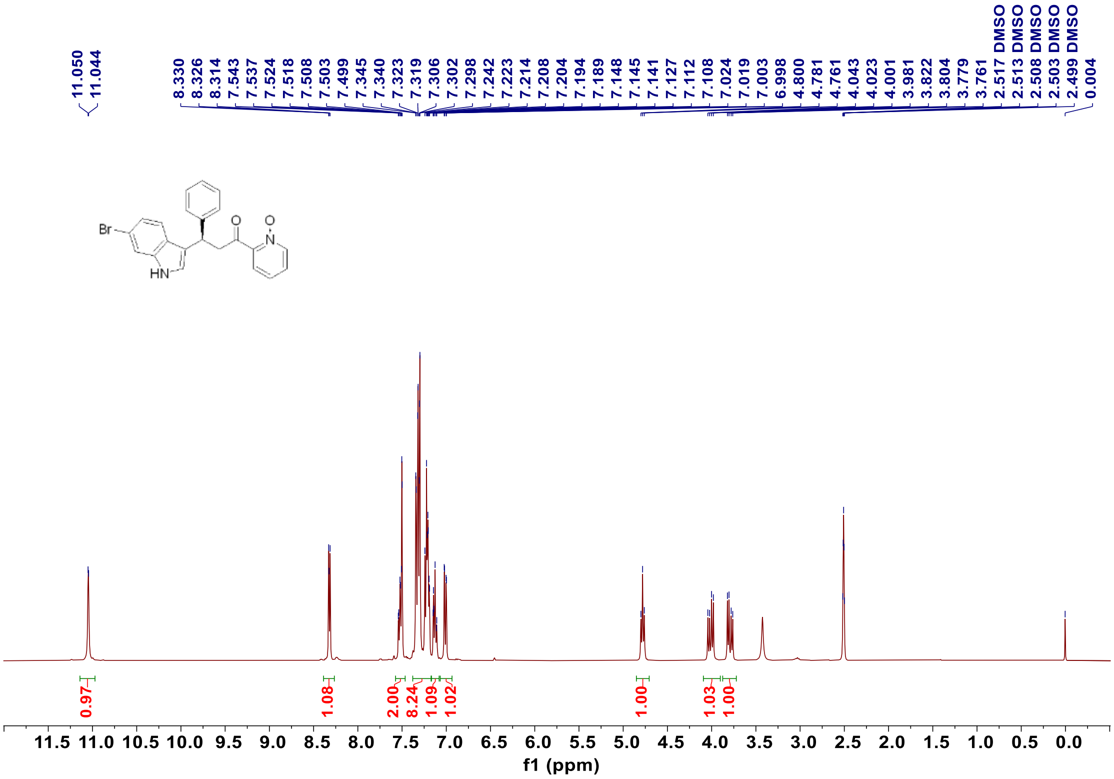

^{13}C{^{1}H} NMR (101 MHz, DMSO-d_6)

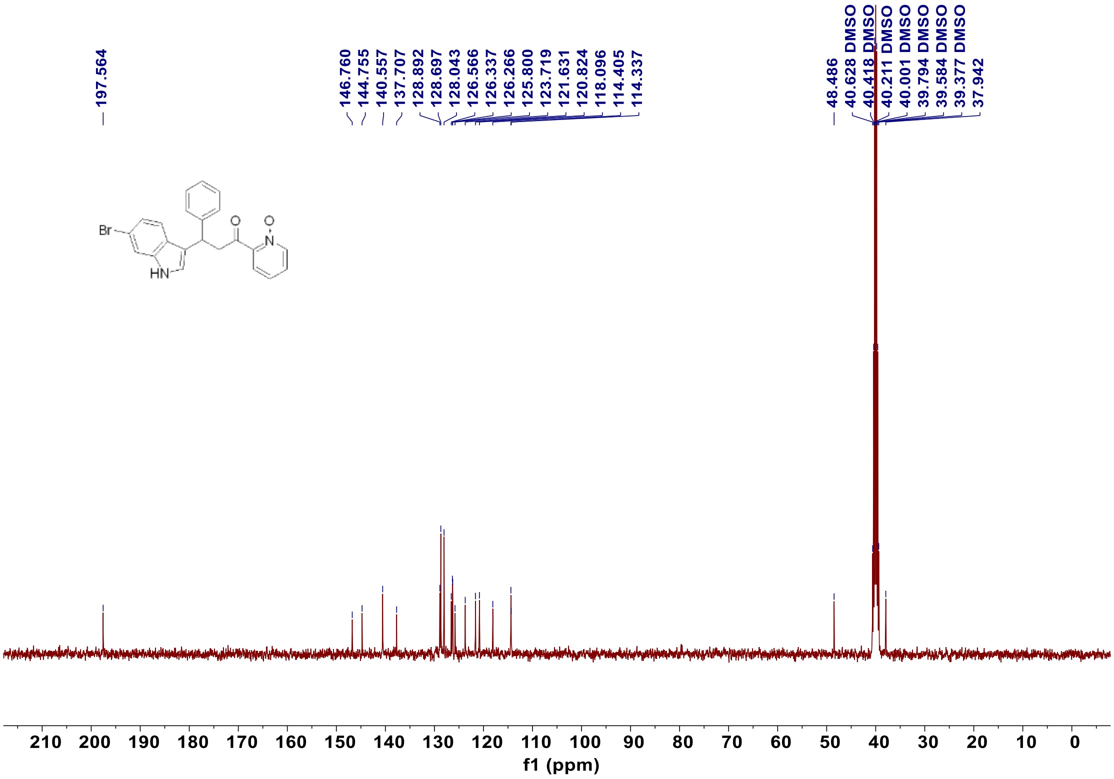

(*R*)-2-(3-(6-Fluoro-1*H*-indol-3-yl)-3-phenylpropanoyl)pyridine 1-oxide (3da)

^{1}H NMR (400 MHz, $CDCl_3$)

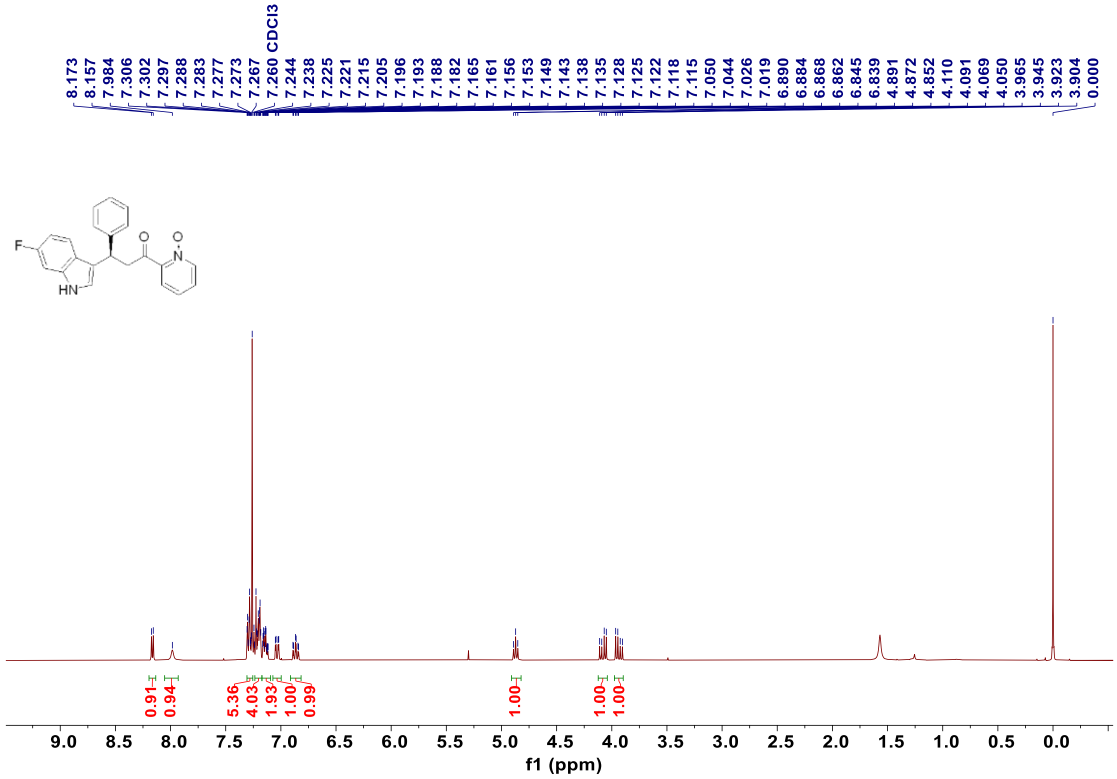

$^{13}C\{^{1}H\}$ NMR (101 MHz, **DMSO-*d*$_6$**)

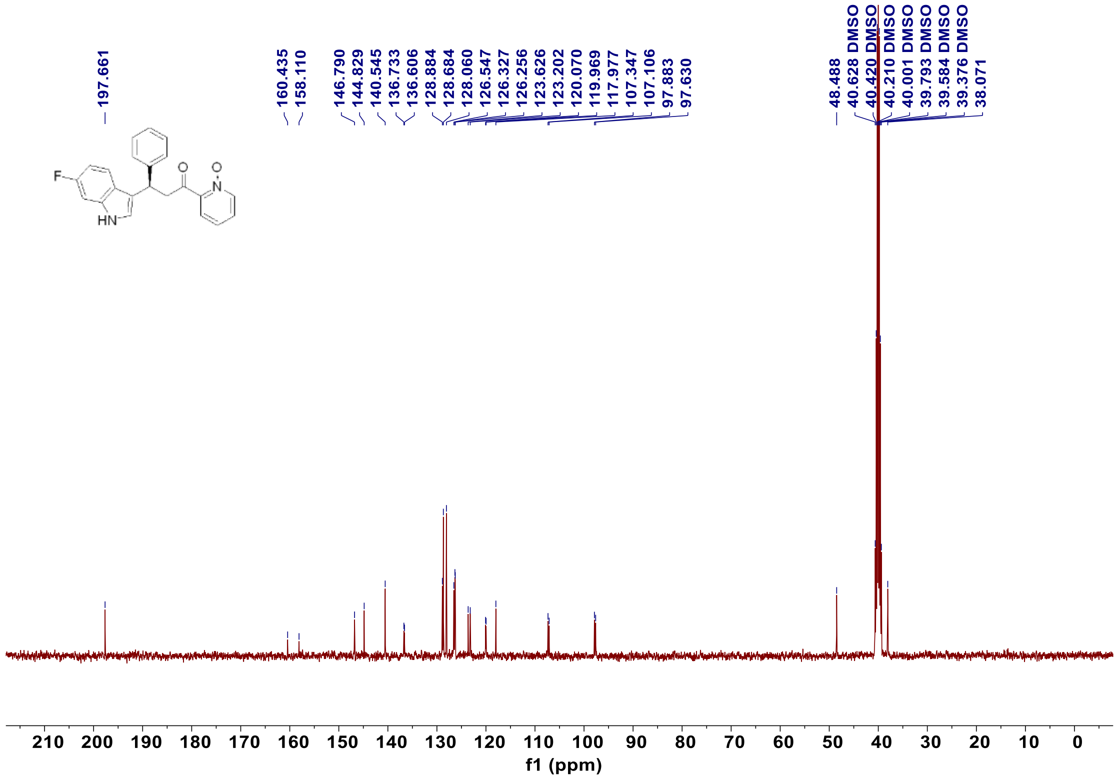

$^{19}F\{^1H\}$ NMR (377 MHz, $CDCl_3$)

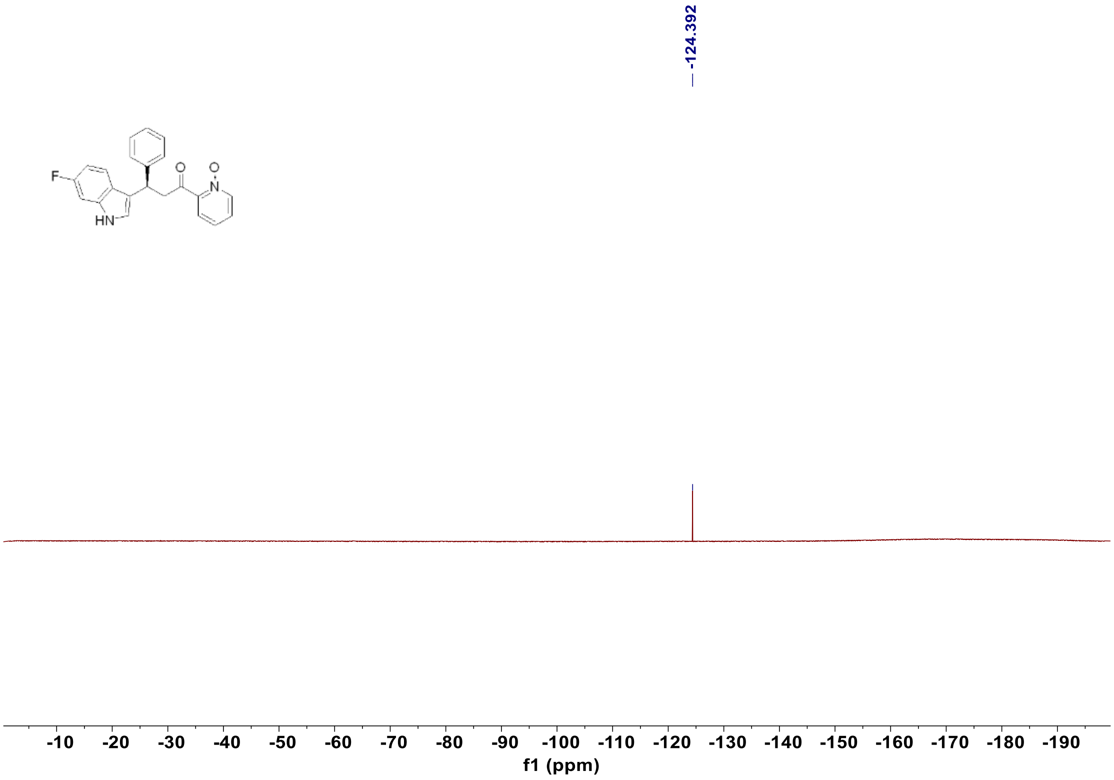

(*R*)-2-(3-(6-(Methoxycarbonyl)-1*H*-indol-3-yl)-3-phenylpropanoyl)pyridine 1-oxide (3ea)

^{1}H NMR (400 MHz, DMSO-*d*$_6$)

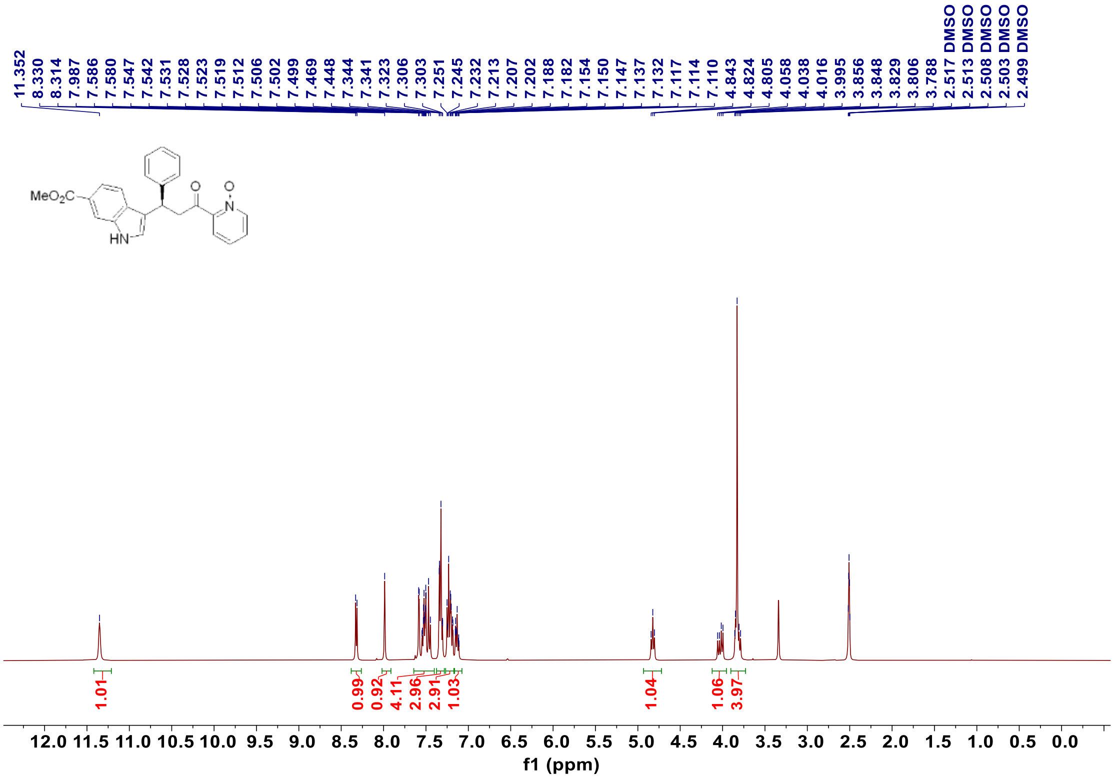

^{13}C{^{1}H} NMR (101 MHz, DMSO-*d*$_6$)

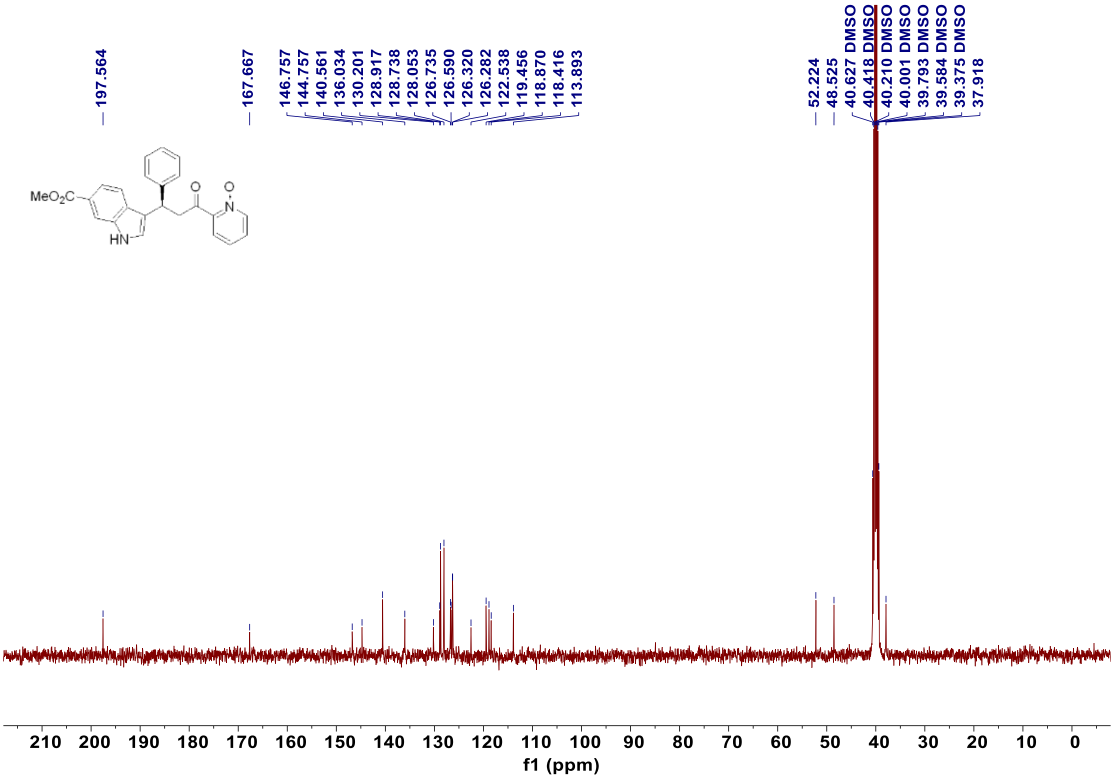

(*R*)-2-(3-(5-Methoxy-1*H*-indol-3-yl)-3-phenylpropanoyl)pyridine 1-oxide (3fa)

^{1}H NMR (400 MHz, $CDCl_3$)

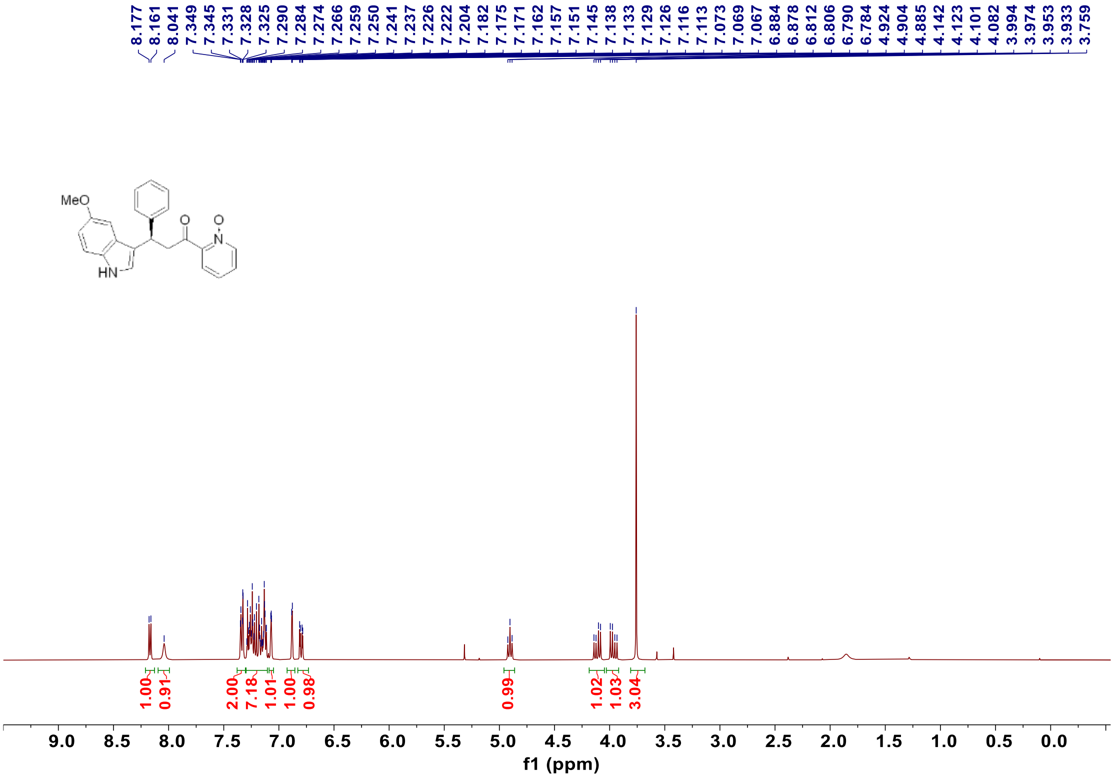

^{13}C{^{1}H} NMR (101 MHz, $CDCl_3$)

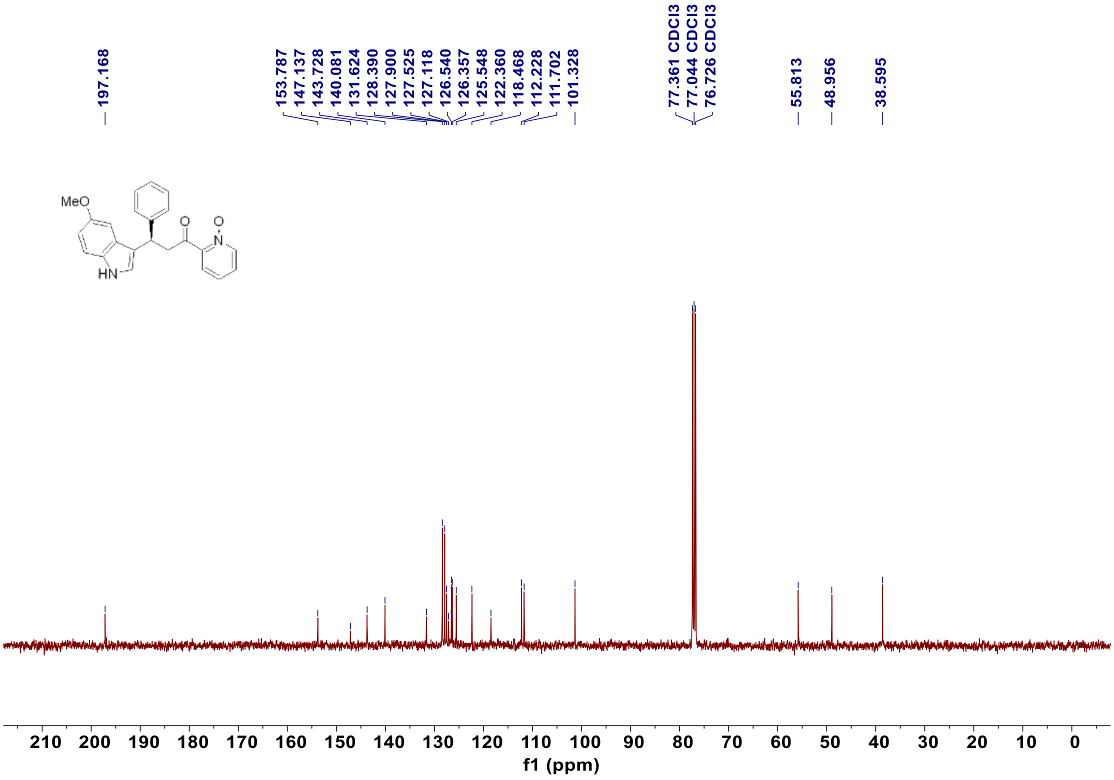

(*R*)-2-(3-(5-(Benzyloxy)-1*H*-indol-3-yl)-3-phenylpropanoyl)pyridine 1-oxide (3ga)

^{1}H NMR (400 MHz, $CDCl_3$)

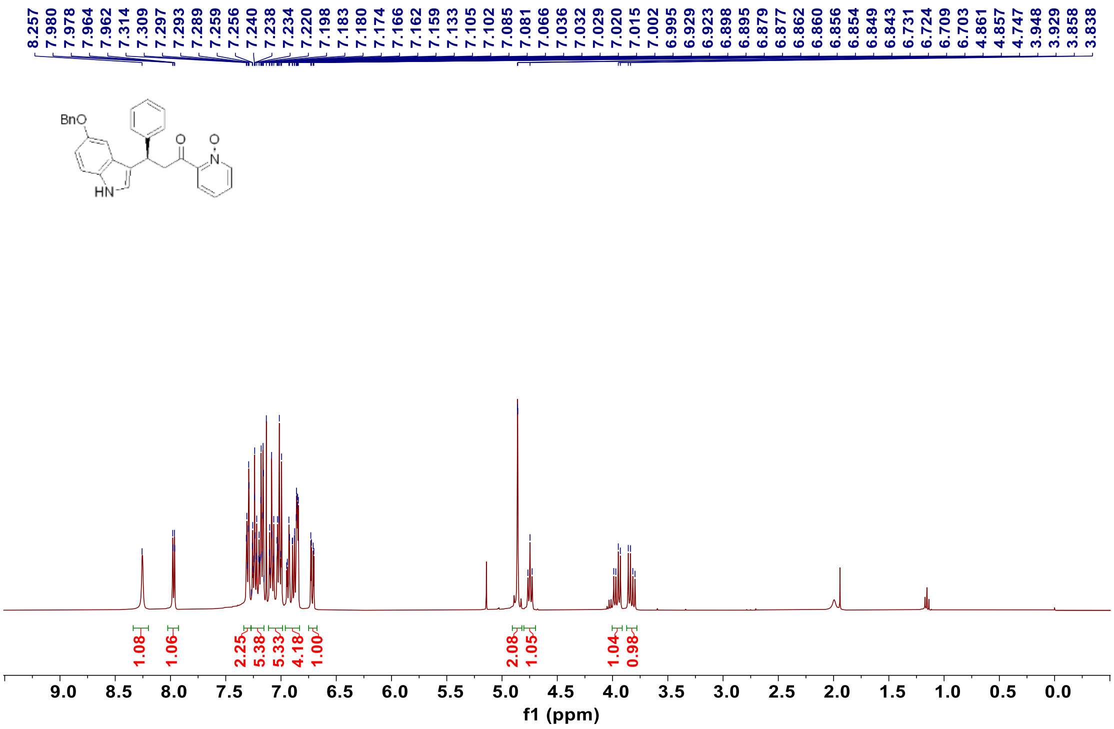

^{13}C NMR (101 MHz, **DMSO-*d*$_6$**)

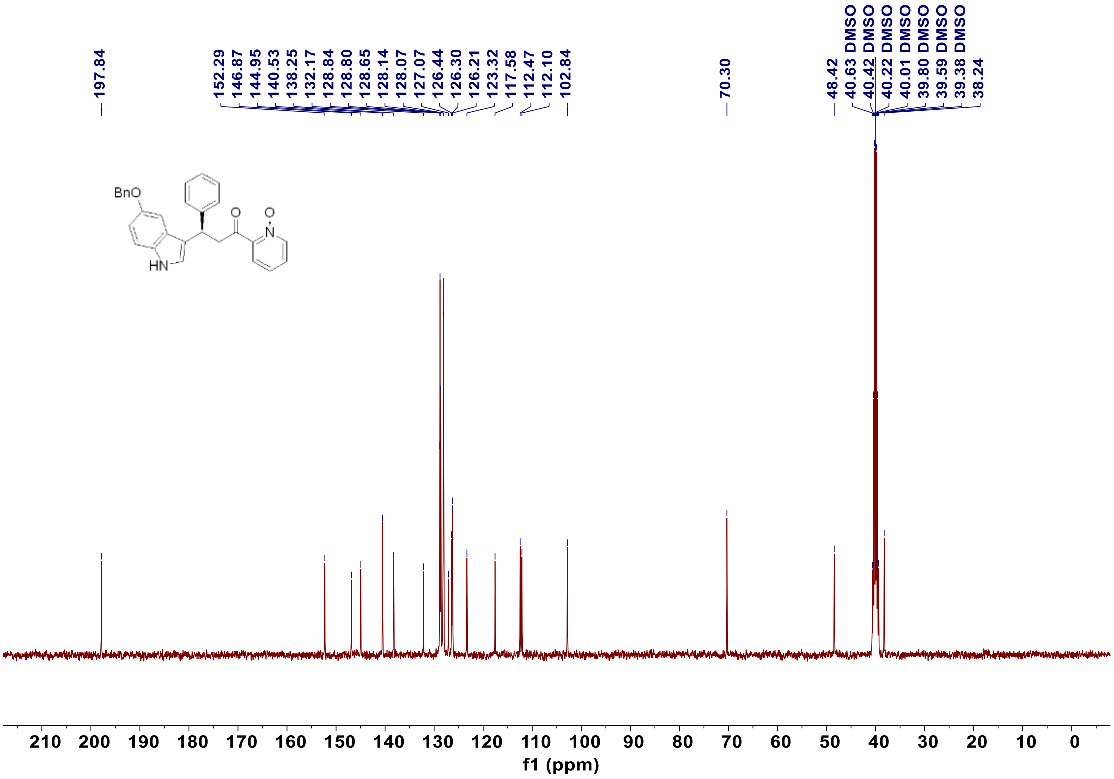

(*R*)-2-(3-(5-Chloro-1*H*-indol-3-yl)-3-phenylpropanoyl)pyridine 1-oxide (3ha)

^{1}H NMR (400 MHz, DMSO-d_6)

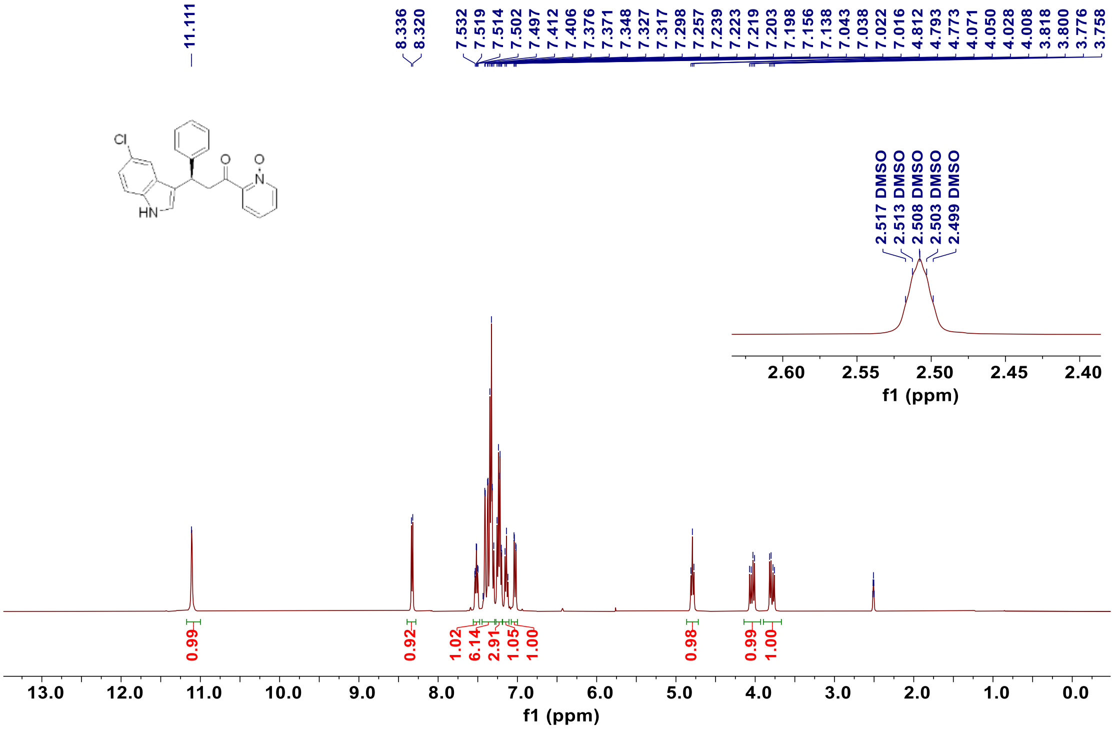

^{13}C{^{1}H} NMR (101 MHz, DMSO-d_6)

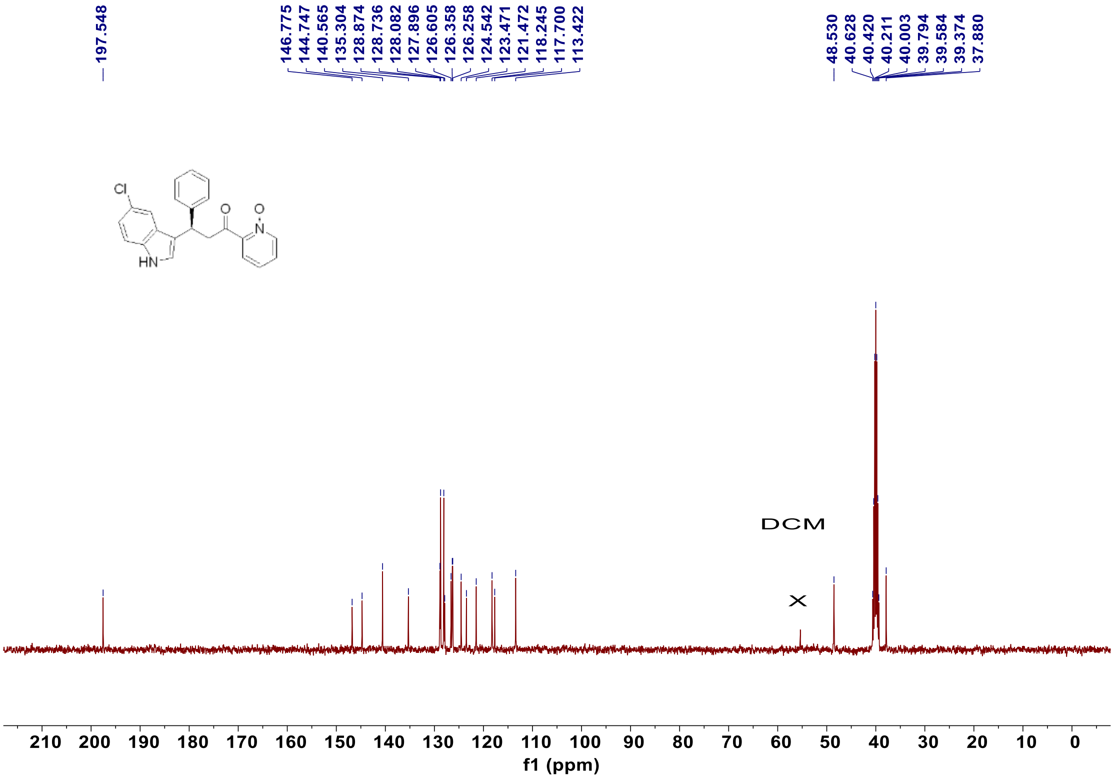

(*R*)-2-(3-(5-Fluoro-1*H*-indol-3-yl)-3-phenylpropanoyl)pyridine 1-oxide (3ia)

^{1}H NMR (400 MHz, 101 MHz, DMSO-d_6)

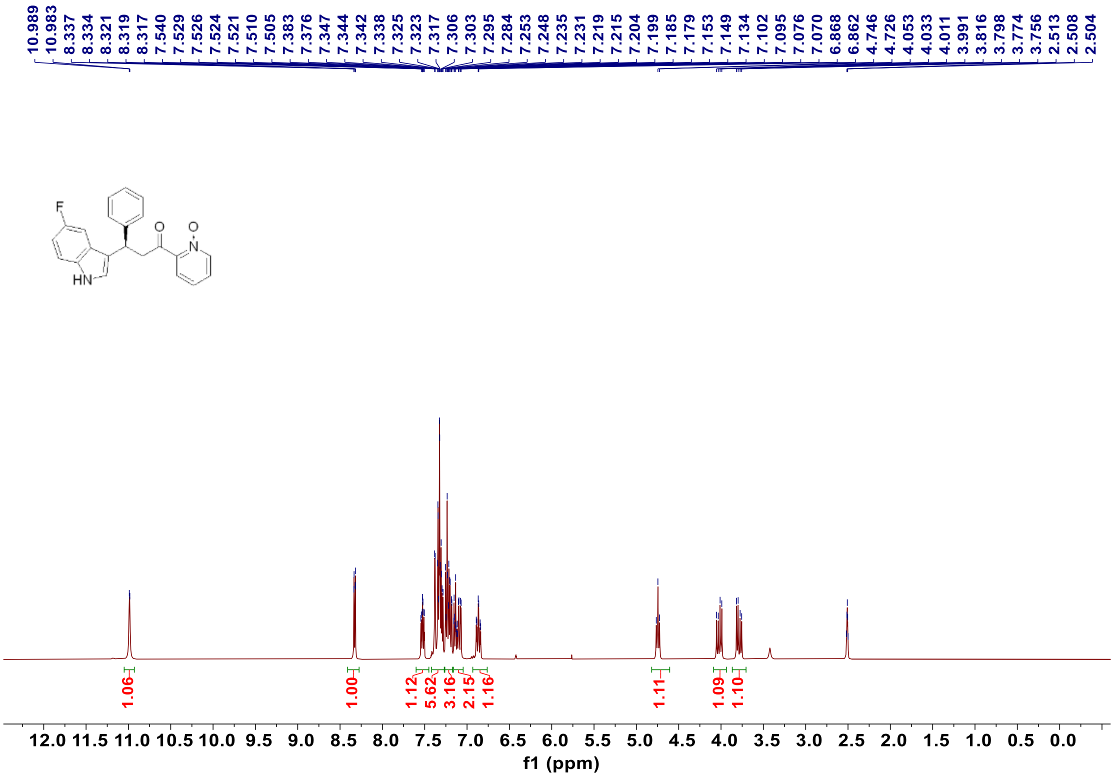

^{13}C{^{1}H} NMR (101 MHz, DMSO-d_6)

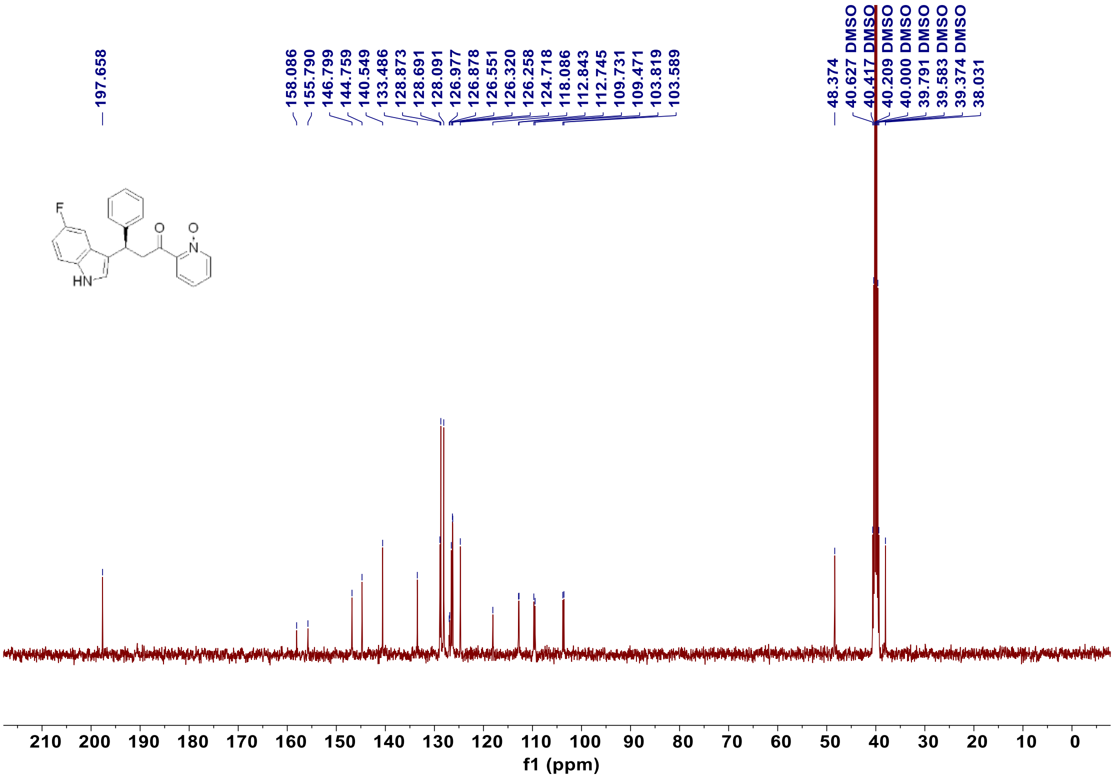

$^{19}F\{^{1}H\}$ NMR (377 MHz, DMSO-d_6)

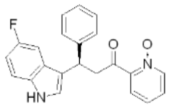

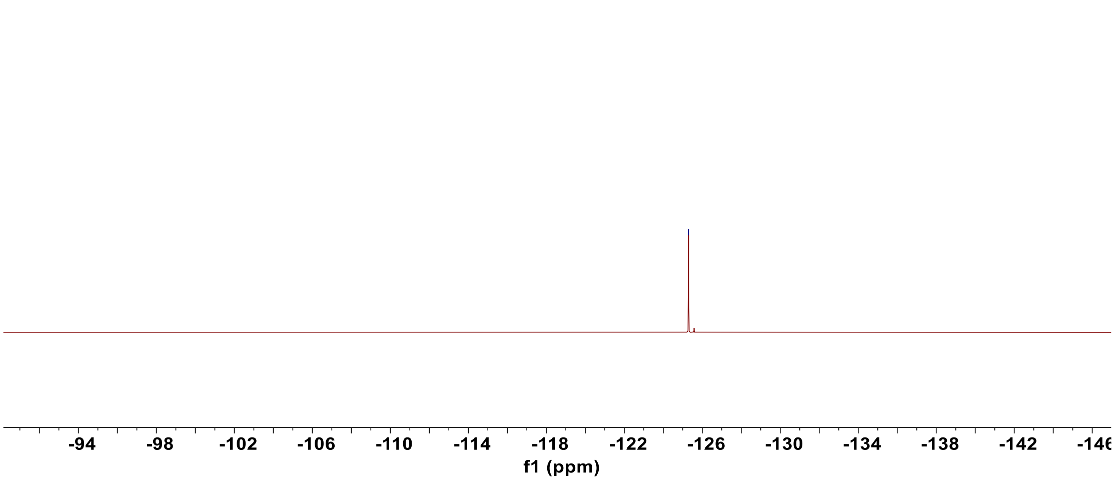

(*R*)-2-(3-(4-Methoxy-1*H*-indol-3-yl)-3-phenylpropanoyl)pyridine 1-oxide (3ja)

^{1}H NMR (400 MHz, $CDCl_3$)

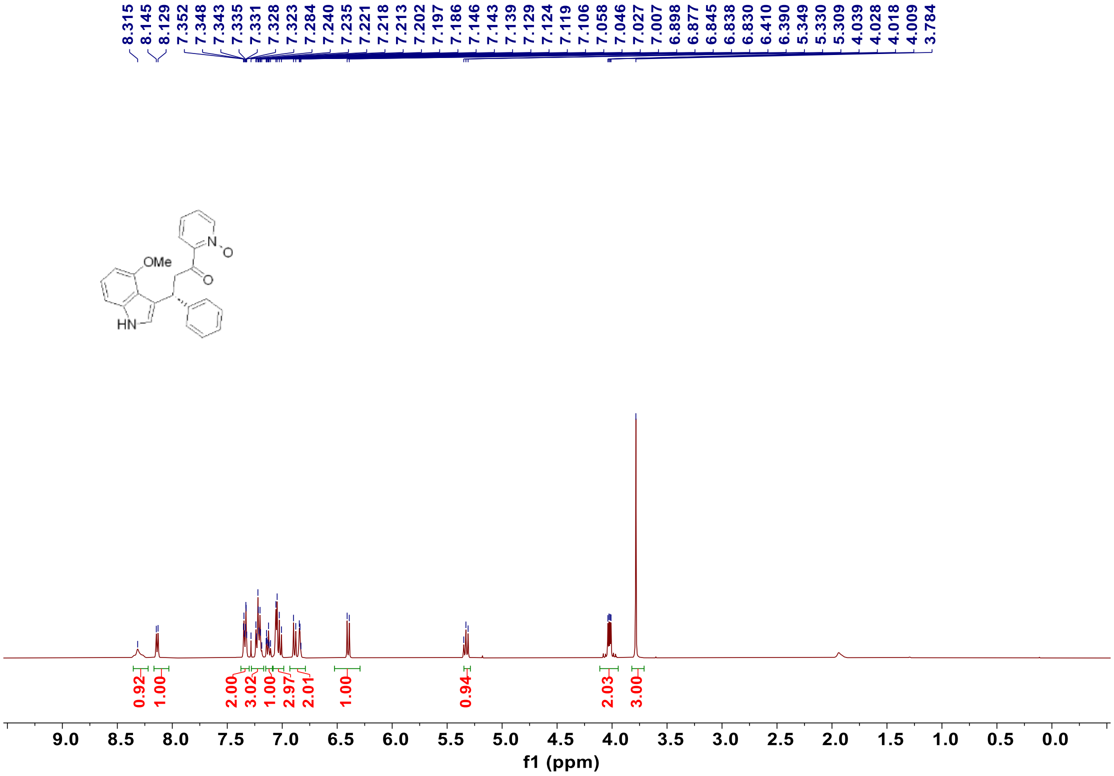

^{13}C{^{1}H} NMR (101 MHz, $CDCl_3$)

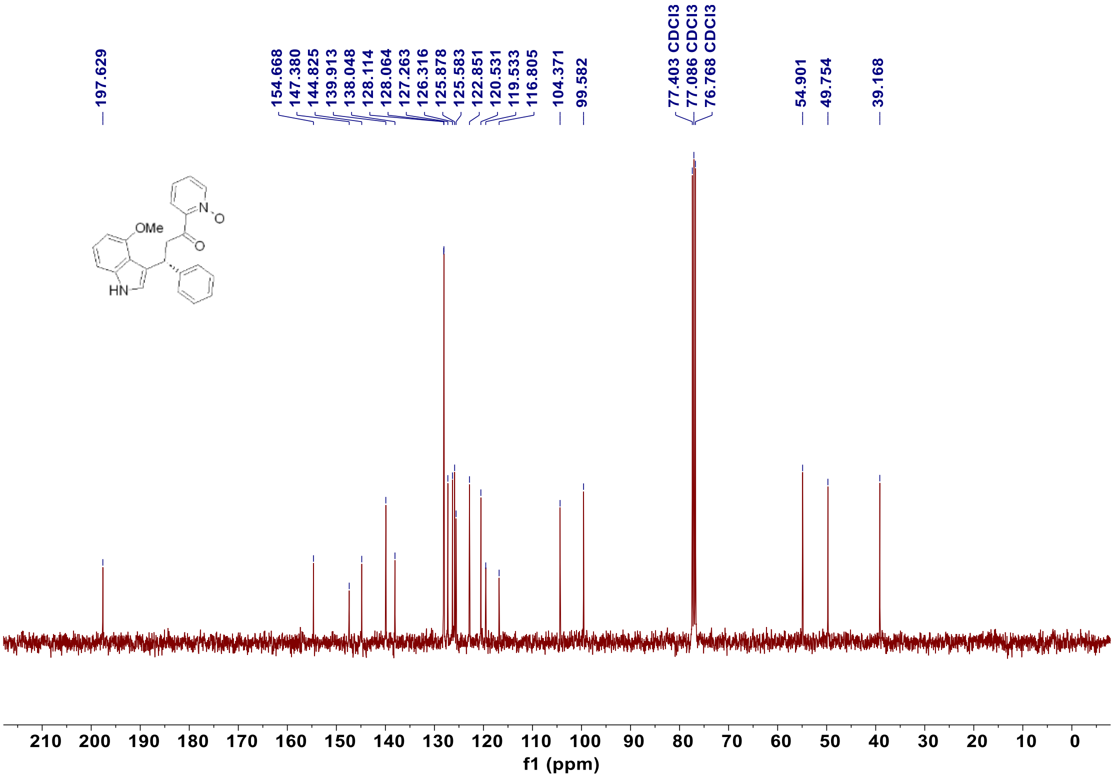

(*R*)-2-(3-(1-Methyl-1*H*-indol-3-yl)-3-phenylpropanoyl)pyridine 1-oxide (3ka)

^{1}H NMR (400 MHz, $CDCl_3$)

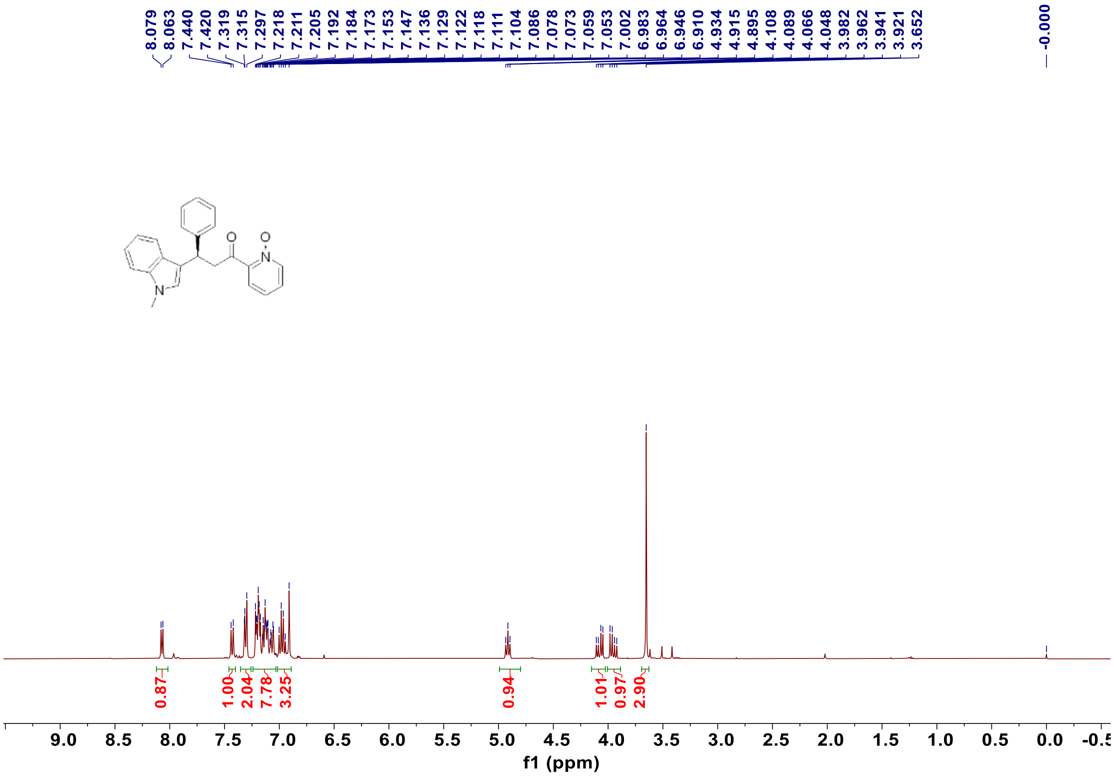

$^{13}C\{^{1}H\}$ NMR (101 MHz, $CDCl_3$)

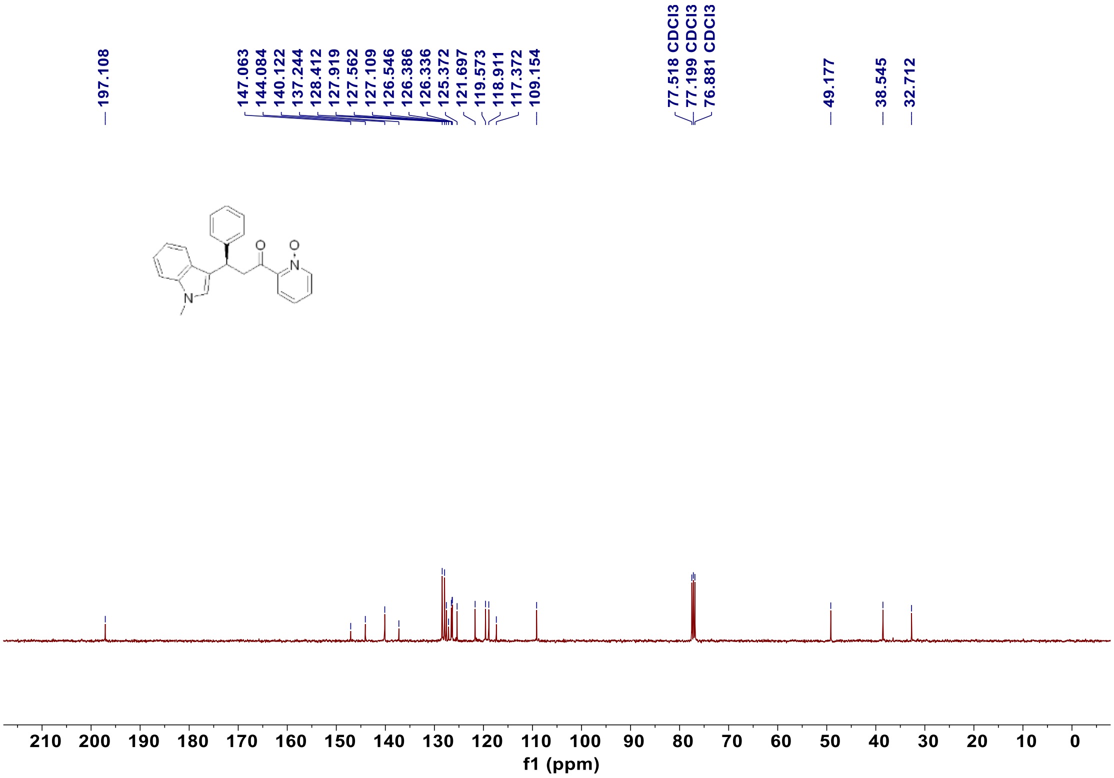

(*R*)-2-(3-(1*H*-Indol-3-yl)-3-phenylpropanoyl)pyridine 1-oxide (3ma)

^{1}H NMR (400 MHz, $CDCl_3$)

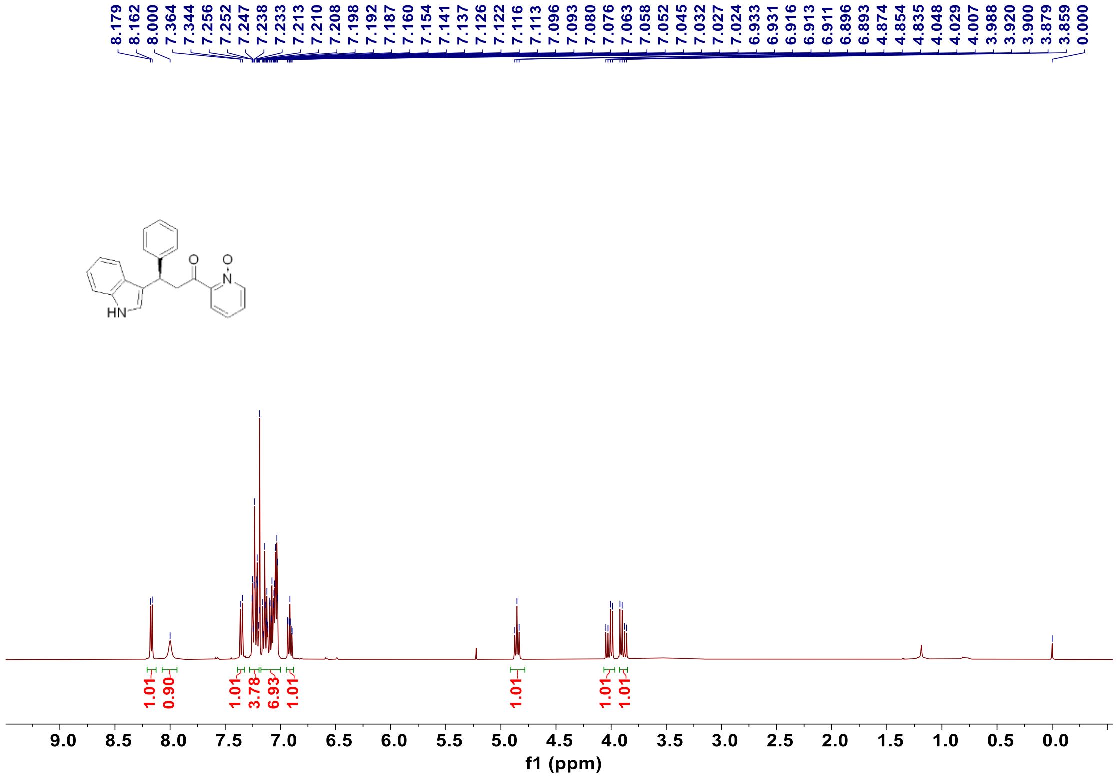

^{13}C{^{1}H} NMR (101 MHz, $CDCl_3$)

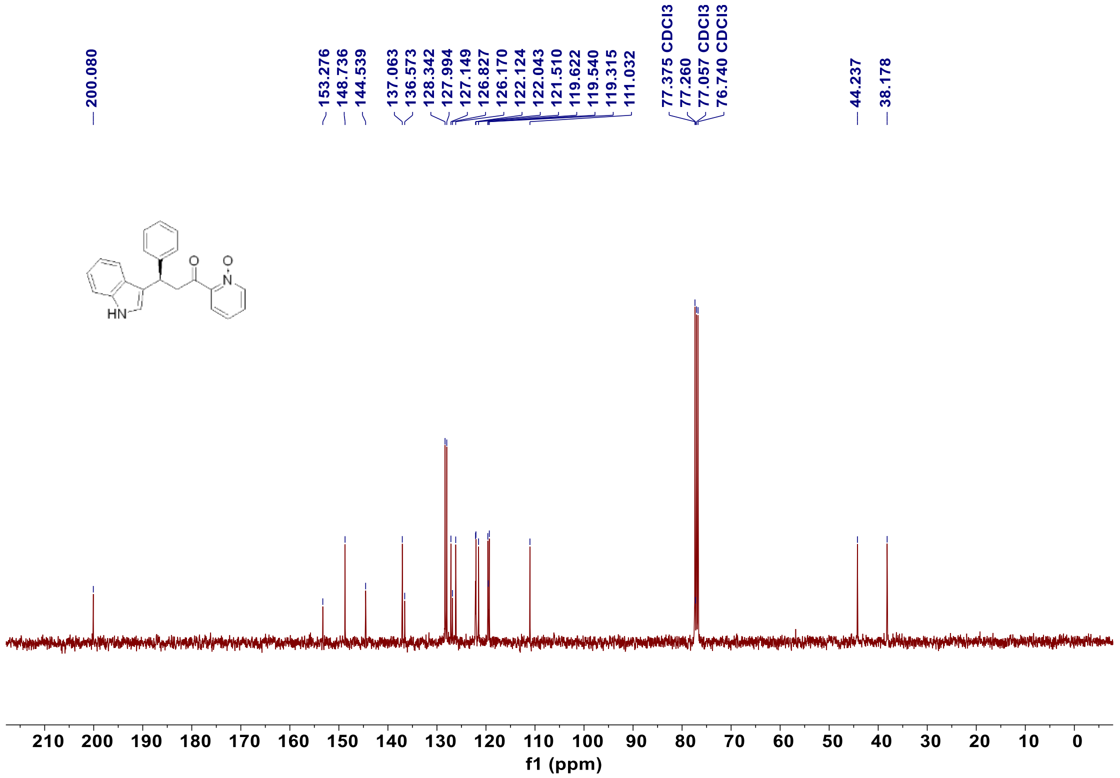

(*R*)-2-(3-(6-Methoxy-1*H*-indol-3-yl)-3-(4-methoxyphenyl)propanoyl)pyridine 1-oxide (3ab)

^{1}H NMR (400 MHz, $CDCl_3$)

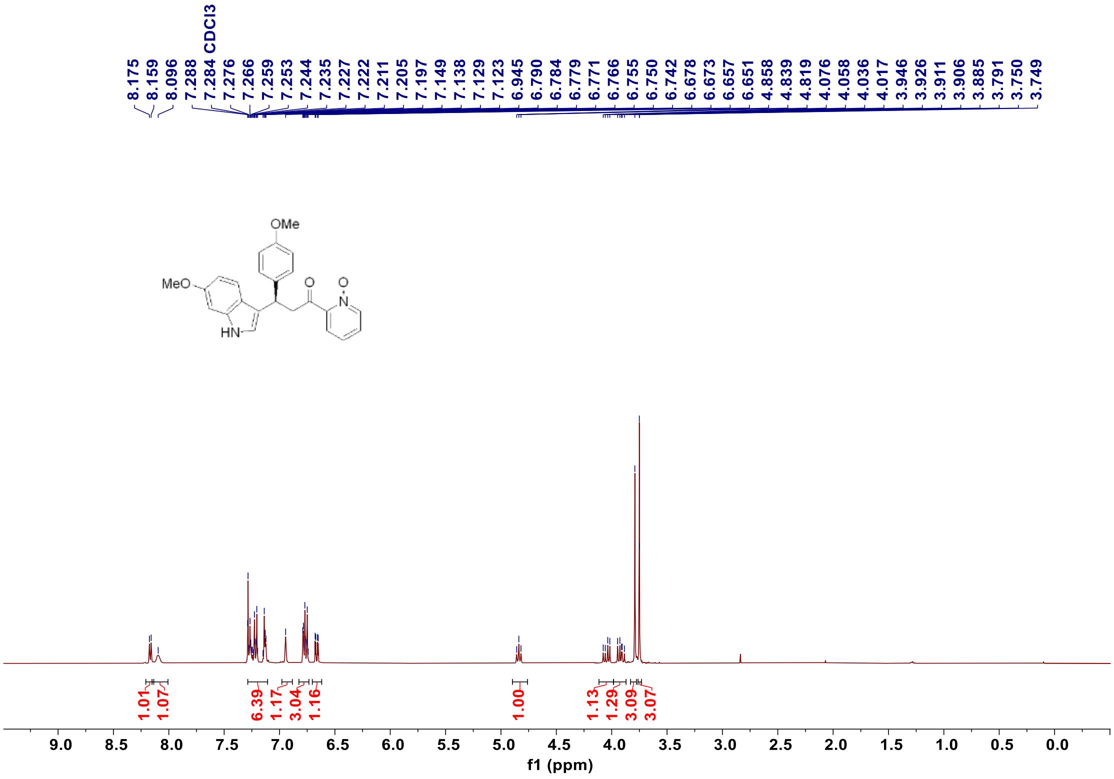

$^{13}C\{^{1}H\}$ NMR (101 MHz, $CDCl_3$)

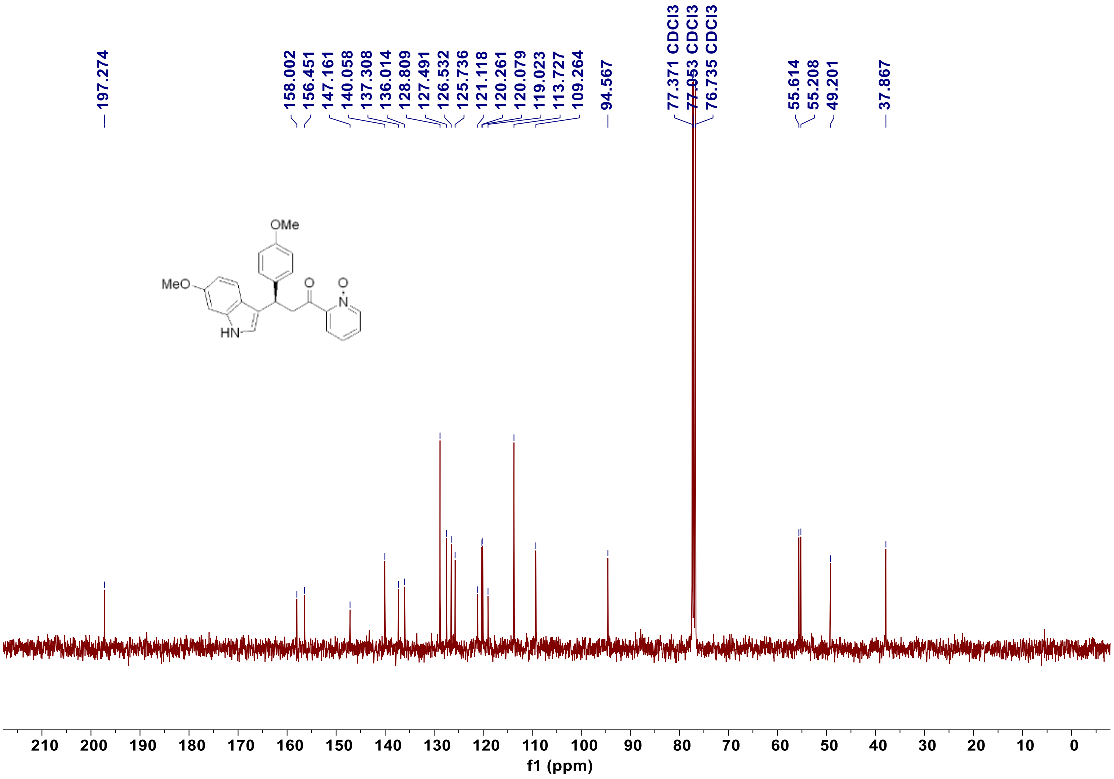

(*R*)-2-(3-(4-Fuorophenyl)-3-(6-methoxy-1*H*-indol-3-yl)propanoyl)pyridine 1-oxide (3ac)

^{1}H NMR (400 MHz, $CDCl_3$)

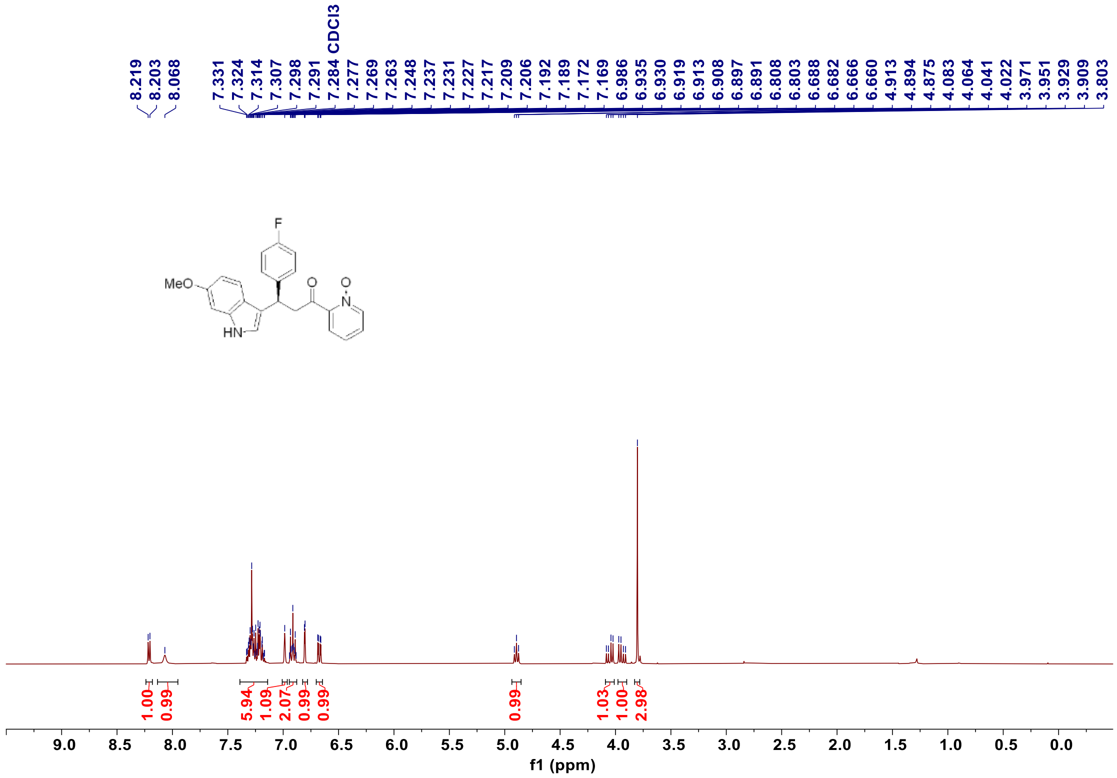

$^{13}C\{^{1}H\}$ NMR (101 MHz, $CDCl_3$)

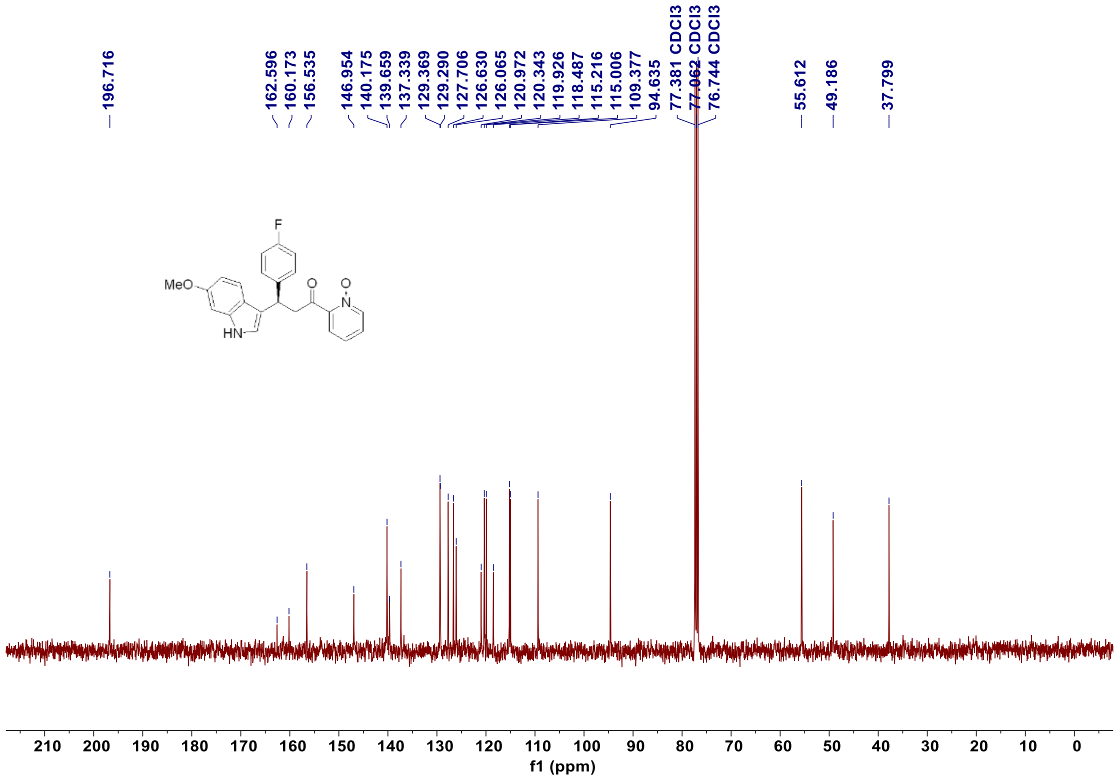

$^{19}F\{^1H\}$ NMR (377 MHz, $CDCl_3$)

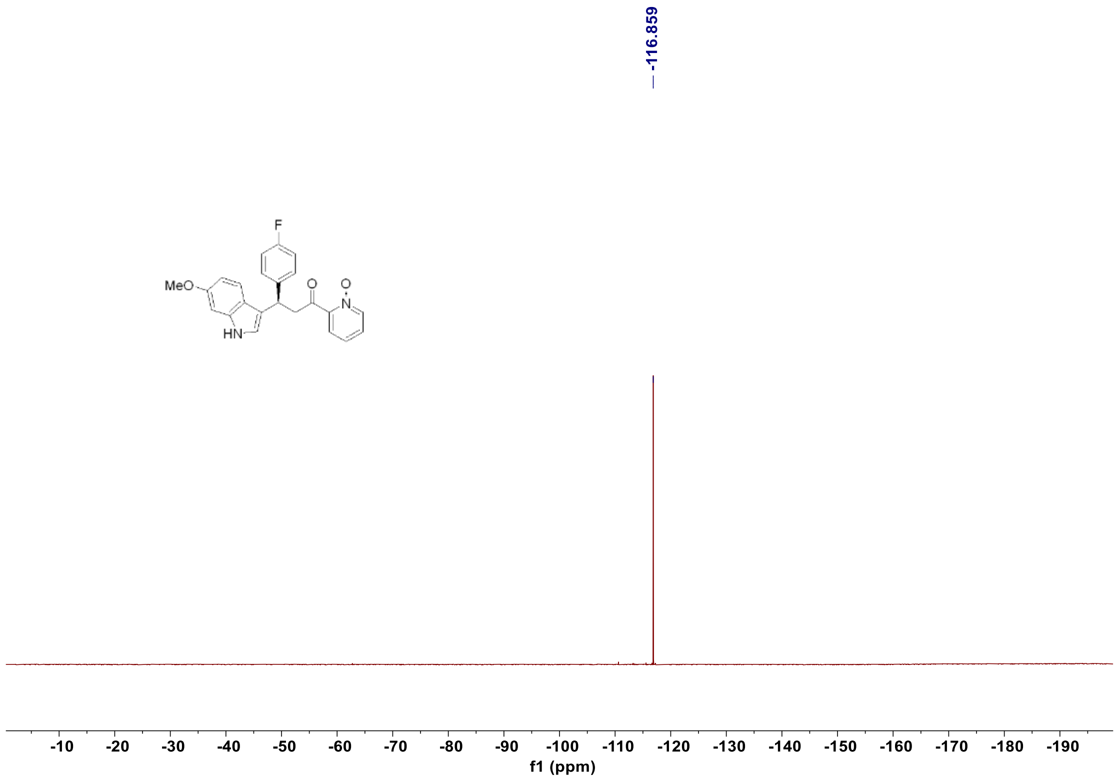

(*R*)-2-(3-(4-Chlorophenyl)-3-(6-methoxy-1*H*-indol-3-yl)propanoyl)pyridine 1-oxide (3ad)

^{1}H NMR (400 MHz, CDCl$_3$)

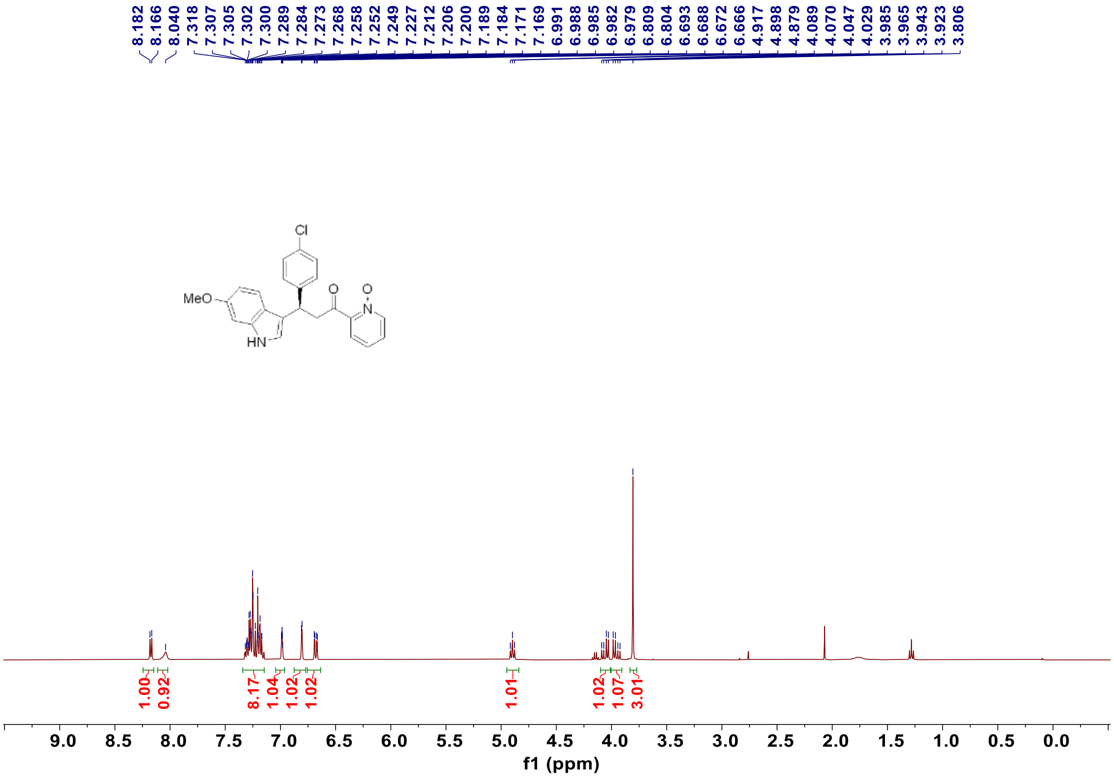

^{13}C{^{1}H} NMR (101 MHz, CDCl$_3$)

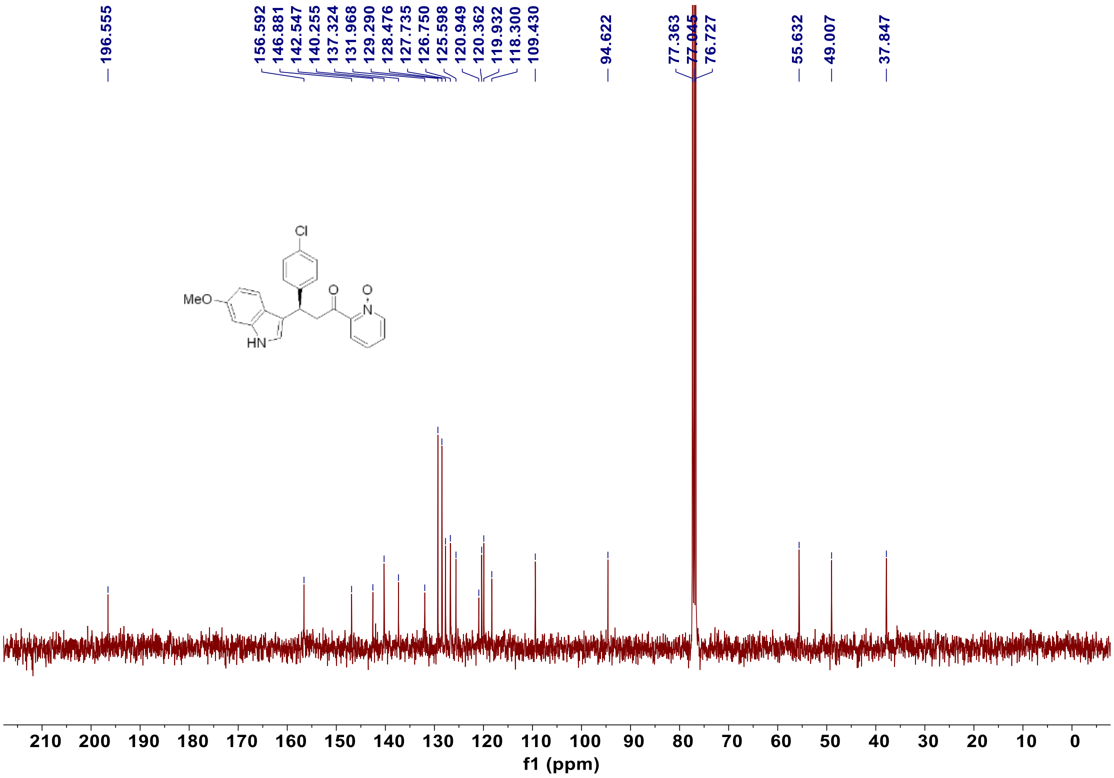

(*R*)-2-(3-(4-Bromophenyl)-3-(6-methoxy-1*H*-indol-3-yl)propanoyl)pyridine 1-oxide (3ae)

^{1}H NMR (400 MHz, $CDCl_3$)

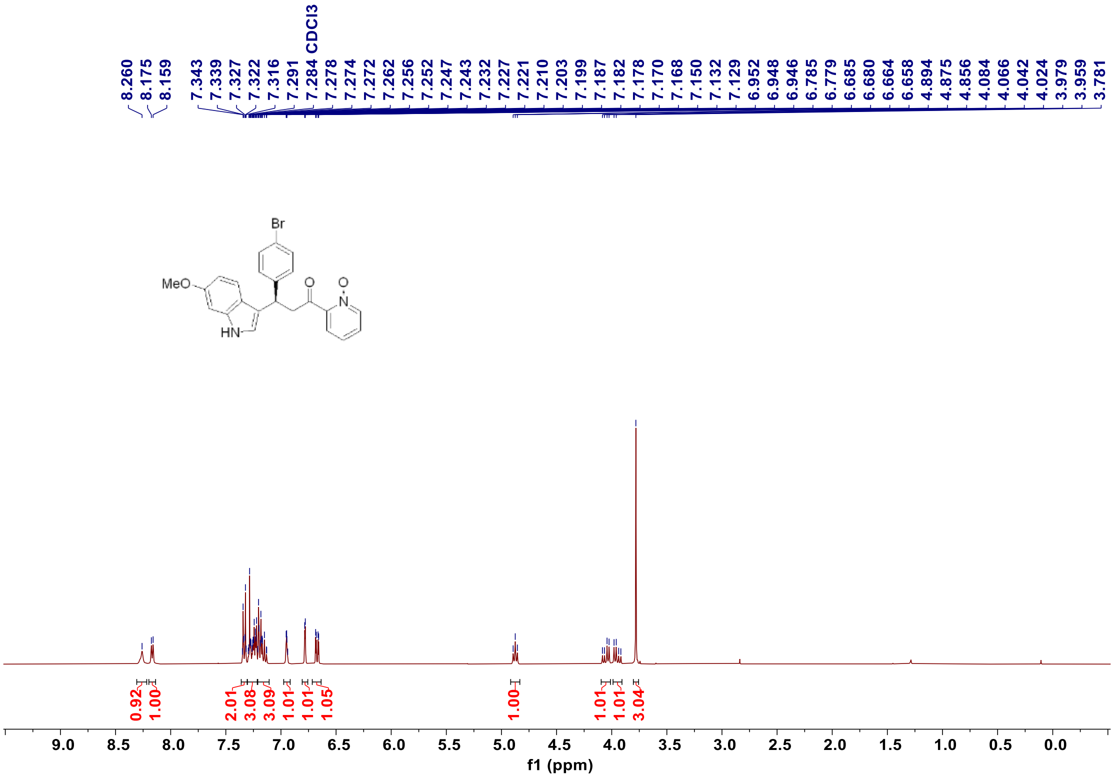

^{13}C{^{1}H} NMR (101 MHz, $CDCl_3$)

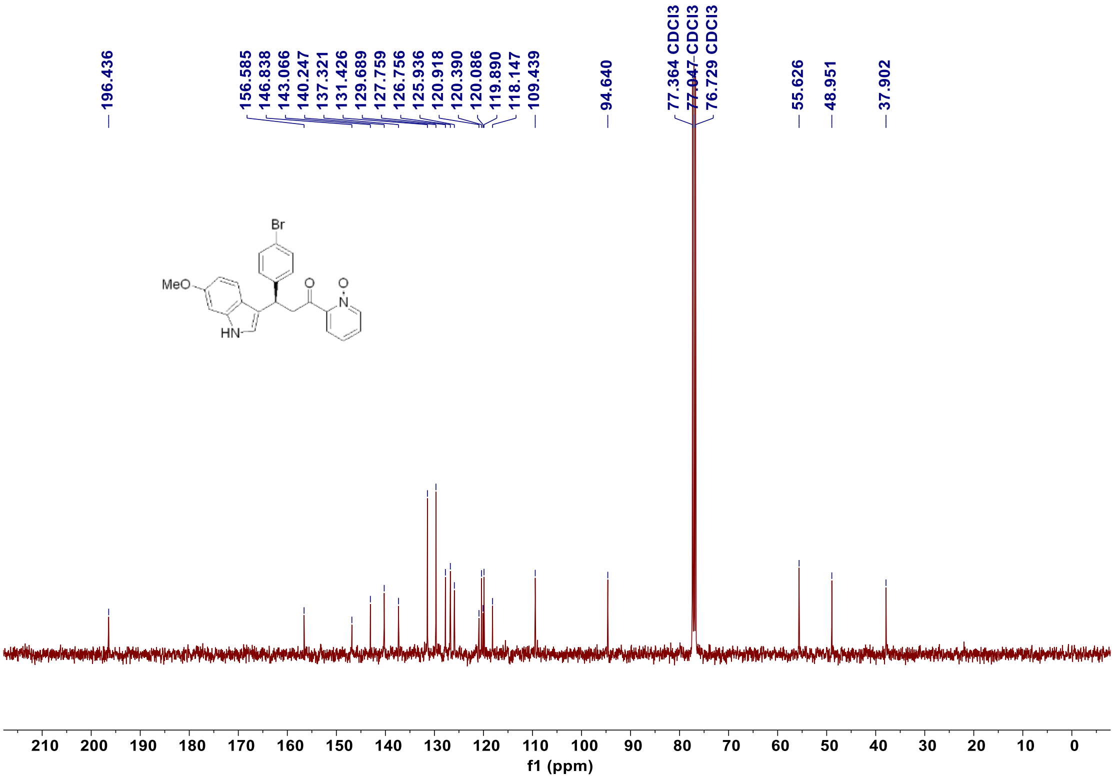

(*R*)-2-(3-(4-Cyanophenyl)-3-(6-methoxy-1*H*-indol-3-yl)propanoyl)pyridine 1-oxide (3af)

^{1}H NMR (400 MHz, $CDCl_3$)

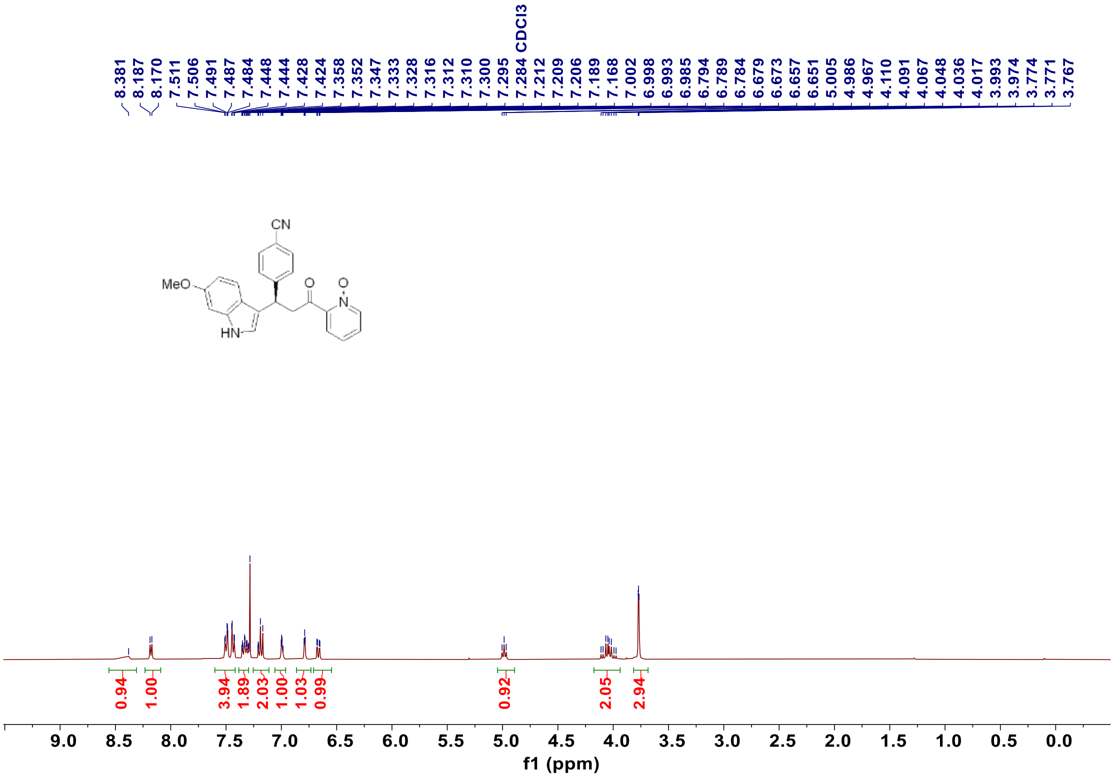

$^{13}C\{^{1}H\}$ NMR (101 MHz, $CDCl_3$)

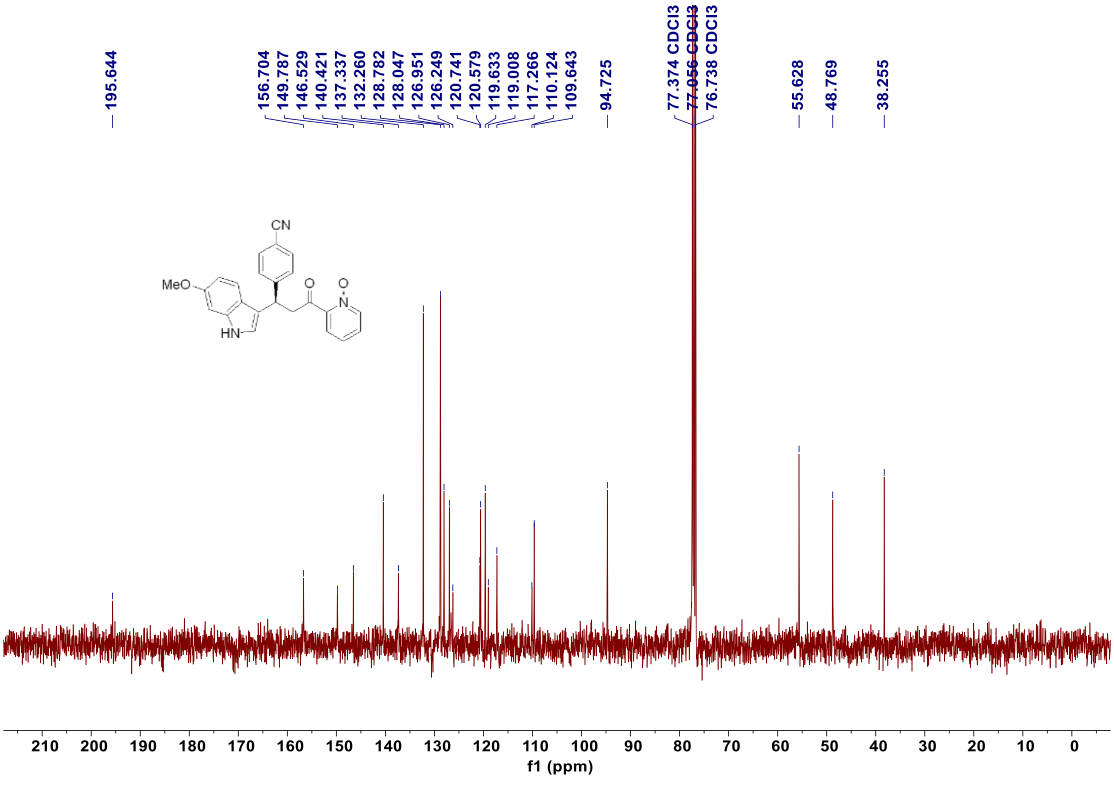

(*R*)-2-(3-(6-Methoxy-1*H*-indol-3-yl)-3-(4-(trifluoromethyl)phenyl)propanoyl)pyridine 1-oxide (3ag)

^{1}H NMR (400 MHz, $CDCl_3$)

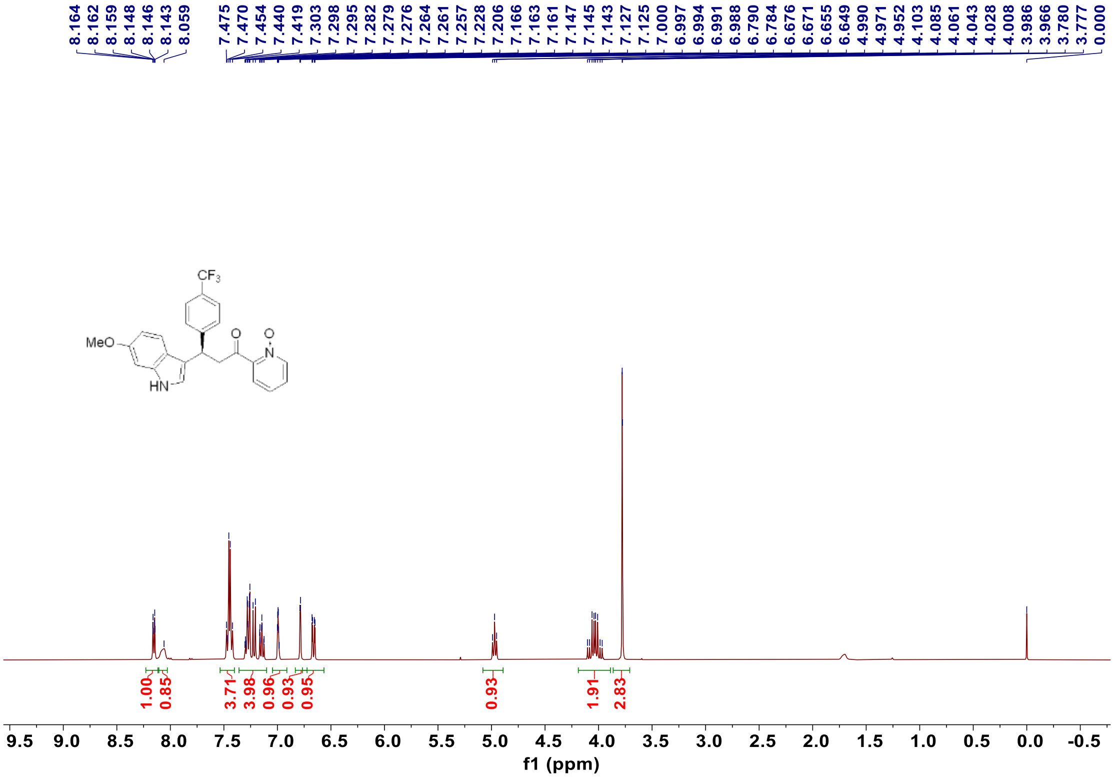

$^{13}C\{^{1}H\}$ NMR (101 MHz, $CDCl_3$)

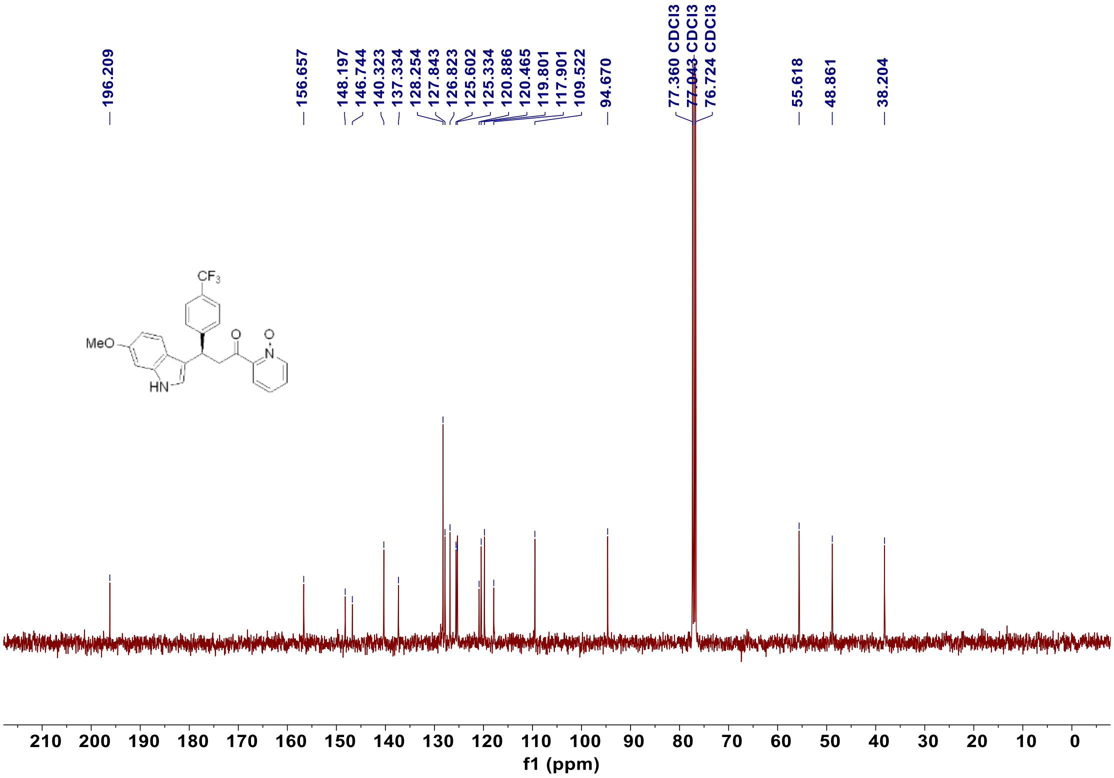

$^{19}F\{^{1}H\}$ NMR (376 MHz, $CDCl_3$)

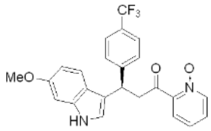

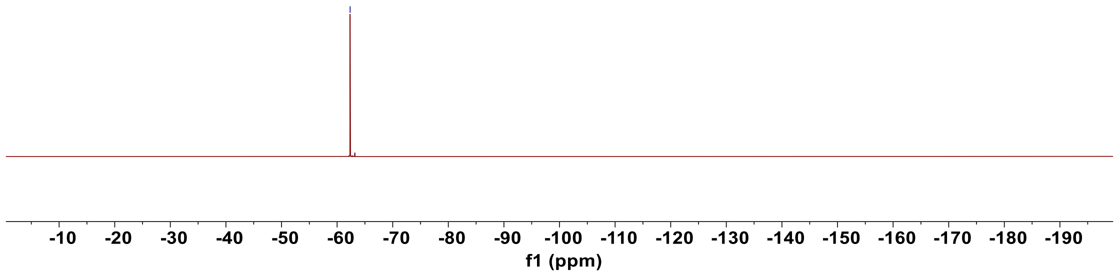

(*S*)-2-(3-(6-Methoxy-1*H*-indol-3-yl)-3-(2-methoxyphenyl)propanoyl)pyridine 1-oxide (3ah)

^{1}H NMR (400 MHz, $CDCl_3$)

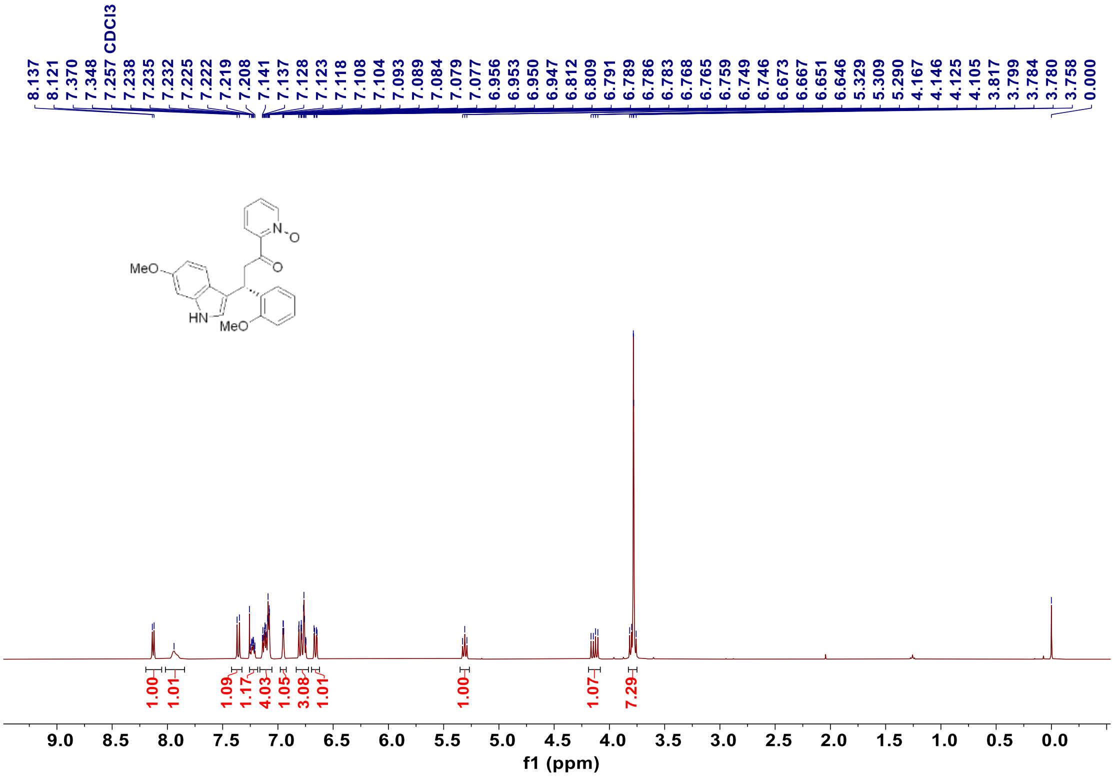

$^{13}C\{^{1}H\}$ NMR (101 MHz, $CDCl_3$)

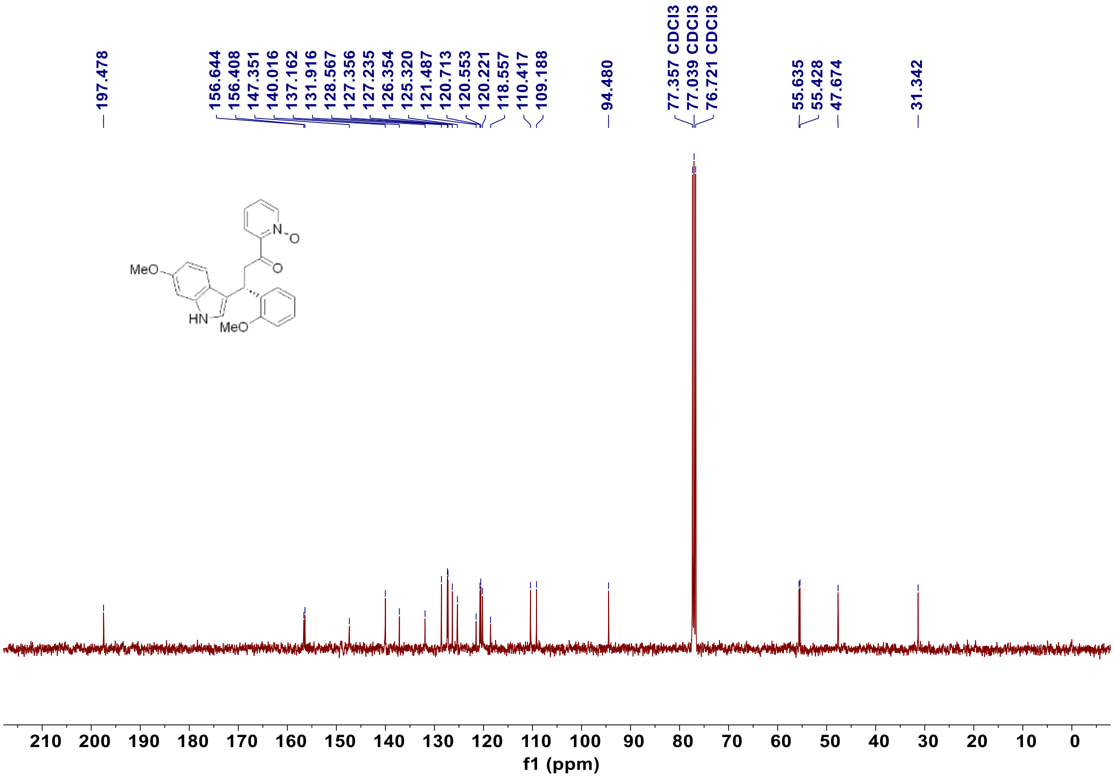

(*S*)-2-(3-(2-Fluorophenyl)-3-(6-methoxy-1H-indol-3-yl)propanoyl)pyridine 1-oxide (3ai)

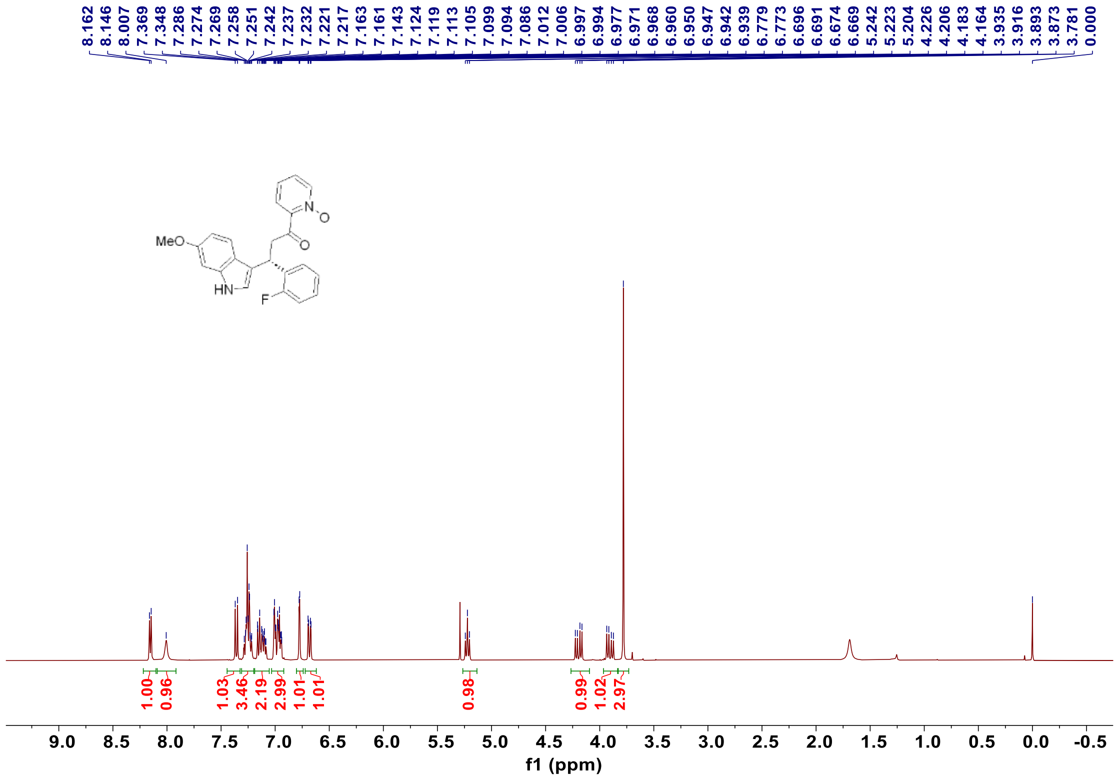

$^{13}C\{^1H\}$ NMR (101 MHz, $CDCl_3$)

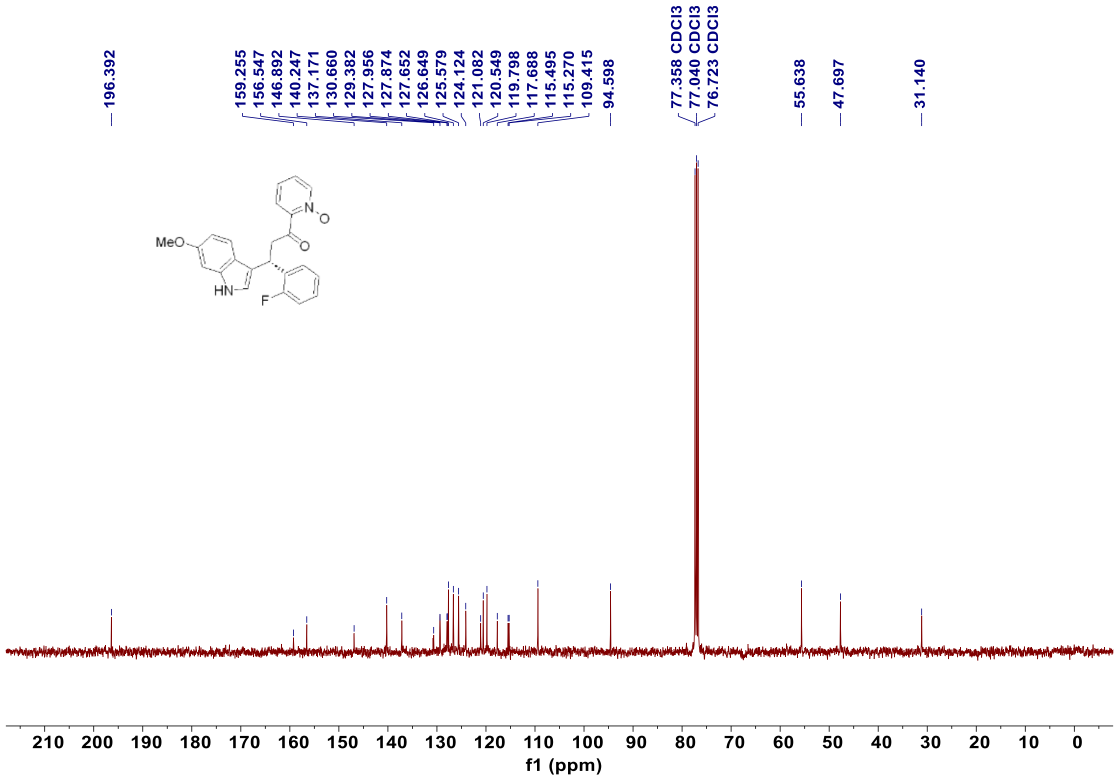

19{1H} NMR (376 MHz, $CDCl_3$)

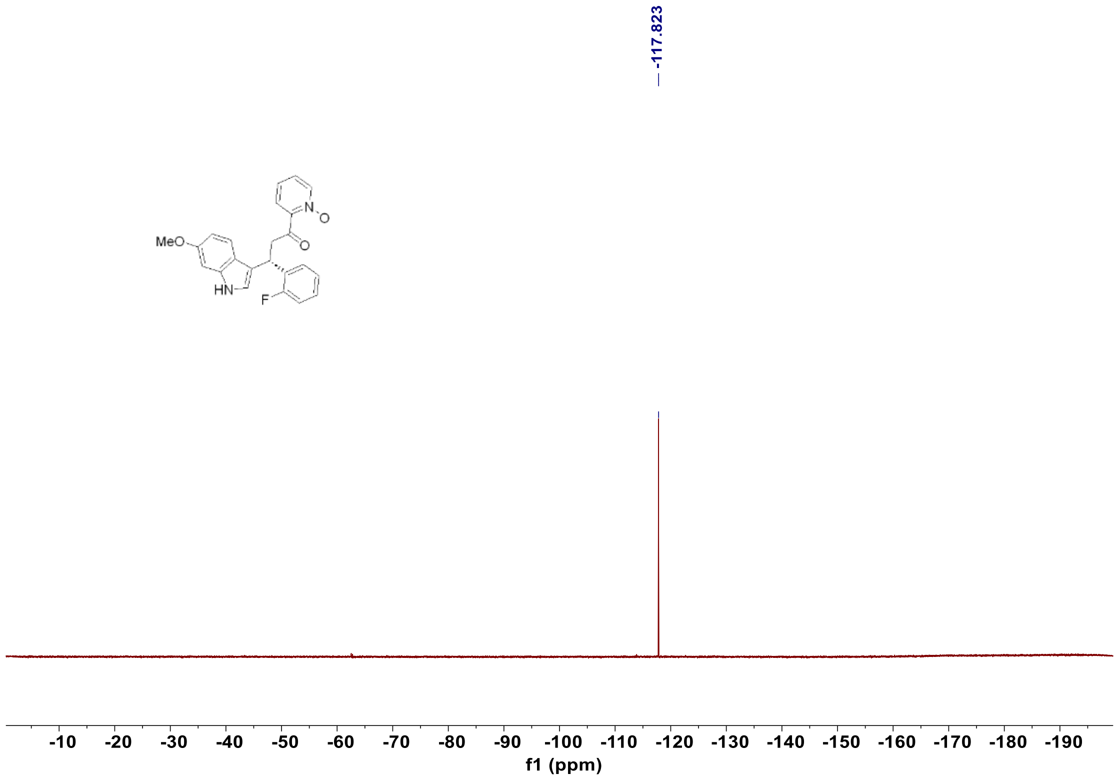

(*S*)-2-(3-(2-Chlorophenyl)-3-(6-methoxy-1*H*-indol-3-yl)propanoyl)pyridine 1-oxide (3aj)

^{1}H NMR (400 MHz, $CDCl_3$)

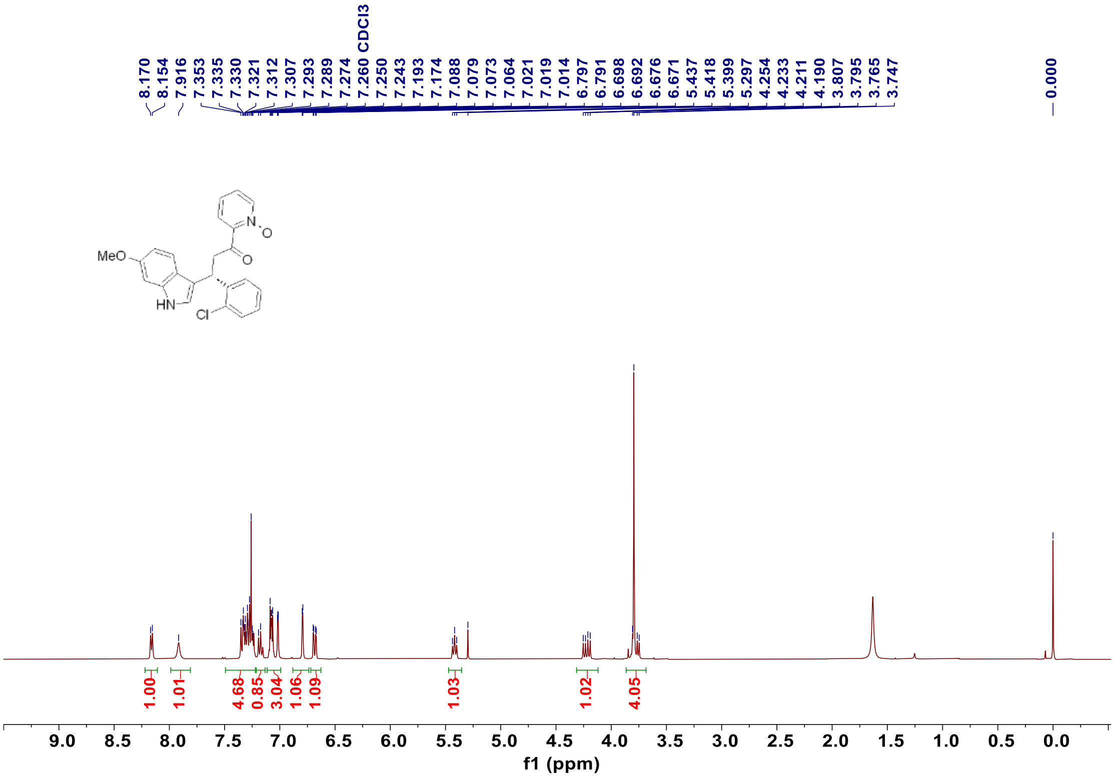

^{13}C{^{1}H} NMR (101 MHz, $CDCl_3$)

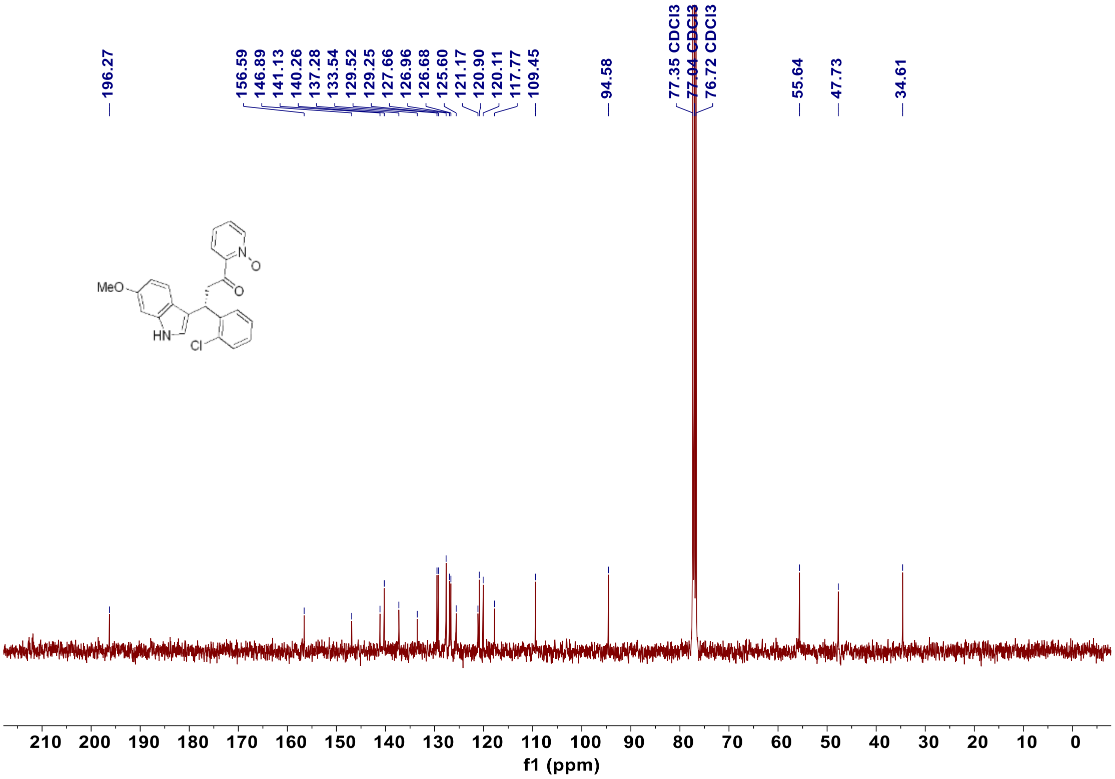

(*S*)-2-(3-(2-Bromophenyl)-3-(6-methoxy-1*H*-indol-3-yl)propanoyl)pyridine 1-oxide (3ak)

^{1}H NMR (400 MHz, $CDCl_3$)

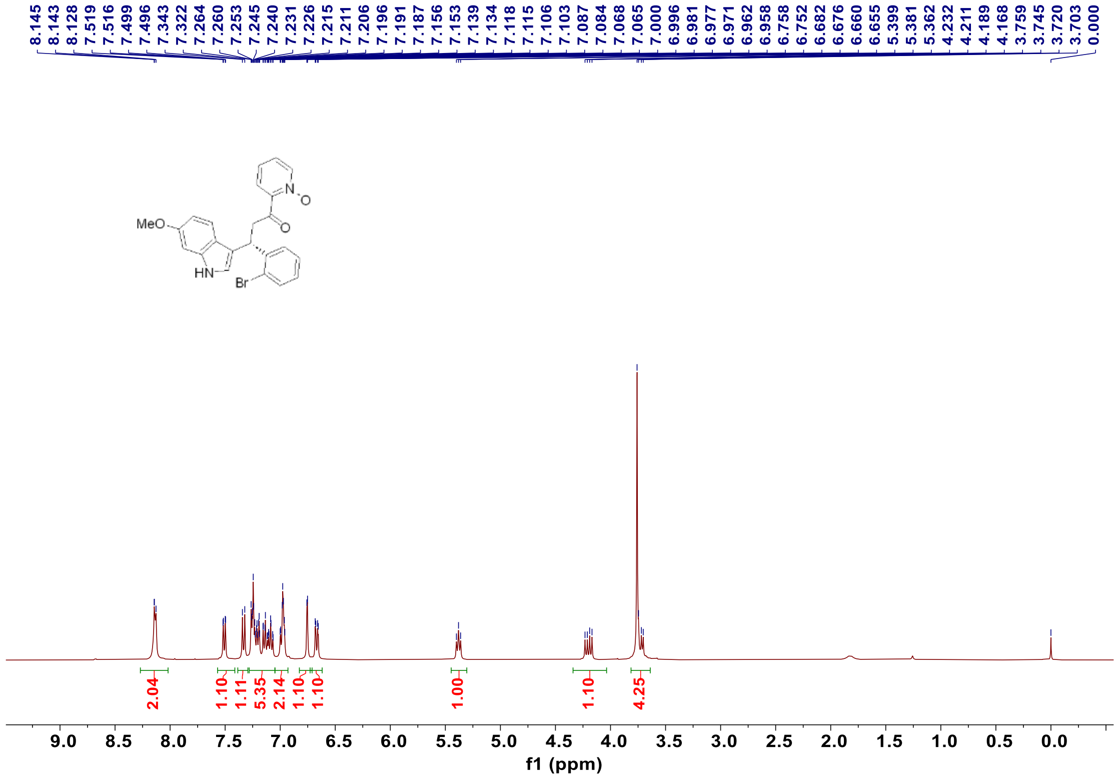

^{13}C NMR (101 MHz, **DMSO-*d*$_6$**)

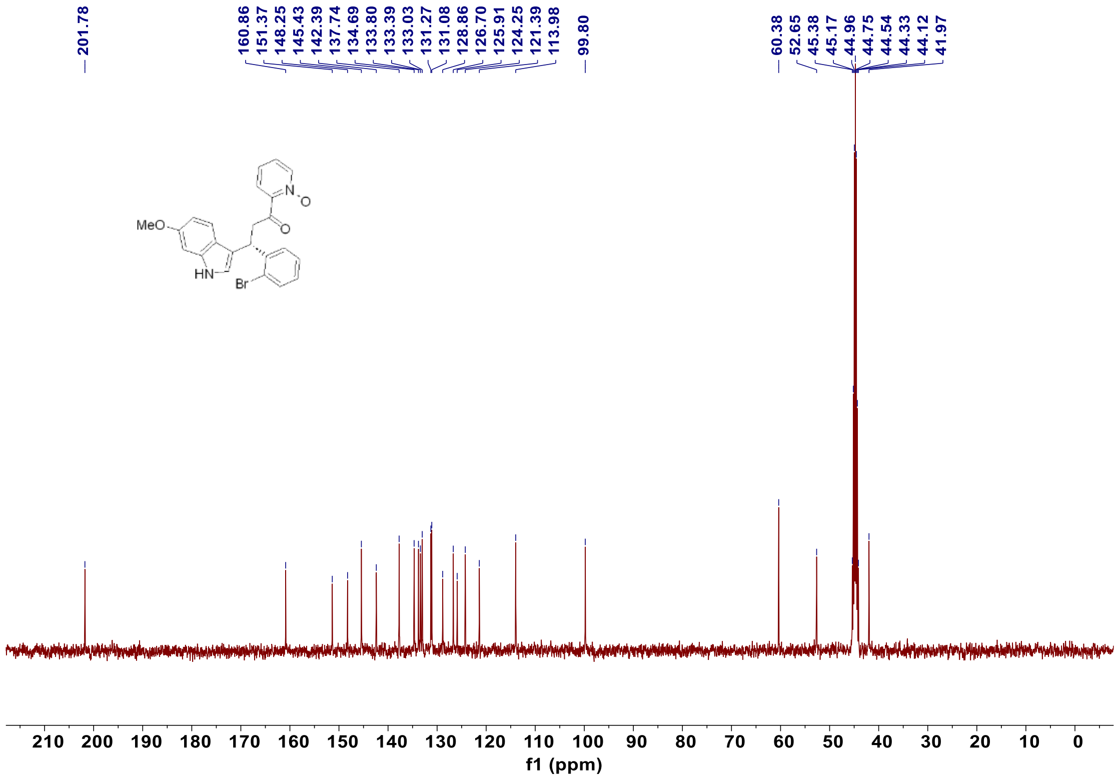

(*R*)-2-(3-(6-Methoxy-1*H*-indol-3-yl)-3-(naphthalen-2-yl)propanoyl)pyridine 1-oxide (3al)

^{1}H NMR (400 MHz, $CDCl_3$)

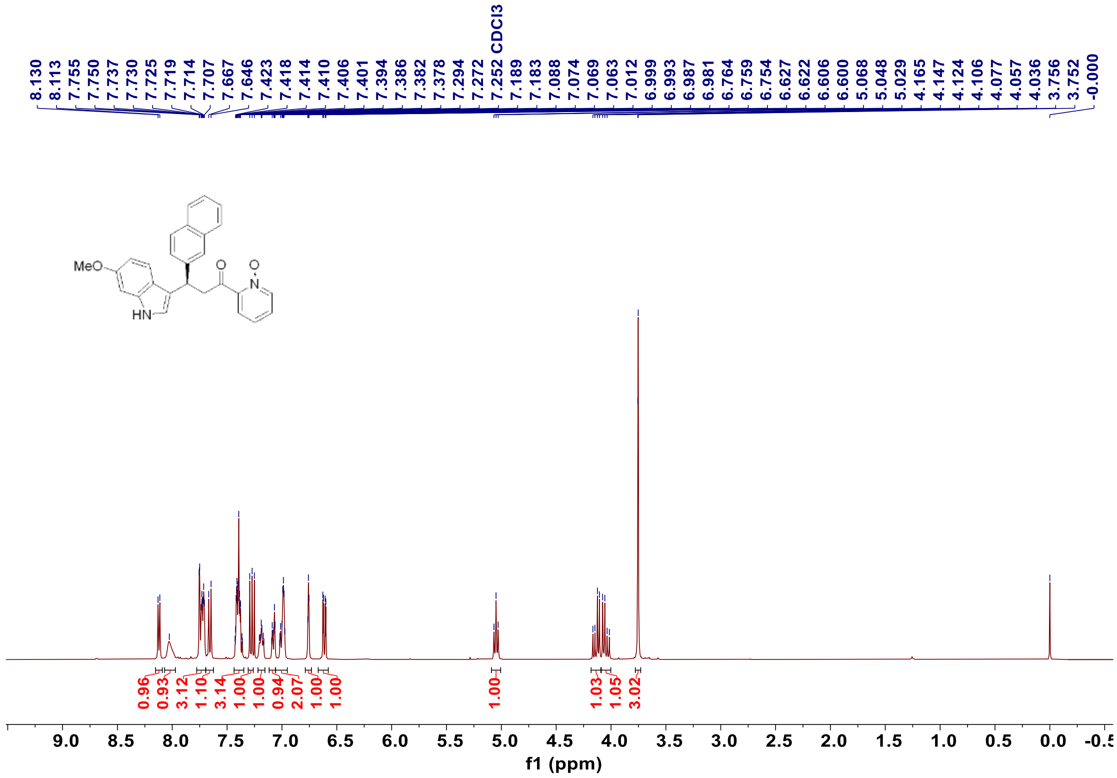

^{13}C{^{1}H} NMR (101 MHz, $CDCl_3$)

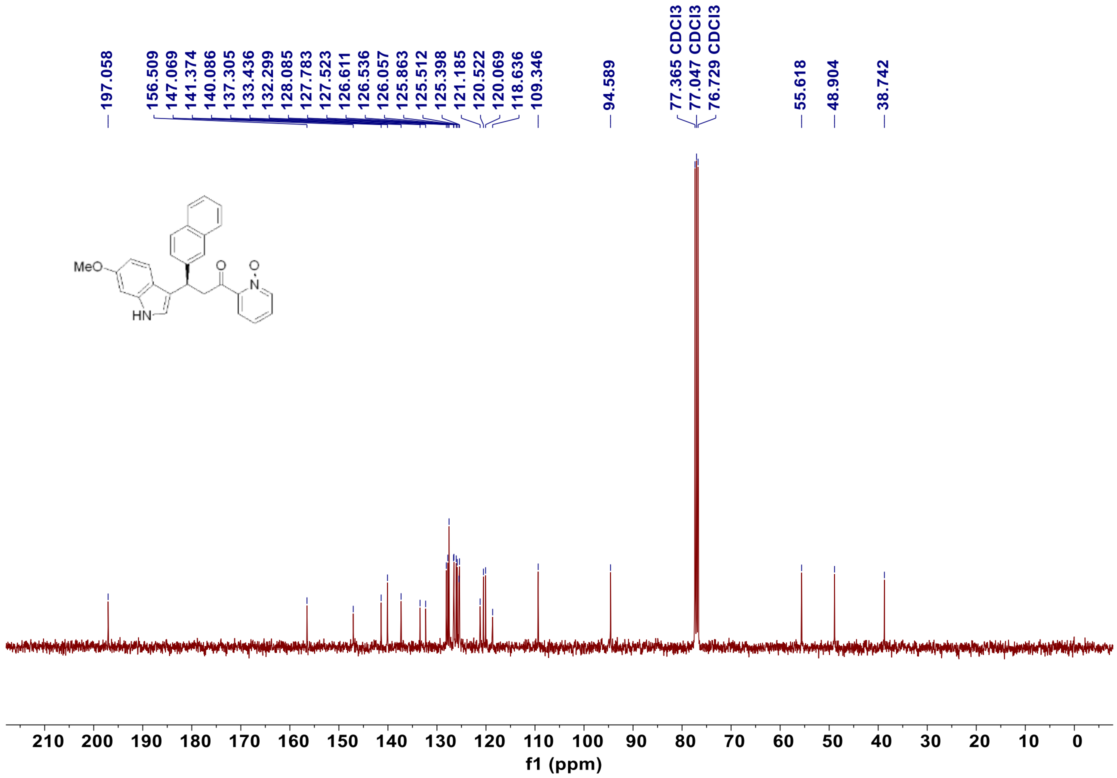

(*S*)-2-(3-(Furan-2-yl)-3-(6-methoxy-1*H*-indol-3-yl)propanoyl)pyridine 1-oxide (3am)

^{1}H NMR (400 MHz, $CDCl_3$)

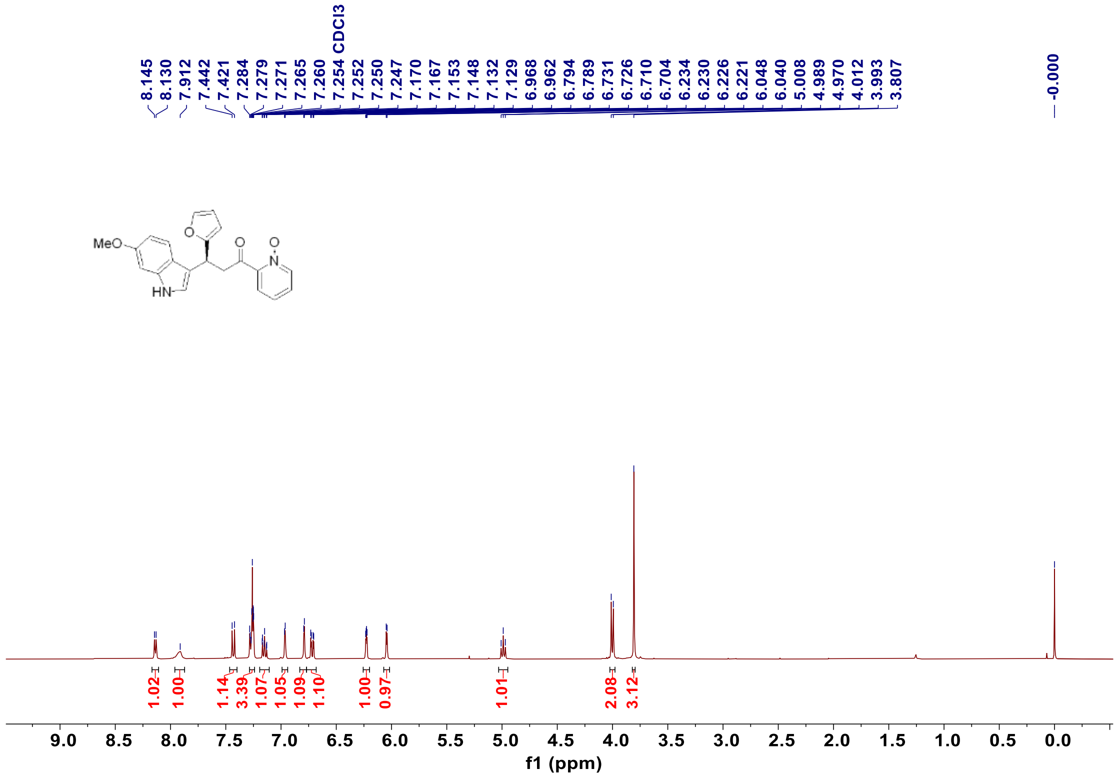

^{13}C{^{1}H} NMR (101 MHz, $CDCl_3$)

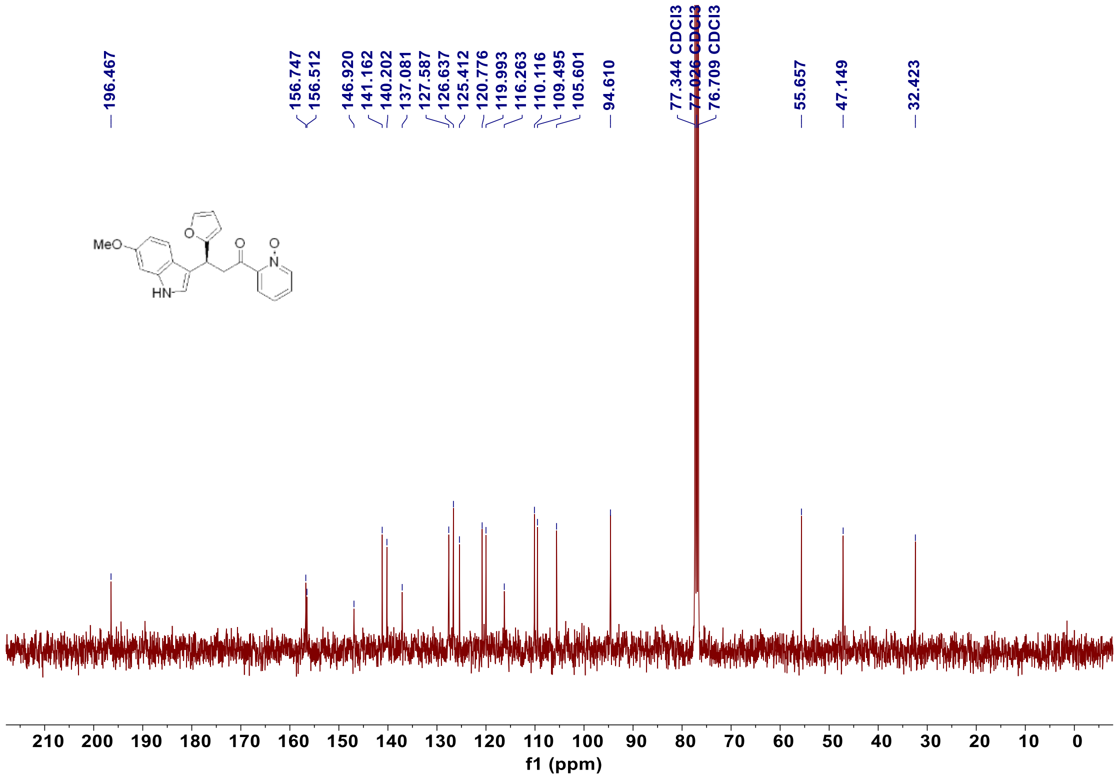

3. Copies of HPLC profiles of products 3

(*R*)-2-(3-(6-zmethoxy-1*H*-indol-3-yl)-3-phenylpropanoyl)pyridine 1-oxide (3aa)

ee 96%, HPLC analysis: chiralpak AD-H (*i*-PrOH/hexane = 40:60, *v*/*v*, 1.0 mL/min, 254 nm) major t_R = 15.57 min and minor t_S = 18.27 min.

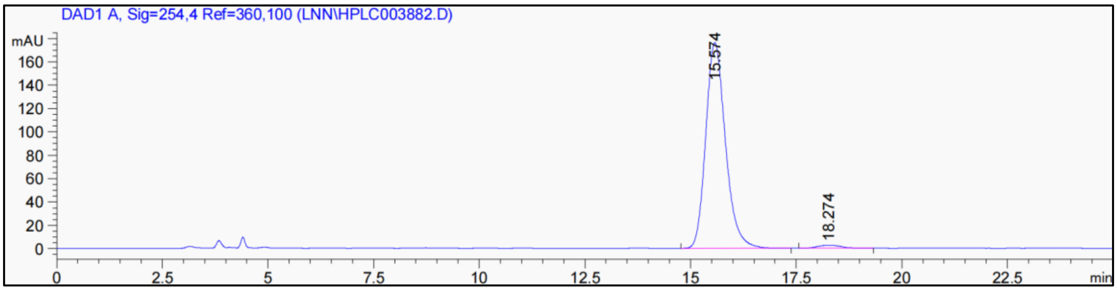

Peak	Ret. Time [min]	Type	Width [min]	Area [Mau*s]	Height [mAU]	Area %
1	15.574	BB	0.4987	5723.54492	176.34422	98.1780
2	18.274	BB	0.5622	106.22013	2.81519	1.8220

Racemic

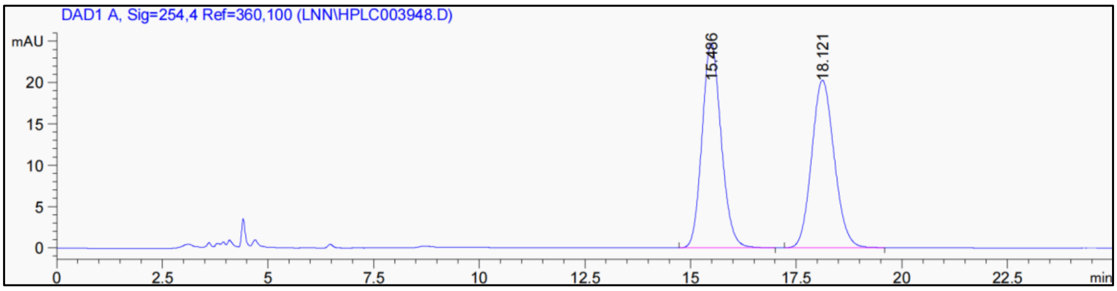

Peak	Ret. Time [min]	Type	Width [min]	Area [Mau*s]	Height [mAU]	Area %
1	15.486	BB	0.4872	780.43475	24.79493	50.9175
2	18.121	BB	0.5751	752.30939	20.26546	49.0825

(*R*)-2-(3-(6-Methyl-1*H*-indol-3-yl)-3-phenylpropanoyl)pyridine 1-oxide (3ba)

ee 94%, HPLC analysis: chiralpak AD-H (*i*-PrOH/hexane = 40:60, *v*/*v*, 1.0 mL/min, 230 nm) major t_R = 13.48 min and minor t_S = 16.79 min.

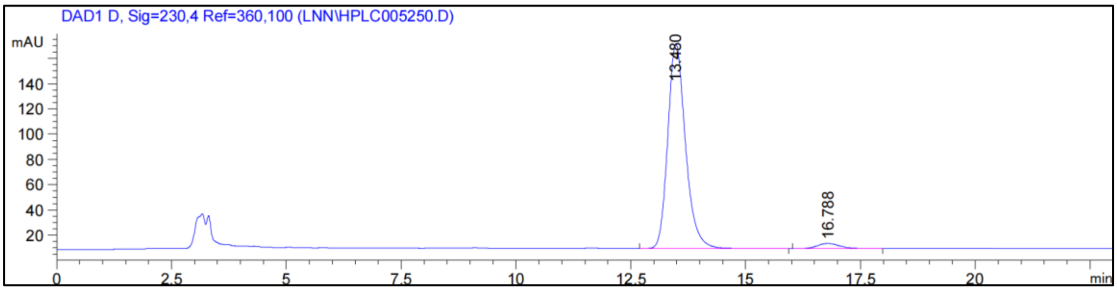

Peak	Ret. Time [min]	Type	Width [min]	Area [Mau*s]	Height [mAU]	Area %
1	13.480	BB	0.4082	4339.27490	162.14465	96.9265
2	16.788	BB	0.5095	137.59566	4.14213	3.0735

Racemic

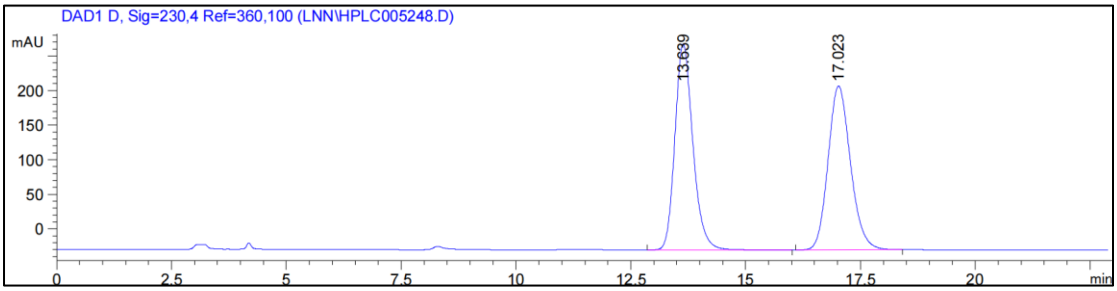

Peak	Ret. Time [min]	Type	Width [min]	Area [Mau*s]	Height [mAU]	Area %
1	13.639	BB	0.4161	8070.85986	297.83777	50.1112
2	17.023	BB	0.5239	8035.02734	236.78473	49.8888

(*R*)-2-(3-(6-Bromo-1*H*-indol-3-yl)-3-phenylpropanoyl)pyridine 1-oxide (3ca)

ee 98%, HPLC analysis: chiralpak AD-H (*i*-PrOH/hexane = 30:70, *v*/*v*, 1.0 mL/min, 254 nm) major t_R = 10.97 min and minor t_S = 12.26 min.

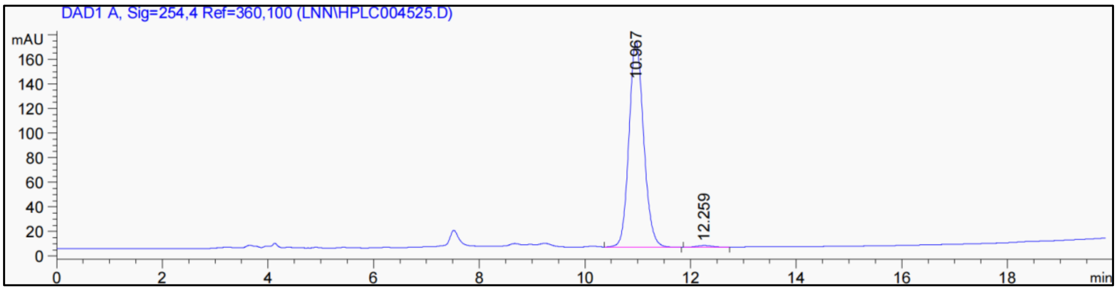

Peak	Ret. Time [min]	Type	Width [min]	Area [Mau*s]	Height [mAU]	Area %
1	10.967	BB	0.2987	3230.31592	167.71217	99.0725
2	12.259	BB	0.3216	30.24080	1.48477	0.9275

Racemic

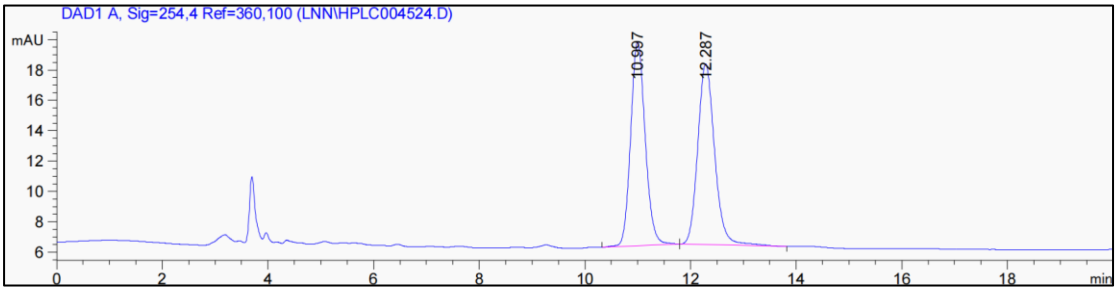

Peak	Ret. Time [min]	Type	Width [min]	Area [Mau*s]	Height [mAU]	Area %
1	10.997	BB	0.2955	257.59521	13.44635	49.2834
2	12.287	BB	0.3418	265.08612	11.90371	50.7166

(*R*)-2-(3-(6-Fluoro-1*H*-indol-3-yl)-3-phenylpropanoyl)pyridine 1-oxide (3da)

ee 99%, HPLC analysis: chiralpak AD-H (*i*-PrOH/hexane = 40:60, *v*/*v*, 1.0 mL/min, 254 nm) major t_R = 9.42 min and minor t_S = 10.38 min

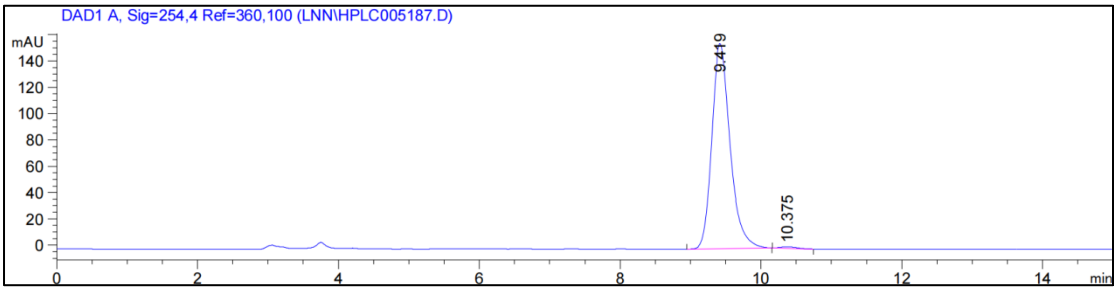

Peak	Ret. Time [min]	Type	Width [min]	Area [Mau*s]	Height [mAU]	Area%
1	9.419	BB	0.2734	2797.00000	155.86798	99.3116
2	10.375	BB	0.2559	19.38726	1.23007	0.6884

Racemic

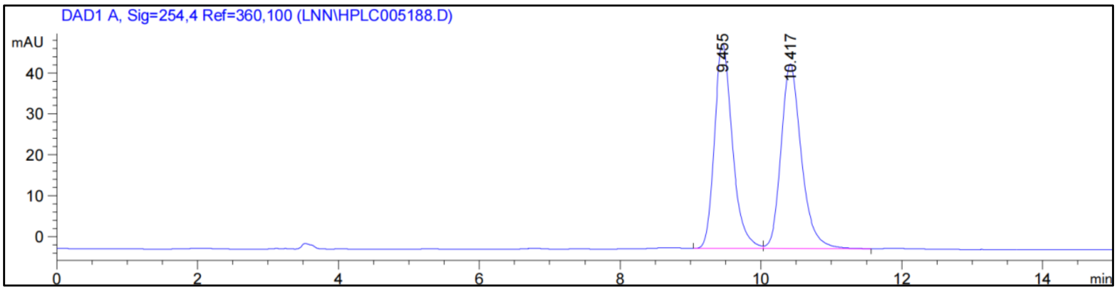

Peak	Ret. Time [min]	Type	Width [min]	Area [Mau*s]	Height [mAU]	Area %
1	9.455	BV	0.2711	889.22516	50.09607	49.5481
2	10.417	VB	0.3059	905.44568	45.17223	50.4519

(*R*)-2-(3-(6-(Methoxycarbonyl)-1*H*-indol-3-yl)-3-phenylpropanoyl)pyridine 1-oxide (3ea)

ee 23%, HPLC analysis: chiralpak AD-H (*i*-PrOH/hexane = 30:70, *v*/*v*, 1.0 mL/min, 230 nm) major t_R = 39.17 min and minor t_S = 54.22 min

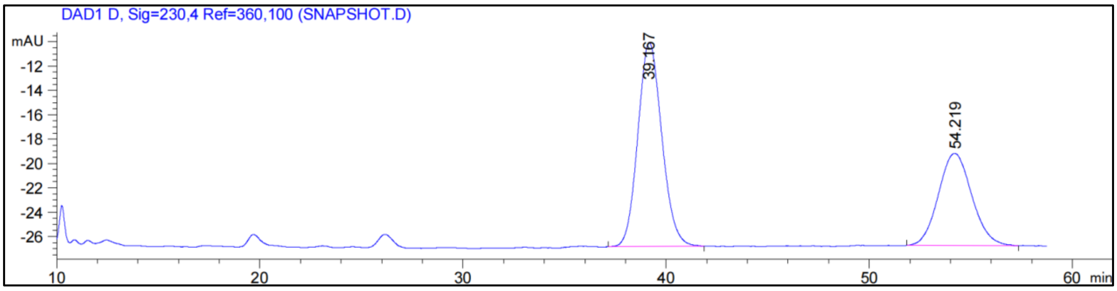

Peak	Ret. Time [min]	Type	Width [min]	Area [Mau*s]	Height [mAU]	Area %
1	39.167	BB	1.2490	1375.16516	16.76443	61.3891
2	54.219	BB	1.4060	864.91339	7.57571	38.6109

Racemic

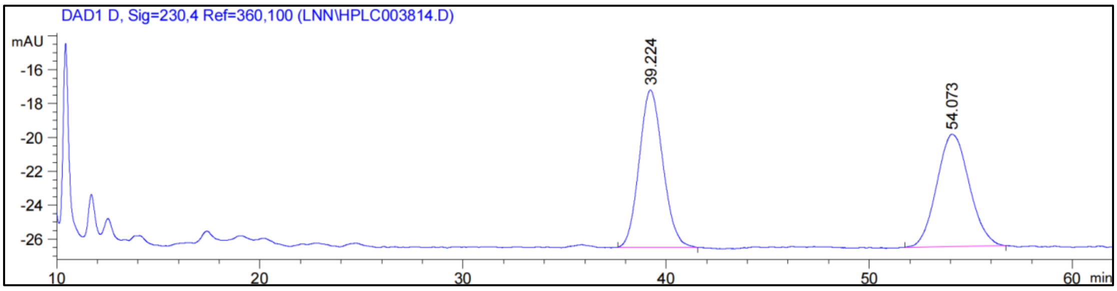

Peak	Ret. Time [min]	Type	Width [min]	Area [Mau*s]	Height [mAU]	Area %
1	39.224	BB	1.1527	750.88898	9.29255	50.4107
2	54.073	BB	1.3636	738.65265	6.64110	49.5893

(*R*)-2-(3-(5-Methoxy-1*H*-indol-3-yl)-3-phenylpropanoyl)pyridine 1-oxide (3fa)

ee 88%, HPLC analysis: chiralpak AD-H (*i*-PrOH/hexane = 30:70, *v*/*v*, 1.0 mL/min, 254 nm) major t_R = 16.29 min and minor t_S = 19.35 min

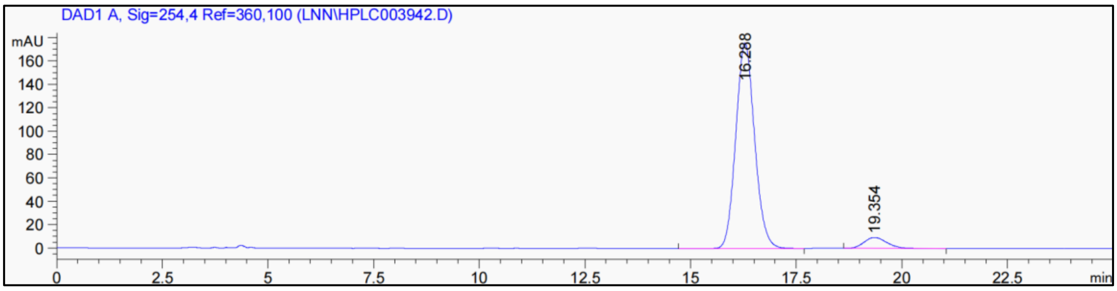

Peak	Ret. Time [min]	Type	Width [min]	Area [Mau*s]	Height [mAU]	Area %
1	16.288	BB	0.4798	5455.79053	175.94279	93.8156
2	19.354	BB	0.5846	359.65143	9.52335	6.1844

Racemic

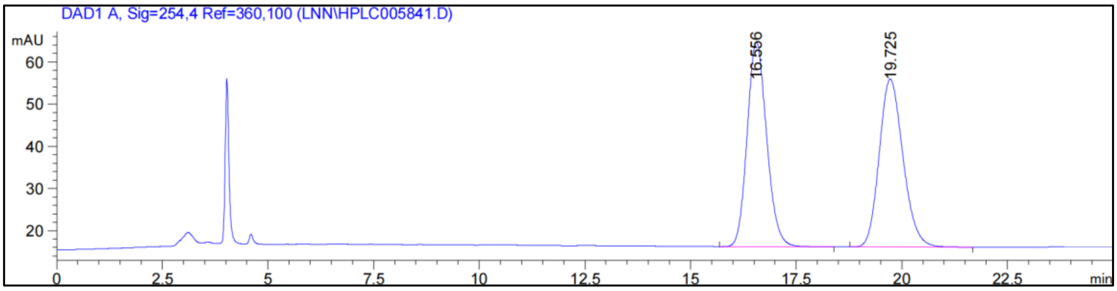

Peak	Ret. Time [min]	Type	Width [min]	Area [Mau*s]	Height [mAU]	Area %
1	16.556	BB	0.4988	1560.90015	48.58812	49.9522
2	19.725	BB	0.6082	1563.88660	39.81733	50.0478

(*R*)-2-(3-(5-(Benzyloxy)-1*H*-indol-3-yl)-3-phenylpropanoyl)pyridine 1-oxide (3ga)

ee 78%, HPLC analysis: chiralpak AD-H (*i*-PrOH/hexane = 40:60, *v*/*v*, 1.0 mL/min, 254 nm) major t_R = 24.97 min and minor t_S = 29.80 min

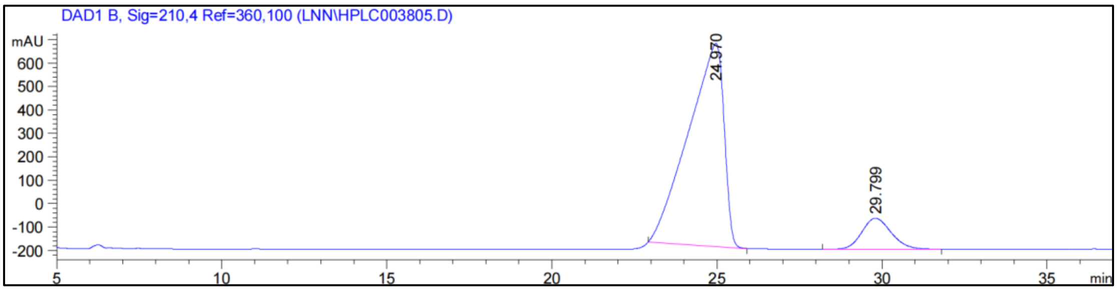

Peak	Ret. Time [min]	Type	Width [min]	Area [Mau*s]	Height [mAU]	Area %
1	24.970	BB	1.0315	6.49977e4	866.17889	88.8817
2	29.799	BB	0.9511	8130.60986	131.91663	11.1183

Racemic

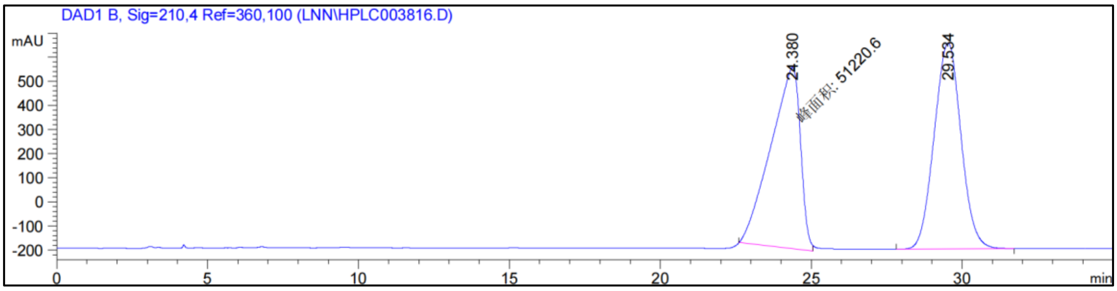

Peak	Ret. Time [min]	Type	Width [min]	Area [Mau*s]	Height [mAU]	Area %
1	24.380	MM	1.1359	5.12206e4	751.51801	49.2402
2	29.534	BB	0.9656	5.28014e4	853.69891	50.7598

(*R*)-2-(3-(5-Chloro-1*H*-indol-3-yl)-3-phenylpropanoyl)pyridine 1-oxide (3ha)

ee 98%, HPLC analysis: chiralpak AD-H (*i*-PrOH/hexane = 40:60, *v*/*v*, 1.0 mL/min, 230 nm) major t_R = 10.06 min and minor t_S = 12.04 min

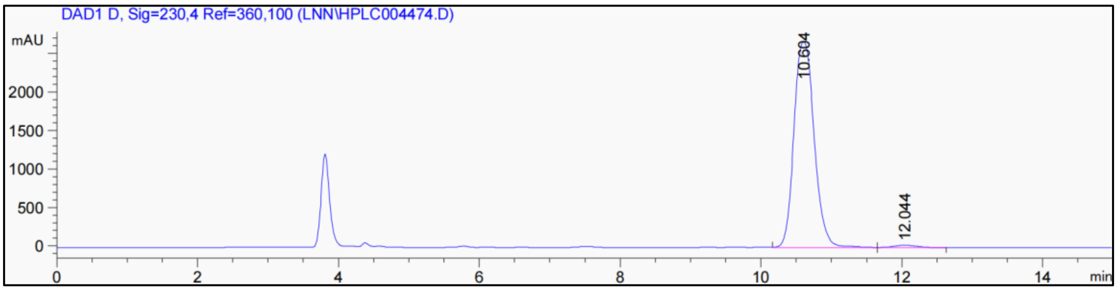

Peak	Ret. Time [min]	Type	Width [min]	Area [Mau*s]	Height [mAU]	Area %
1	10.604	VV	0.3125	5.33340e4	2675.79590	98.7233
2	12.044	VB	0.3277	689.73297	32.22149	1.2767

Racemic

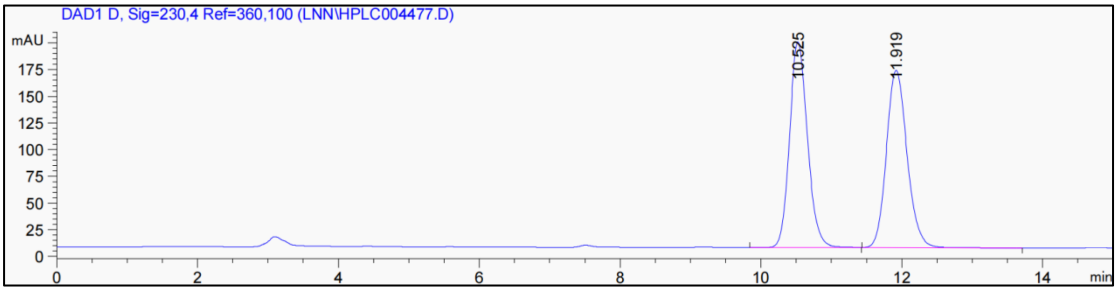

Peak	Ret. Time [min]	Type	Width [min]	Area [Mau*s]	Height [mAU]	Area %
1	10.525	BB	0.2721	3405.02832	192.72142	50.1990
2	11.919	BB	0.3133	3378.02661	166.07657	49.8010

(*R*)-2-(3-(5-Fluoro-1*H*-indol-3-yl)-3-phenylpropanoyl)pyridine 1-oxide (3ia)

ee 93%, HPLC analysis: chiralpak AD-H (*i*-PrOH/hexane = 40:60, *v*/*v*, 1.0 mL/min, 254 nm) major t_R = 8.09 min and minor t_S = 8.84 min

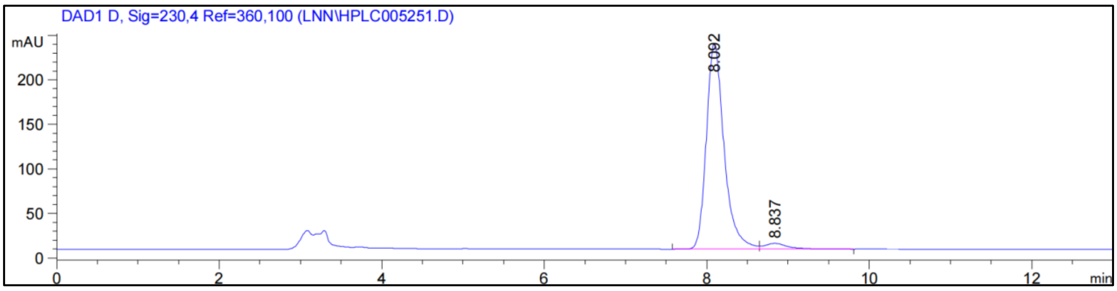

Peak	Ret. Time [min]	Type	Width [min]	Area [Mau*s]	Height [mAU]	Area %
1	8.092	BV	0.2291	3506.81201	230.95436	96.3337
2	8.837	VB	0.2939	133.46158	6.54374	3.6663

Racemic

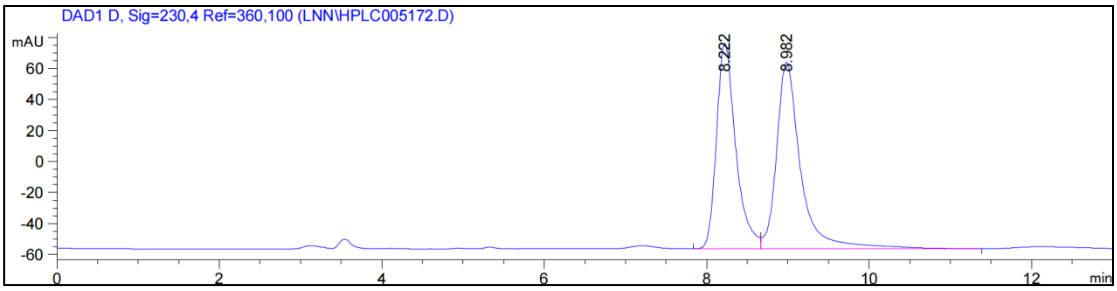

Peak	Ret. Time [min]	Type	Width [min]	Area [Mau*s]	Height [mAU]	Area %
1	8.222	BV	0.2480	2181.30933	132.56204	46.6029
2	8.982	VB	0.3042	2499.32251	120.37980	53.3971

(*R*)-2-(3-(4-Methoxy-1*H*-indol-3-yl)-3-phenylpropanoyl)pyridine 1-oxide (3ja)

ee 57%, HPLC analysis: chiralpak AD-H (*i*-PrOH/hexane = 40:60, *v*/*v*, 1.0 mL/min, 254 nm) major t_R = 8.46 min and minor t_S = 9.19 min

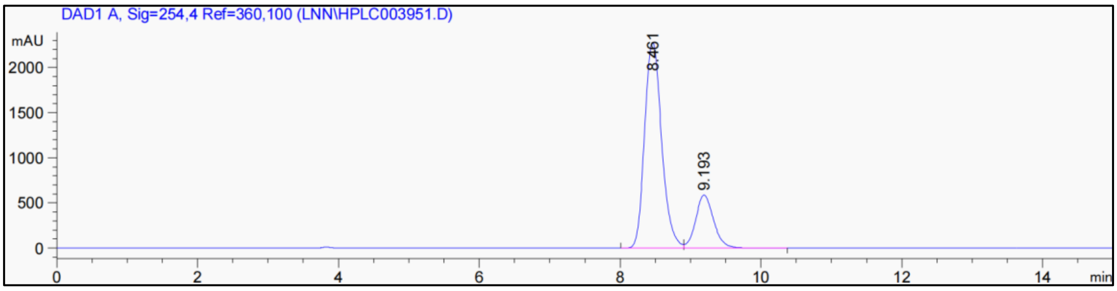

Peak	Ret. Time [min]	Type	Width [min]	Area [Mau*s]	Height [mAU]	Area %
1	8.461	BV	0.2597	3.82553e4	2280.61743	78.6854
2	9.193	VB	0.2703	1.03627e4	585.96307	21.3146

Racemic

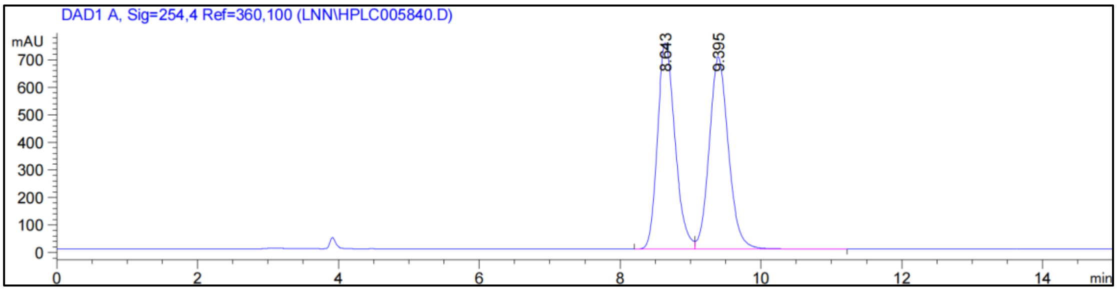

Peak	Ret. Time [min]	Type	Width [min]	Area [Mau*s]	Height [mAU]	Area %
1	8.643	BV	0.2654	1.29176e4	748.29236	49.4612
2	9.395	VB	0.2906	1.31990e4	698.14453	50.5388

(*R*)-2-(3-(1-Methyl-1*H*-indol-3-yl)-3-phenylpropanoyl)pyridine 1-oxide (3ka)

ee 38%, HPLC analysis: chiralpak OD-H (*i*-PrOH/hexane = 30:70, *v*/*v*, 1.0 mL/min, 254 nm) major t_R = 18.26 min and minor t_S = 24.19 min

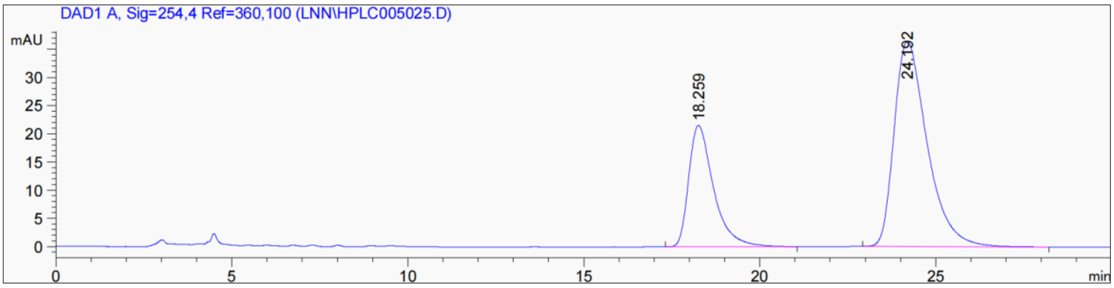

Peak	Ret. Time [min]	Type	Width [min]	Area [Mau*s]	Height [mAU]	Area %
1	18.259	BB	0.7439	1055.57117	21.53487	30.8427
2	24.192	BB	0.9886	2366.85864	36.39857	69.1573

Racemic

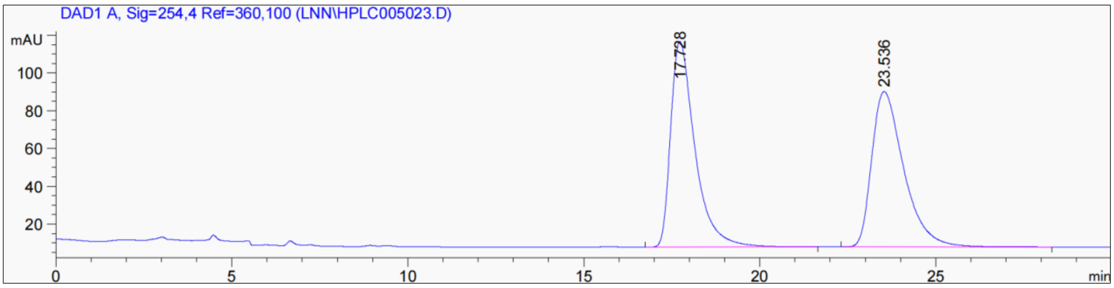

Peak	Ret. Time [min]	Type	Width [min]	Area [Mau*s]	Height [mAU]	Area %
1	17.728	BB	0.7259	5227.85986	108.92572	50.0788
2	23.536	BB	0.9649	5211.40479	82.28493	49.9212

(*R*)-2-(3-(1*H*-Indol-3-yl)-3-phenylpropanoyl)pyridine 1-oxide (3ma)

ee 51%, HPLC analysis: chiralpak AD-H (*i*-PrOH/hexane = 30:70, *v*/*v*, 1.0 mL/min, 210 nm) major t_R = 10.59 min and minor t_S = 12.40 min

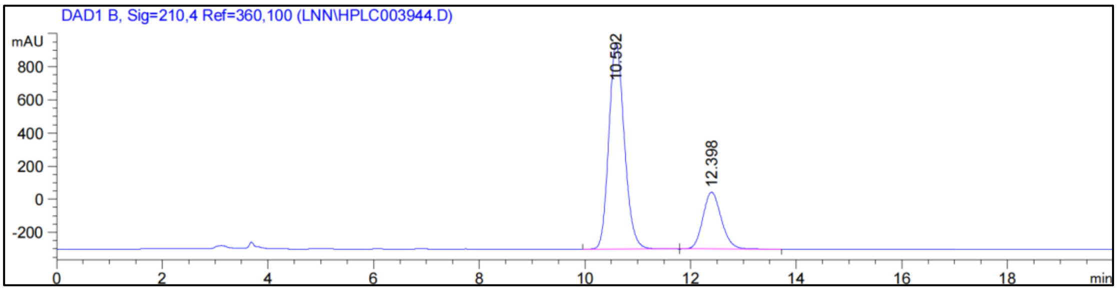

Peak	Ret. Time [min]	Type	Width [min]	Area [Mau*s]	Height [mAU]	Area %
1	10.592	BB	0.3138	2.53017e4	1241.30432	75.2421
2	12.398	BB	0.3747	8325.34668	343.53061	24.7579

Racemic

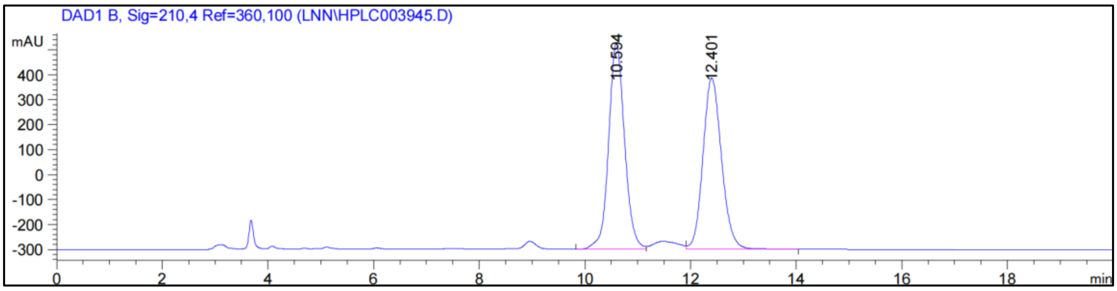

Peak	Ret. Time [min]	Type	Width [min]	Area [Mau*s]	Height [mAU]	Area %
1	10.594	BV	0.3232	1.73355e4	824.82251	50.7734
2	12.401	VB	0.3777	1.68074e4	686.39020	49.2266

(*R*)-2-(3-(6-Methoxy-1*H*-indol-3-yl)-3-(4-methoxyphenyl)propanoyl)pyridine (3ab) 1-oxide

ee 50%, HPLC analysis: chiralpak AS-H (*i*-PrOH/hexane = 30:70, *v*/*v*, 1.0 mL/min, 210 nm) major t_R = 18.01 min and minor t_R = 22.06 min

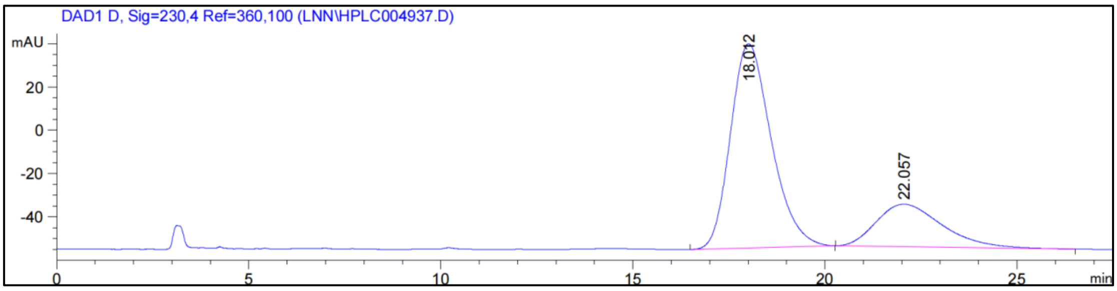

Peak	Ret. Time [min]	Type	Width [min]	Area [Mau*s]	Height [mAU]	Area %
1	18.012	BB	1.1468	6953.40723	94.24373	74.8004
2	22.057	BBA	1.7105	2342.54053	19.59784	25.1996

Racemic

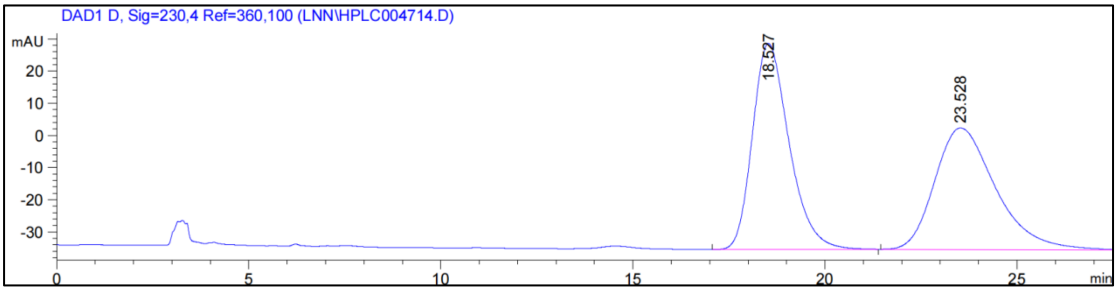

Peak	Ret. Time [min]	Type	Width [min]	Area [Mau*s]	Height [mAU]	Area %
1	18.527	BB	1.0173	4248.32617	64.09306	50.2997
2	23.528	BB	1.6404	4197.70410	37.83473	49.7003

(*R*)-2-(3-(4-Fluorophenyl)-3-(6-methoxy-1*H*-indol-3-yl)propanoyl)pyridine 1-oxide (3ac)

ee 73%, HPLC analysis: chiralpak OD-H (*i*-PrOH/hexane = 30:70, *v*/*v*, 1.0 mL/min, 254 nm) major t_R = 18.26 min and minor t_S = 24.19 min

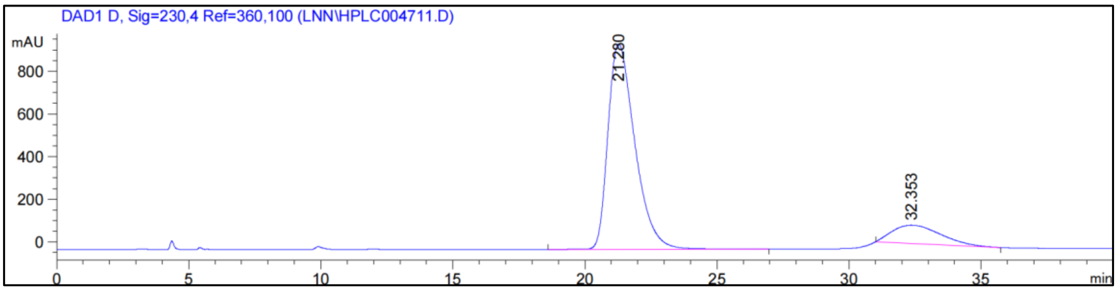

Peak	Ret. Time [min]	Type	Width [min]	Area [Mau*s]	Height [mAU]	Area %
1	21.280	BB	1.0864	6.80745e4	963.34058	86.3242
2	32.353	BB	1.8152	1.07846e4	84.71770	13.6758

Racemic

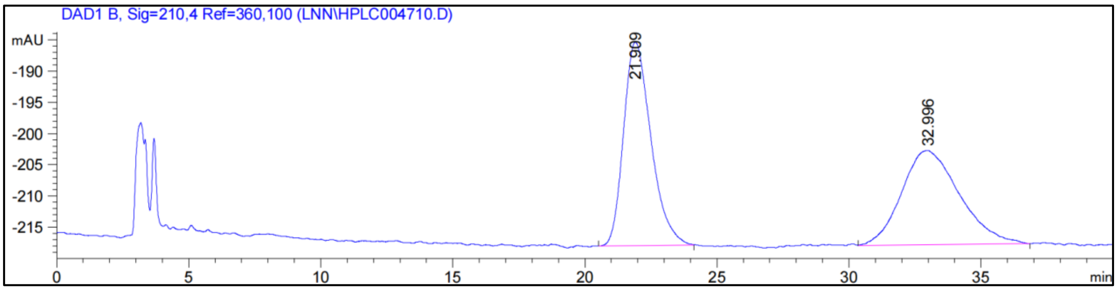

Peak	Ret. Time [min]	Type	Width [min]	Area [Mau*s]	Height [mAU]	Area %
1	21.909	BB	1.0664	2347.30444	32.58762	50.5092
2	32.996	BB	1.8083	2299.97974	15.10594	49.4908

(*R*)-2-(3-(4-Chlorophenyl)-3-(6-methoxy-1*H*-indol-3-yl)propanoyl)pyridine 1-oxide (3ad)

ee 67%, HPLC analysis: chiralpak AS-H (*i*-PrOH/hexane = 30:70, *v*/*v*, 1.0 mL/min, 210 nm)

major t_R = 21.68 min and minor t_R = 29.63 min

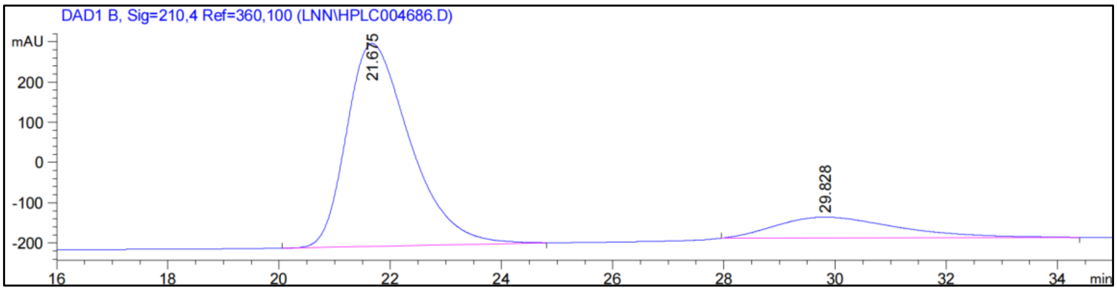

Peak	Ret. Time [min]	Type	Width [min]	Area [Mau*s]	Height [mAU]	Area %
1	21.675	BB	1.2126	3.99431e4	503.98758	83.4558
2	29.828	BB	2.0629	7918.29297	51.63958	16.5442

Racemic

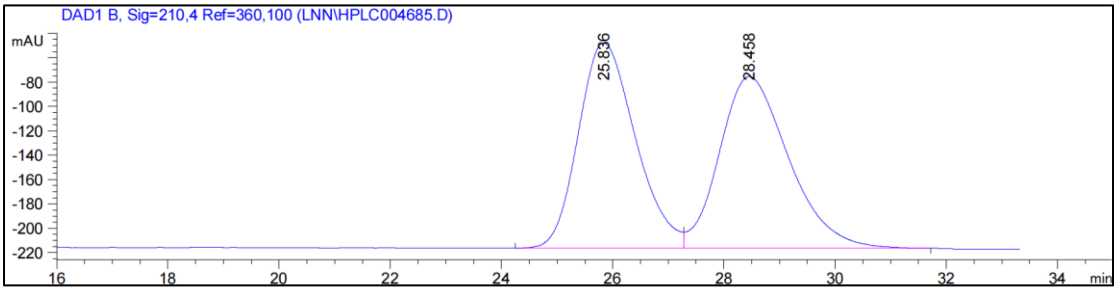

Peak	Ret. Time [min]	Type	Width [min]	Area [Mau*s]	Height [mAU]	Area %
1	25.836	BV	1.1052	1.19970e4	168.38919	49.6002
2	28.458	VB	1.3352	1.21904e4	140.92625	50.3998

(*R*)-2-(3-(4-Bromophenyl)-3-(6-methoxy-1H-indol-3-yl)propanoyl)pyridine 1-oxide (3ae)

ee 63%,HPLC analysis: chiralpak AS-H (*i*-PrOH/hexane = 40:60, *v*/*v*, 1.0 mL/min, 254 nm) major t_R = 14.18 min and minor t_S = 17.78 min

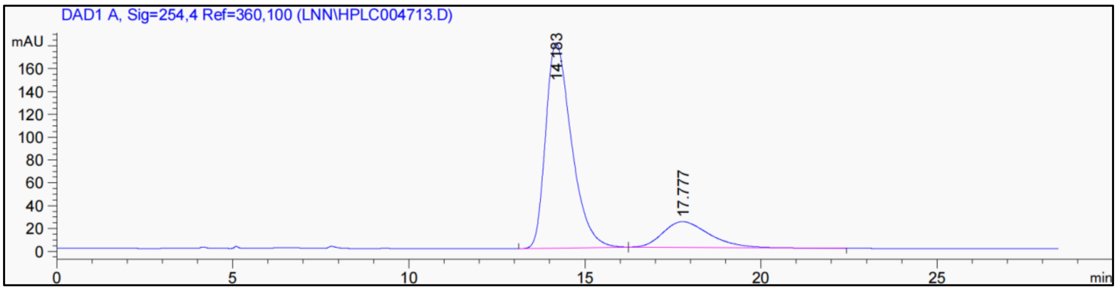

Peak	Ret. Time [min]	Type	Width [min]	Area [Mau*s]	Height [mAU]	Area %
1	14.183	BB	0.7821	9153.74609	179.76875	81.6097
2	17.777	BB	1.3804	2062.74072	22.48306	18.3903

Racemic

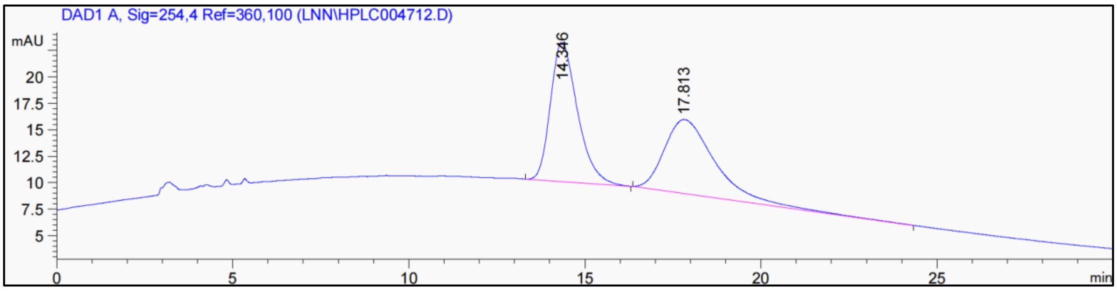

Peak	Ret. Time [min]	Type	Width [min]	Area [Mau*s]	Height [mAU]	Area %
1	14.346	BB	0.8153	701.09381	13.12321	49.8905
2	17.813	BBA	1.2732	704.17072	6.97859	50.1095

(*R*)-2-(3-(4-Cyanophenyl)-3-(6-methoxy-1*H*-indol-3-yl)propanoyl)pyridine 1-oxide (3af)

ee 71%, HPLC analysis: chiralpak AS-H (*i*-PrOH/hexane = 30:70, *v*/*v*, 1.0 mL/min, 214 nm) major t_R = 30.13 min and minor t_S = 35.94 min

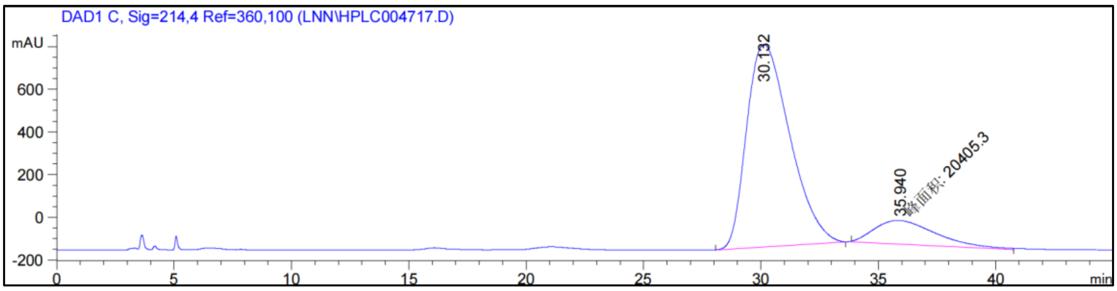

Peak	Ret. Time [min]	Type	Width [min]	Area [Mau*s]	Height [mAU]	Area %
1	30.132	BB	1.9490	1.21145e5	949.22058	85.5845
2	35.940	MM	3.0624	2.04053e4	111.05406	14.4155

Racemic

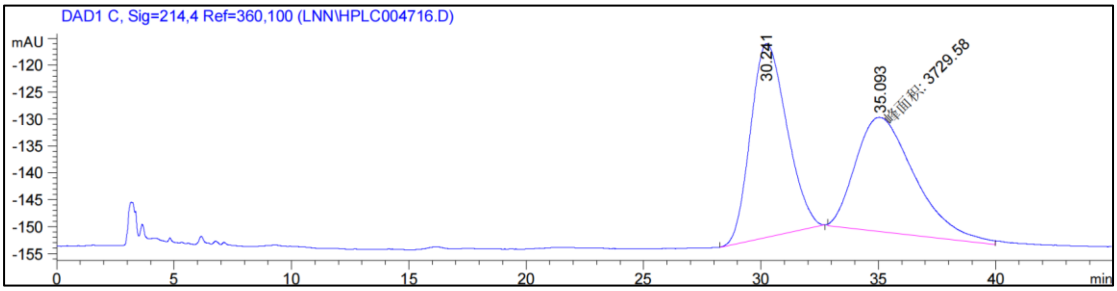

Peak	Ret. Time [min]	Type	Width [min]	Area [Mau*s]	Height [mAU]	Area %
1	30.241	BB	1.6300	3861.19067	35.97911	50.8669
2	35.093	MM	2.9255	3729.57983	21.24754	49.1331

(*R*)-2-(3-(6-Methoxy-1*H*-indol-3-yl)-3-(4-(trifluoromethyl)phenyl)propanoyl)pyridine 1-oxide (3ag)

ee 77%, HPLC analysis: chiralpak AD-H (*i*-PrOH/hexane = 40:60, *v*/*v*, 1.0 mL/min, 254 nm) major t_R = 15.65 min and minor t_S = 21.80 min

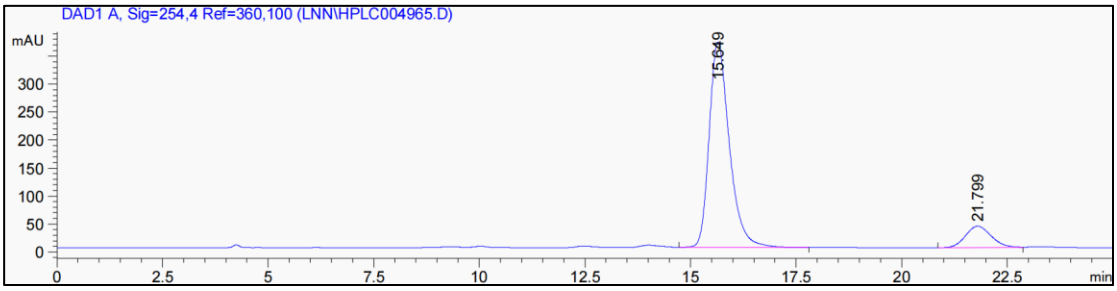

Peak	Ret. Time [min]	Type	Width [min]	Area [Mau*s]	Height [mAU]	Area %
1	15.649	BB	0.5047	1.21663e4	367.05542	88.5172
2	21.799	BB	0.6476	1578.25903	37.91838	11.4828

Racemic

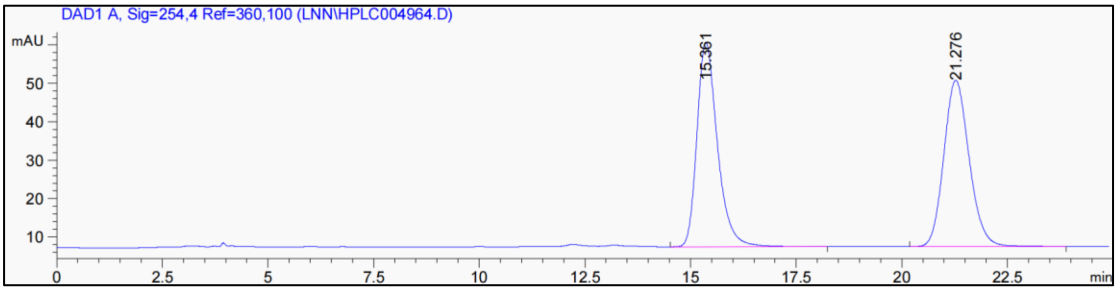

Peak	Ret. Time [min]	Type	Width [min]	Area [Mau*s]	Height [mAU]	Area %
1	15.361	BB	0.5115	1793.25806	53.16837	49.8369
2	21.276	BB	0.6470	1804.99841	43.23879	50.1631

(*S*)-2-(3-(6-Methoxy-1*H*-indol-3-yl)-3-(2-methoxyphenyl)propanoyl)pyridine 1-oxide (3ah)

ee 38%, HPLC analysis: chiralpak AS-H (*i*-PrOH/hexane = 40:60, *v*/*v*, 1.0 mL/min, 214 nm) major t_R = 18.51 min and minor t_S = 23.56 min

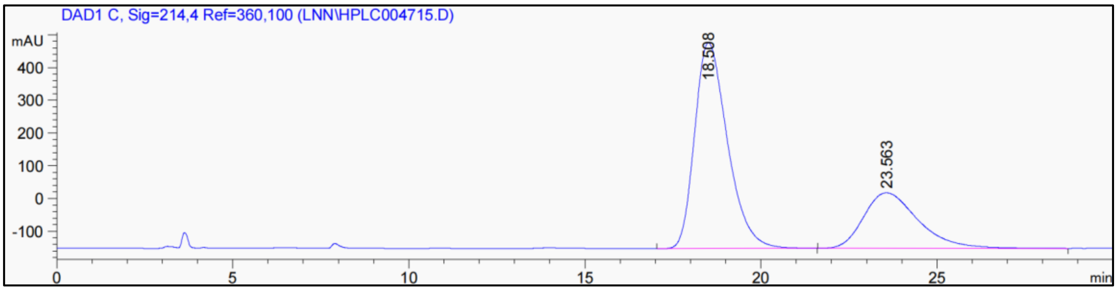

Peak	Ret. Time [min]	Type	Width [min]	Area [Mau*s]	Height [mAU]	Area %
1	18.508	BB	0.9978	4.07595e4	629.14014	68.9794
2	23.563	BB	1.6209	1.83299e4	169.60524	31.0206

Racemic

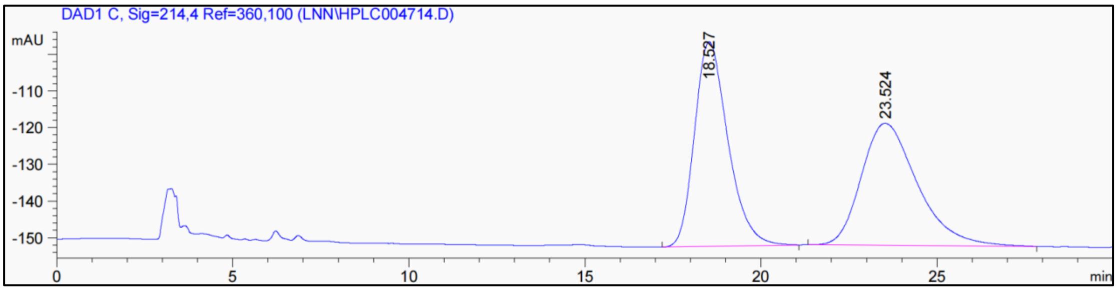

Peak	Ret. Time [min]	Type	Width [min]	Area [Mau*s]	Height [mAU]	Area %
1	18.527	BB	1.0050	3676.58789	55.77639	49.9645
2	23.524	BB	1.5716	3681.81812	33.25718	50.0355

(*S*)-2-(3-(2-Fluorophenyl)-3-(6-methoxy-1*H*-indol-3-yl)propanoyl)pyridine 1-oxide (3ai)

ee 53%, HPLC analysis: chiralpak AD-H (*i*-PrOH/hexane = 30:70, *v*/*v*, 1.0 mL/min, 254 nm) major t_R = 23.97 min and minor t_S = 38.42 min

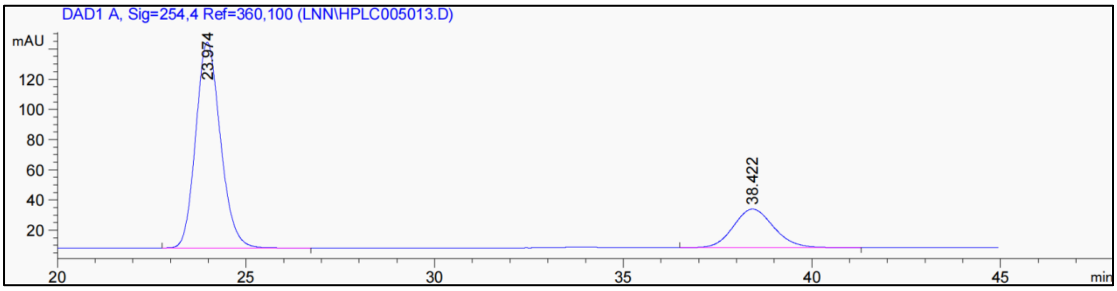

Peak	Ret. Time [min]	Type	Width [min]	Area [Mau*s]	Height [mAU]	Area %
1	23.974	BB	0.6997	6180.42090	136.11508	76.6395
2	38.422	BB	1.1371	1883.85144	25.46409	23.3605

Racemic

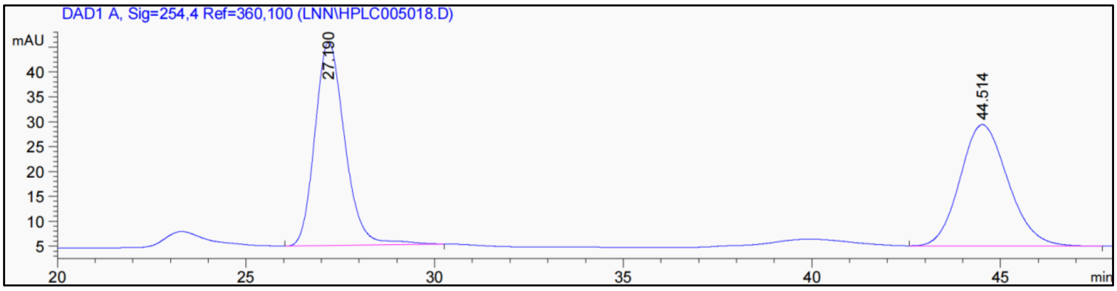

Peak	Ret. Time [min]	Type	Width [min]	Area [Mau*s]	Height [mAU]	Area %
1	27.190	BB	0.8521	2254.97119	40.96994	51.0881
2	44.514	BB	1.3358	2158.91211	24.40987	48.9119

(*S*)-2-(3-(2-Chlorophenyl)-3-(6-methoxy-1*H*-indol-3-yl)propanoyl)pyridine 1-oxide (3aj)

ee 44%, HPLC analysis: chiralpak AD-H (*i*-PrOH/hexane = 40:60, *v*/*v*, 1.0 mL/min, 254 nm) major t_R = 14.79 min and minor t_S = 19.25 min

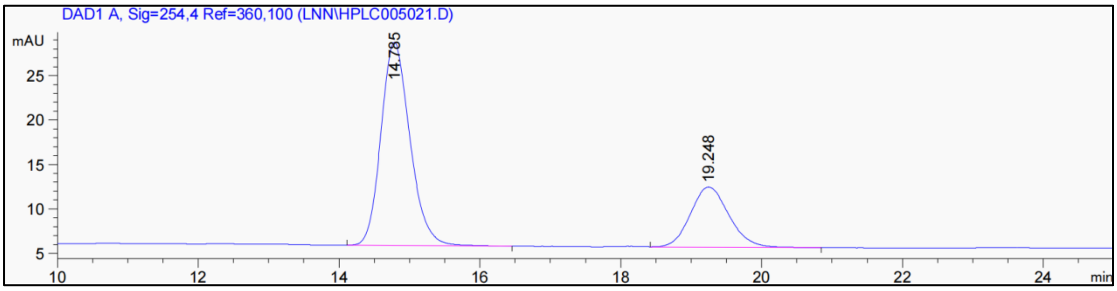

Peak	Ret. Time [min]	Type	Width [min]	Area [Mau*s]	Height [mAU]	Area %
1	14.785	BB	0.4408	652.43152	22.87374	71.8230
2	19.248	BB	0.5901	255.95598	6.75457	28.1770

Racemic

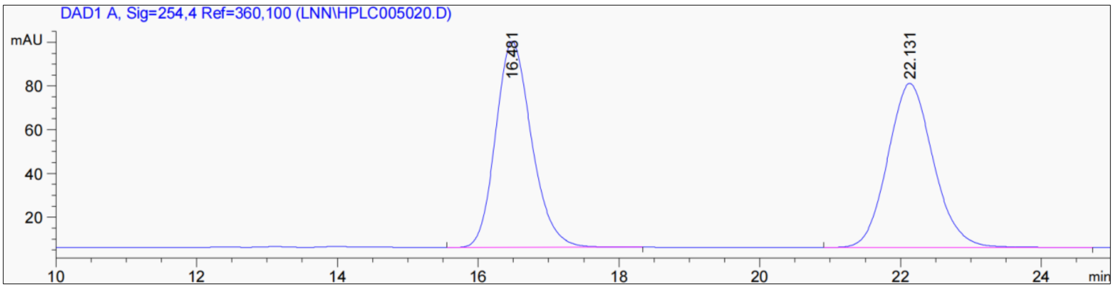

Peak	Ret. Time [min]	Type	Width [min]	Area [Mau*s]	Height [mAU]	Area %
1	16.481	BB	0.5458	3343.04468	94.24673	49.8342
2	22.131	BB	0.6974	3365.29053	75.01736	50.1658

(*S*)-2-(3-(2-Bromophenyl)-3-(6-methoxy-1*H*-indol-3-yl)propanoyl)pyridine 1-oxide (3ak)

ee 41%, HPLC analysis: chiralpak AD-H (*i*-PrOH/hexane = 40:60, *v*/*v*, 1.0 mL/min, 254 nm) major t_R = 13.40 min and minor t_S = 17.21 min

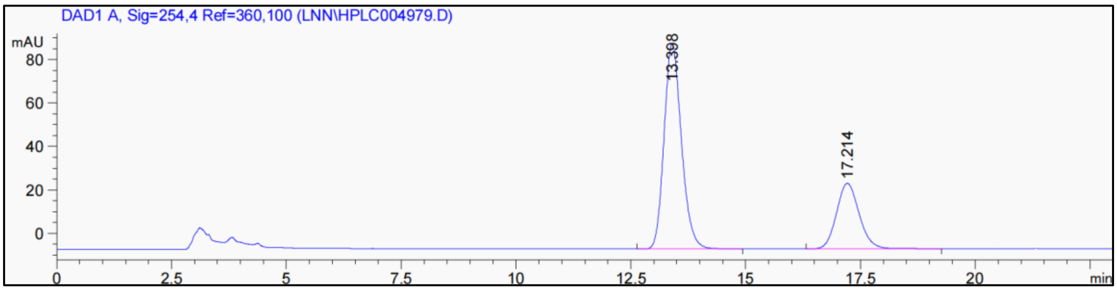

Peak	Ret. Time [min]	Type	Width [min]	Area [Mau*s]	Height [mAU]	Area %
1	13.398	BB	0.4065	2485.00488	94.57873	70.5857
2	17.214	BB	0.5305	1035.54346	30.16697	29.4143

Racemic

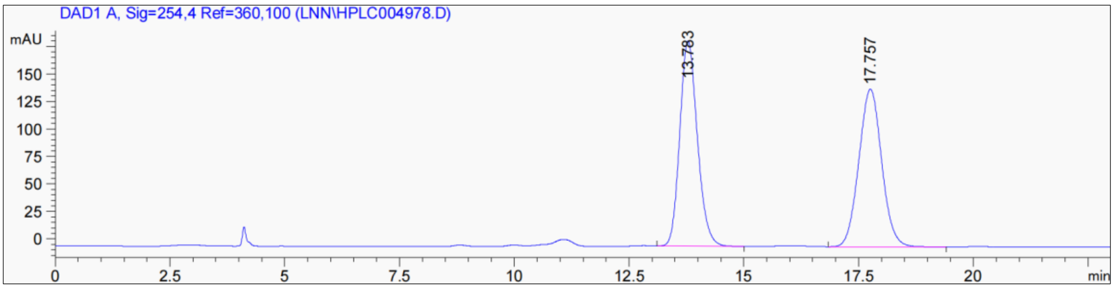

Peak	Ret. Time [min]	Type	Width [min]	Area [Mau*s]	Height [mAU]	Area %
1	13.783	BB	0.4102	4965.35547	186.75343	50.0647
2	17.757	BB	0.5352	4952.51758	143.31845	49.9353

(*R*)-2-(3-(6-Methoxy-1*H*-indol-3-yl)-3-(naphthalen-2-yl)propanoyl)pyridine 1-oxide (3al)

ee 60%, HPLC analysis: chiralpak AD-H (*i*-PrOH/hexane = 40:60, *v*/*v*, 1.0 mL/min, 254 nm) major t_R = 20.71 min and minor t_S = 23.55 min

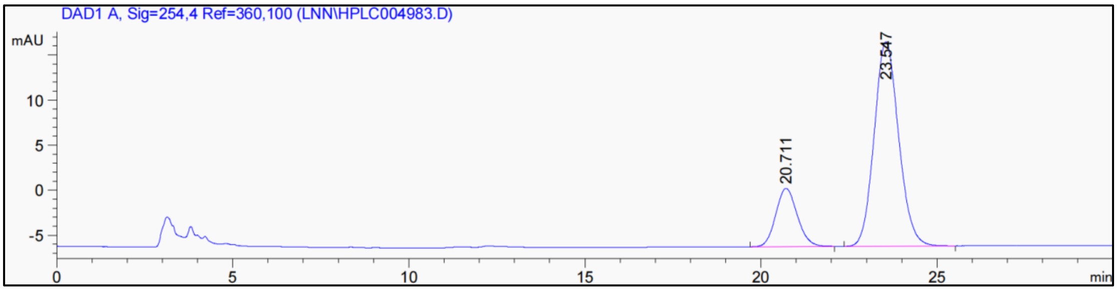

Peak	Ret. Time [min]	Type	Width [min]	Area [Mau*s]	Height [mAU]	Area %
1	20.711	BB	0.6554	273.82864	6.44823	20.0235
2	23.547	BB	0.7431	1093.70496	22.66083	79.9765

Racemic

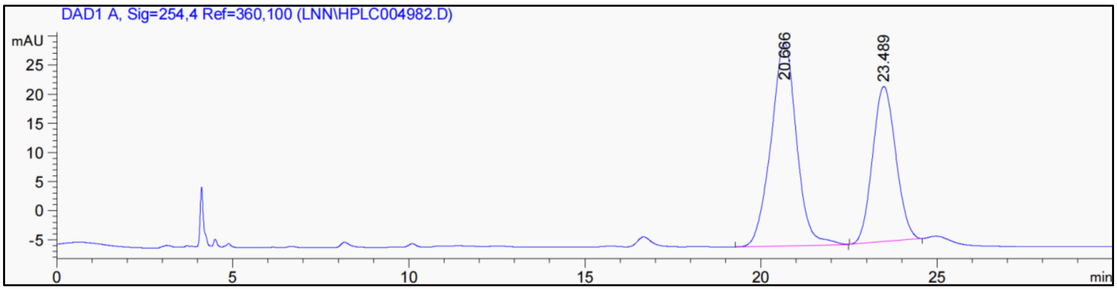

Peak	Ret. Time [min]	Type	Width [min]	Area [Mau*s]	Height [mAU]	Area %
1	20.666	BB	0.7535	1755.41174	34.97658	58.9788
2	23.489	BB	0.7146	1220.93359	26.64409	41.0212

(*S*)-2-(3-(Furan-2-yl)-3-(6-methoxy-1*H*-indol-3-yl)propanoyl)pyridine 1-oxide (3am)

ee 41%, HPLC analysis: chiralpak AS-H (*i*-PrOH/hexane = 30:70, *v*/*v*, 1.0 mL/min, 214 nm) major t_R = 29.72 min and minor t_S = 32.99 min

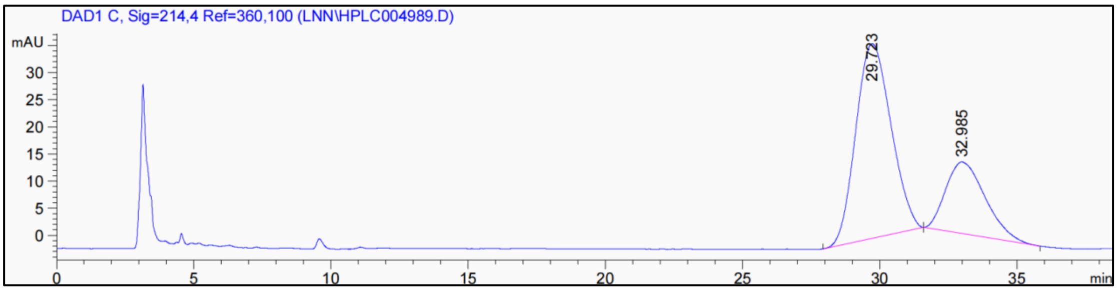

Peak	Ret. Time [min]	Type	Width [min]	Area [Mau*s]	Height [mAU]	Area %
1	29.723	BB	1.3782	3201.11377	35.90168	70.6648
2	32.985	BB	1.2694	1328.88391	13.21216	29.3352

Racemic

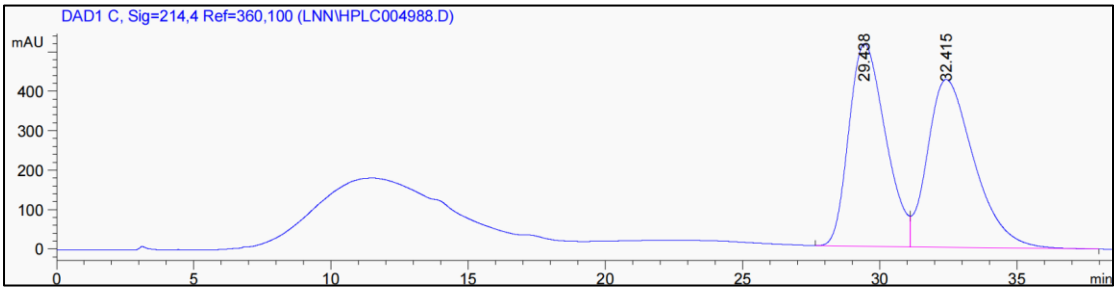

Peak	Ret. Time [min]	Type	Width [min]	Area [Mau*s]	Height [mAU]	Area %
1	29.438	BV	1.4467	4.76465e4	513.39673	48.6579
2	32.415	VB	1.6988	5.02749e4	425.40524	51.3421
